# Harnessing the copper surface for direct mechanocatalysis: a case study on mechanochemical sulfonylurea synthesis

**DOI:** 10.1039/d5sc04099j

**Published:** 2025-09-09

**Authors:** Kathleen R. Floyd, Lizette S. Mella, Ryan W. Kwok, Mackenzie Gray, Edward J. Broker, Mateusz Marianski, Tomislav Friščić, James D. Batteas

**Affiliations:** a Department of Chemistry, Texas A&M University College Station TX 77843-3255 USA; b Department of Chemistry, Hunter College New York NY 10065 USA; c PhD Program in Chemistry, Graduate Center of the City University of New York New York NY 10016 USA; d School of Chemistry, University of Birmingham Edgbaston Birmingham B152TT UK t.friscic@bham.ac.uk; e Department of Materials Science and Engineering, Texas A&M University College Station TX 77843-3127 USA batteas@chem.tamu.edu

## Abstract

Direct mechanocatalysis has arisen as a promising tool to achieve synthetic transformations by utilizing reaction vessels and/or media made of a material capable of acting as a source of catalysis. One common metal utilized for this purpose is copper, the surface of which is not a static environment but rather undergoes many transformations (particularly upon exposure to water and oxygen). Here we have utilized in-house developed equipment for work under controlled atmosphere milling, including a composite milling jar design that enables investigating the behaviour of the copper surface in either impact- or shear-dominated milling regimes. We exploit the unique sensitivity to Cu(ii) species of the mechanocatalytic coupling of isocyanate and sulfonamide to form the sulfonylurea diabetic drug tolbutamide to establish methods to control the copper surface composition and reveal the factors that influence copper transformation into different states during milling. We reveal the active catalyst formed through direct mechanocatalysis to be a hydroxylated copper species and demonstrate that the reaction proceeds *via* surface wear and subsequent catalyst formation. Any initial surface oxide is observed to be insignificant to the overall process. The use of the composite reaction jar further revealed that wear dominates from the end regions of the vessel, undergoing primarily impact forces. Based on these finding and density functional theory (DFT) calculations, we present a reaction mechanism which explains the different yields of *in situ* generated Cu(OH)_2_ and a traditional CuCl_2_ pre-catalyst. These results highlight the importance of systematic investigations of surface characteristics for understanding and controlling direct mechanocatalysis and demonstrate methods to realize these goals.

## Abstract 2

Surface Cu is mechanically converted to Cu(ii)-OH species *via* water and oxygen to catalyse the mechanochemical synthesis of tolbutamide.

## Graphical abstract

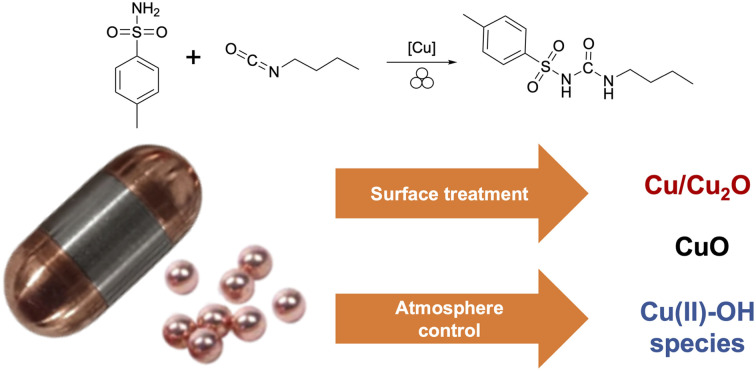

## 1 Introduction

Mechanochemistry, the application of physical force to drive chemical transformations, has arisen as a powerful green synthetic methodology for chemical synthesis. By performing reactions in a solid state rather than in solvents, mechanochemical syntheses can achieve enhanced product yields and purity, exhibit decreased reaction times, and display lower energy demands.^1–3^ Examples of such reactions include drug synthesis,^4,5^ polymer formation,^6^ industrial gas-phase processes,^7–9^ nanomaterial manufacture,^10^ MOF synthesis,^11^ waste processing,^12^ and mechanical alloying.^13,14^

In a mechanochemical reactor, energy for the chemical reaction is imparted to reagent mixtures by transducers of mechanical energy in the form of a mortar and pestle, grinding media such as milling balls (in the cases of planetary and vibratory milling),^15^ co-rotating screws (as in twin screw extrusion),^16^ or by the colliding reagents themselves (as in resonant acoustic mixing).^17^ As a result, the material of the reaction vessel often plays an important role in governing the forces experienced by reagents due to the associated mechanics,^18,19^ but the surface chemistry associated with the vessel plays a critical, yet frequently overlooked, role.^20^ For instance, the most often cited mechanochemical reaction which yields mercury *via* grinding of cinnabar in the presence of vinegar uses a copper surface on a mortar and pestle to promote the process.^21,22^ It has even been noted that mere fingerprints left on the jar surface can alter the outcomes of mechanochemical transformations.^23^

This process of direct mechanocatalysis, which uses metal additive surfaces (like foils, powders, or vials) as a source of catalyst, provides an attractive alternative to using the often expensive, synthetically-intensive, and complex organocatalytic methods currently available.^24–28^ Catalysts in traditional solvent-based synthetic processes have poor recyclability (only 18 metals utilized in industry have end-of-life recycling rates over 50%), limited shelf lives, varying quality, and generally require an inert atmosphere to achieve high conversions.^28,29^ In contrast, direct mechanocatalysis should, in principle, simplify catalyst addition, removal, and reuse, often in ambient environments.^24–28^ This speaks directly to the advantages of this technique and should foster its further development. Most studies so far focused on achieving synthetic utility, examples of which include sulfonylurea couplings with brass balls,^30^ cyclopropanation reactions with silver and copper foils in stainless steel jars,^27,31,32^ cycloadditions of alkynes with nickel pellets,^33^ palladium- and/or copper-catalysed Suzuki–Miyaura, Sonogashira, Buchwald–Hartwig,^25,34–42^ as well as azide–alkyne cycloaddition (CuAAC) reactions.^43^ A direct mechanocatalysis reaction protocol for twin screw extrusion was also demonstrated by the Borchardt group, who used palladium coated screws for continuous Suzuki–Miyaura reactions.^44^

In contrast, only a small number of studies attempted to analyse what is driving the direct mechanocatalysis by looking at surfaces and their degradation, or by identifying the catalytically active species.^25,26,45,46^ Despite being one of the most utilized metals for direct mechanocatalysis, the changes to the copper surface during the reaction remain relatively underexplored, with most mechanistic and detailed studies focusing on palladium instead.^25,44,47,48^ Studies of copper-based direct mechanocatalysis strategies have so far focused largely on the Glaser and CuAAC coupling reactions, where the formation of surface species, most likely a copper acetylide, was found to be important for catalysis.^43^ Oxidized copper metal derivatives and surface bound species have their own unique reactivities, demonstrating significant catalytic capability for a number of mechanochemical transformations, including sonocatalysis (where they uniquely serve to selectively control oxidation for biomass conversion to critical compounds under specific atmospheres).^49–51^ Thus, the current treatment of the copper metal as the catalyst in synthetic literature disguises a more complex reality. Exploration of a number of parameters, such as the structure and oxidation of the copper surface, identification of catalytic species, and how these could be controlled to improve the efficiency of direct mechanocatalysis strategies and access their economic utility is necessary.

**Fig. 1:**
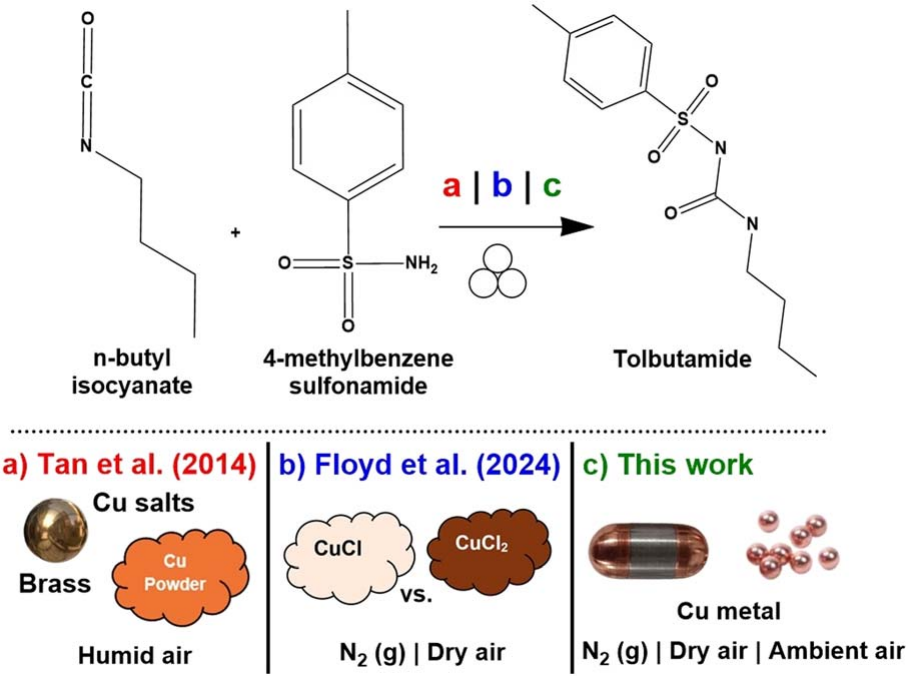
Recent investigations of copper-catalysed tolbutamide synthesis showing (a) initial report with direct mechanocatalysis [10 mm brass ball and liquid assisted grinding (LAG) with CH_3_NO_2_ (*η* = 0.25) or Cu powder (10 mol%, neat, 10 mm stainless steel ball) in a 10 mL stainless steel jar, (b) recent studies of oxidation state dependence [CuCl and CuCl_2_ (1–5 mol%)], 25 mL stainless steel jar, 15 mm stainless steel ball], and (c) this work: examinations of copper metal transformations in different atmospheres. All transformations performed in a Retsch MM 400 ball mill for 2 hours at 30 Hz.

To address this gap, we examined the copper-catalysed mechanochemical synthesis of sulfonyl-(thio)urea-based antidiabetic drugs, which serves as one of the earliest examples of direct mechanocatalysis (as shown in Fig. 1a).^27^ Previous studies demonstrated that this transformation is highly sensitive to the copper oxidation state, atmosphere, and catalyst loading, but how these factors affect the mechanism of the transformation remains unclear.^30,46,52^ We exploit the unique sensitivities of this reaction to probe the chemically relevant transformations of the copper surface under a variety of atmospheric conditions to obtain critical knowledge for mechanochemists hoping to employ direct mechanocatalysis with copper. Specifically, we find that the copper metal acts as a pre-catalyst for this reaction, transforming *in situ* to catalytically active hydroxylated copper species (herein designated as Cu(ii)-OH), given the right atmospheric conditions. Moreover, we report that the copper surface characteristics can be easily tuned by pre-treatment and maintained in lower oxidation states by atmosphere control. The condition of the initial copper surface is inconsequential, as even a robust oxide layer is easily stripped away during milling, leaving the bulk metal and the atmosphere (oxygen and water vapour) as the predominant factors driving further catalyst formation. We validate the identity of the catalyst through a combination of experimental studies and DFT calculations which reveal the mechanism of this transformation and enable direct comparison between the direct mechanocatalysis technique and traditional copper salt catalysts.

## 2 Materials and Methods

Chemicals were purchased from Alfa Aesar, Thermo Scientific, BeanTown Chemical, Strem Chemicals Inc., or Sigma Aldrich and used without further purification (see Section 1, SI).

### 2.1 X-ray photoelectron spectroscopy (XPS)

XPS data were obtained using a SPECS EnviroESCA instrument operating under traditional high vacuum pressure. Spectra were processed and fit with peak models developed by Biesinger *et al.*^53^ Copper samples were kept under nitrogen except when loading them into the instrument for analysis (∼1–3 min process, an insufficient time for significant oxide formation). To ensure that the potential effects of reduction of the copper surface by X-rays were minimized, Cu(2p^3/2^) and Cu LMM (Auger electron spectrum that arises from the electronic transitions within the L shell of an atom) spectra were recorded first, followed by the collection of other XPS data.^53^

### 2.2 Nuclear magnetic resonance (NMR)

^1^H NMR spectra were obtained with either a Bruker Advance Neo 400 instrument equipped with a 400 MHz Ascend magnet, an automated tuning 5 mm broadband iProbe, and a 60 position SampleXpress sample changer; a Bruker Advance Neo 500 instrument equipped with a Bruker Avance Neo console, a 500 Hz Ascend magnet, an automated tuning 5 mm broadband iProbe, and a 16 position SampleXpress Lite sample changer; or a Varian VNMR 500 equipped with a Varian VnmrS console, a 500 Hz Oxford magnet, and 5 mm 1H [X] broadband and [1H/19F] [X] switchable probes. The instrument was chosen based on availability to expedite data acquisition following reaction completion. ^13^C NMR spectra for product verification were obtained on a Bruker Avance III console equipped with a 500 MHz Oxford magnet, an automated tuning 5 mm ^1^H/^13^C/^15^N cold probe, and a 24 position SampleCase sample changer.

### 2.3 Inductively coupled plasma mass spectrometry (ICP-MS)

#### 2.3.1 Instrumental conditions

A PerkinElmer NexION 300D ICP-MS equipped with a 4DXCi autosampler (Elemental Scientific Inc., USA) was used. The ICP-MS incorporated a standard quartz torch and injector, a glass cyclonic spray chamber, and a PFA concentric nebulizer. The instrument was optimized prior to the analysis with a 10 ng mL^−1^ 1% v/v HNO_3_ solution containing ^9^Be, ^115^In, ^140^Ce, and ^238^U. The nebulizer flow rate was automatically adjusted to maximize transmission of ^115^In while keeping the CeO/Ce ratio below 3%. The Sc internal standard solution (5 ng mL^−1^) was added inline *via* a mixing valve. The instrument parameters are given in Section 2.1 of the SI.

#### 2.3.2 Reagents and standard solutions

All reagents used were of a trace-metal grade, suitable for ICP-MS analyses. Chromium, iron, and copper calibration solutions were prepared from aliquots of a 10 μg mL^−1^ multi-element stock solution (IV-ICPMS-71A, Inorganic Ventures, USA). Dilutions were made with a 2% v/v HNO_3_ solution prepared from Type I water (Milli-Q Integral 5, Millipore SAS, France) and trace metals grade nitric acid (Fisher Chemical, USA).

#### 2.3.3 Methods

All sample preparation was carried out in metal-free, 15 mL and 50 mL polypropylene (PP) centrifuge tubes (VWR, USA). Diluent was prepared in 1 L, acid-leached polyethylene bottles. An aliquot of approximately 35 mg of each sample was weighed and digested in a mixture of 2 mL of concentrated nitric acid and 0.5 mL of 35% hydrogen peroxide with heating at 80 °C for 4 h. When no visible solids remained, the digest was cooled to room temperature and Type I water was added to make the final mass of each up to 35 g. The total mass of each digest was recorded.

### 2.4 Liquid chromatography mass spectrometry (LC-MS)

Electrospray ionization (ESI) for product verification was obtained on a Thermo Fisher Scientific QE Focus instrument.

### 2.5 Custom milling jars

To enable milling under strictly controlled atmospheres, we have previously employed in-house designed air-tight milling jars manufactured from stainless steel (see Section 2.2, SI).^46^ Briefly, these jars were machined from stainless steel and use KF-25 flange vacuum joints, which can be sealed with a KF-25 toggle clamp and locking pin. We have also extended this setup to enable studies isolating the catalytic material to different jar regions by making 10 mL inserts of copper and stainless steel that fit within the 25 mL hermetically sealable body (see Section 2.3, SI). By mixing insert caps and cylindrical bodies of different materials, catalytically active metal can be isolated to different jar regions.

### 2.6 Milling surface preparation

Milling jars and balls made of stainless steel were cleaned with stainless steel wool using Alconox Detergent cleaning concentrate and water, rinsed with nanopure H_2_O (18.2 MΩ cm, Barnstead) followed by EtOH, and dried under streaming N_2_.^46^ Copper pieces were thrice submersed in aqueous citric acid (11.1% w/v) for 3 minutes and rinsed in nanopure H_2_O (18.2 MΩ cm, Barnstead) followed by a single rinse with 200 proof EtOH, drying under streaming N_2_, and, when necessary, oxidized under UV ozone for 45 min.^54^ For larger bulk copper insert pieces, a Fischer Scientific FS30D ultrasonic mixer was utilized during the citric acid soaks to ensure complete removal of oxide as it was observed tarnish was more difficult to remove from these samples by simple soaking. When not in use or following milling reactions, prepared copper surfaces were removed from jars and kept under inert nitrogen.

### 2.7 Optimizing oxidation of the copper surface

For trials examining the oxidation of the copper surface, Crossman Copperhead .177 calibre copper-coated precision ball-bearings (hereafter abbreviated as Cu-BBs) were cleaned as described in Section 2.6 of Materials and Methods, spread out on a glass dish, and subjected to UV ozone treatment using a Novascan PSD-UV: UV surface decontamination system. Cu-BBs were removed at 15 min intervals and stored immediately under nitrogen gas until used in reactions. Subsequently, samples were analysed *via* XPS. Oxidized copper pieces for mechanochemical reactions were exposed to UV ozone treatment for 45 minutes to optimize oxide coverage.

### 2.8 Reactions under controlled atmosphere with Cu-BBs

For studies of copper catalysis, Crosman Copperhead .177 calibre copper-coated precision BBs (hereafter abbreviated Cu-BBs) were chosen as a cost-effective (0.66 ¢ (USD)/Cu-BB), readily available material for initial investigations and examinations of surfaces *via* techniques like XPS given bulkier balls and copper pieces are more challenging to examine. Before each experiment, milling materials were cleaned as described in Sections 2.6 and 2.7 of the Materials and Methods. Subsequently, 20 Cu-BBs (4.5 mm diameter, weight ∼0.345 g per BB) were placed into the jar or jar insert along with *p*-toluenesulfonamide (214 mg, 1.25 mmol) and *n*-butyl isocyanate (140.8 μL, 1.25 mmol). The jars were then purged with 20 psi of either dried N_2_ gas or dried air in a Captair Pyramid Portable Glove Bag. An Omega RH82 hygrometer reading of 0.1% confirmed completion of purging. The concentration of O_2_ was measured using a Forensics NIST calibrated gas monitor. Upon purging, jars were sealed, placed on a Retsch MM400 mill, and milling was conducted for 2 hours at a frequency of 30 Hz. After milling, the crude product was collected from multiple jar regions, dissolved in DMSO-*d*_6_, passed through a filter pipette and crude conversion was measured by ^1^H NMR (see Section 2.2, Materials and Methods). Depending on the desired analysis, product and milled Cu-BBs could be removed under a nitrogen atmosphere following the reaction and stored under N_2_ safely away from sources of oxidation. For additional notes on product synthesis, see Section 3 of the SI. For product verification information, see Section 4 of the SI.

### 2.9 Tracking changes to the Cu-BB surfaces throughout the milling process

A stainless-steel milling jar was loaded with Cu-BBs (20, 4.5 mm diameter, weight ∼0.345 g per BB) taken directly from the manufacturer and sealed under humid atmosphere. The BBs were milled at 20 Hz in a Retsch MM 400, removed after each desired time period, cleaned with acetone, stored under N_2_, and transferred to XPS for further analysis (see Section 2.1, Materials and Methods).

### 2.10 Reactions isolating copper metal to different jar regions

Reactions isolating copper catalyst to different jar regions were performed utilizing the system described in Section 2.5 of Materials and Methods. Before each experiment, copper and stainless-steel insert pieces were cleaned using citric acid as described in Section 2.6 of Materials and Methods. Reaction jars were loaded with a SS ball (12.7 mm, ∼8.54 g), *p*-toluenesulfonamide (428 mg, 2.5 mmol), and *n*-butyl isocyanate (278.5 μL, 2.5 mmol). The jars were loaded under humid ambient atmosphere and placed on a Retsch MM400 mill. Milling was conducted for 2 h at a frequency of 30 Hz. After milling, crude product was collected from multiple jar regions, dissolved in DMSO-*d*_6_, passed through a filter pipette, and crude conversion measured by ^1^H NMR (see Section 2.2, Materials and Methods). Additional crude powder was removed from the vials immediately upon reaction conclusion and analysed through ICP-MS (see Section 2.3, Materials and Methods).

### 2.11 Examinations of reactivity with different copper-based catalysts in various atmospheres

Before each experiment, stainless steel 25 mL atmospherically sealed jars were cleaned (see Sections 2.5 and 2.6, Materials and Methods). Subsequently, reaction jars were loaded with a stainless-steel ball (15 mm, 13.4 g), *p*-toluenesulfonamide (214 mg, 1.25 mmol), *n*-butyl isocyanate (140.8 μL, 1.25 mmol), and the copper-based catalyst (5 mol% loading or varies in the case of CuO). The reactions were conducted either neat, or with addition of water (10 μL). The jars were then either: (a) purged with 20 psi of dried N_2_ gas, (b) purged with dried air in a Captair Pyramid Portable Glove Bag, or (c) placed directly on the mill under humid ambient atmosphere. As in Section 2.8 of Materials and Methods, complete purging or humidity was confirmed by a hygrometer reading of 0.1% or ∼30–60%, respectively, and the concentration of O_2_ was measured using a Forensics NIST calibrated gas monitor. Upon purging, jars were sealed, placed on a Retsch MM400 mill, and milling was conducted for 2 h at a frequency of 30 Hz. After milling, the crude product was collected from multiple jar regions, dissolved in DMSO-*d*_6_, passed through a filter pipette, and crude conversion was measured by ^1^H nuclear magnetic resonance spectroscopy (NMR) (see Section 2.2, Materials and Methods). For additional notes on product synthesis, see Sections 3 and 4 of the SI.

### 2.12 Density-functional theory (DFT) calculations on reaction mechanism

We conducted geometry optimizations to obtain the structures of all minima and transition states at the PBE0+D3/6-311+G(d,p) level of theory *via* Gaussian 16, Revision B.01, Frisch, M. J. *et al.* Gaussian, Inc., Wallingford CT, 2016. DFT functionals, such as PBE0,^55^ B3LYP,^56–58^ and M06-L^59,60^ have all been used in the literature to successfully explain the role of copper(i) and copper(ii) complexes in organic reactions. These minima and transition states were then verified using frequency calculations. In order to model the dielectric environment within the ball-mill reactor,^61^ we choose a dielectric constant of *ε* = 8.94 (phenyl isocyanate) using the Polarizable Continuum Model (PCM) as implemented in Gaussian 16.

## 3 Results and discussion

Since the formation of tolbutamide is highly sensitive to the presence of copper(ii) species, it offers a unique platform to probe the changes to the copper surface during milling (see Fig. 1).^30,46^ To do this, we first learned how to characterize and control the composition of the copper surface, and then created surfaces of known composition and probe the changing surface chemistry and associated overall % conversion in humid, dry, and nitrogen atmospheres. Sensitivity of the conversion to atmospheric conditions helped us to identify the active catalyst, Cu(OH)_2_. We then compared it to traditional alternatives using experimental and computational methods, resulting in a greater understanding of the mechanisms underlying mechanochemical and direct mechanocatalytic transformation.

### 3.1 Step 1: controlling the characteristics of the copper surface

We began by exploring the reaction driven by stainless steel BBs coated in copper. As the amount of copper catalyst present and its oxidation state have been shown to be critical for the control of reaction conversion,^46^ we first sought a method to control the surface oxidation to ensure reactions began from the same initial conditions. This requires a methodology to obtain copper as both a controlled native oxide layer and as an untarnished metal, enabling exploration from the likely initial surface states of copper prior to reaction.^54^

**Fig. 2:**
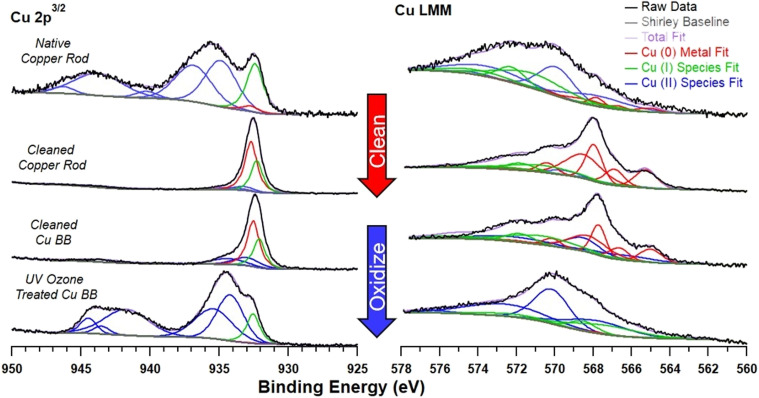
X-ray photoelectron spectroscopy (XPS) measurements of Cu LMM and Cu(2p^3/2^) peaks and associated fits with different copper metal pre-treatments demonstrating capacity to control metal surface composition.

Initial XPS of the native Cu-BBs showed that surface oxidation was inconsistent, with Cu(ii) species coverage varying from 70.1 to 90.9% between samples (see Section 5, SI for spectra, and Section 6 of the SI for verification of the experimental insignificance of surface curvature). We note that qualitatively the native metal Cu(2p^3/2^) XPS satellite feature corresponds to either Cu(OH)_2_ or CuO, depending on the sample analysed (see Fig. 2 and Section 5, SI).^53^ To ensure that the copper surface is similar across the Cu-BBs used, this initial oxide layer needed to be removed. There are a number of established methods for removing contaminant oxidation from copper surfaces in literature, along with many traditional household cleaning techniques.^54,62–64^ We sought to determine a simple and safe method, with a green footprint, that could be utilized easily across different laboratories. To test cleaning options, we cleaned cylindrical copper rods (simulating both flat and curved surfaces studied herein and in literature) with several simple methods to select a superior process (see Section 7, SI). It was found that a modified technique based on the method of Aromaa *et al.* offered the best balance of oxidation removal, low organic residue, cost, and simplicity (see Section 2.6, Materials and Methods).^54^ Upon cleaning, the copper was found to be predominantly Cu(0), with minor Cu_2_O and trace Cu(ii) species. We further developed a method to selectively oxidize the Cu-BBs to form a controlled layer of oxidized catalyst on the surface. This was achieved by exposure of cleaned BBs to ozone generated under UV irradiation (see Section 2.7, Materials and Methods). Surface coverage of 90% CuO was found to be the optimal oxide coverage attainable and resulted after 45 min of exposure to UV ozone (discussed in more detail in Section 8, SI) XPS spectra of copper samples before and after cleaning and oxidation processes are shown in Fig. 2.

### 3.2 Step 2: probing transformations of the copper surface during milling

**Fig. 3:**
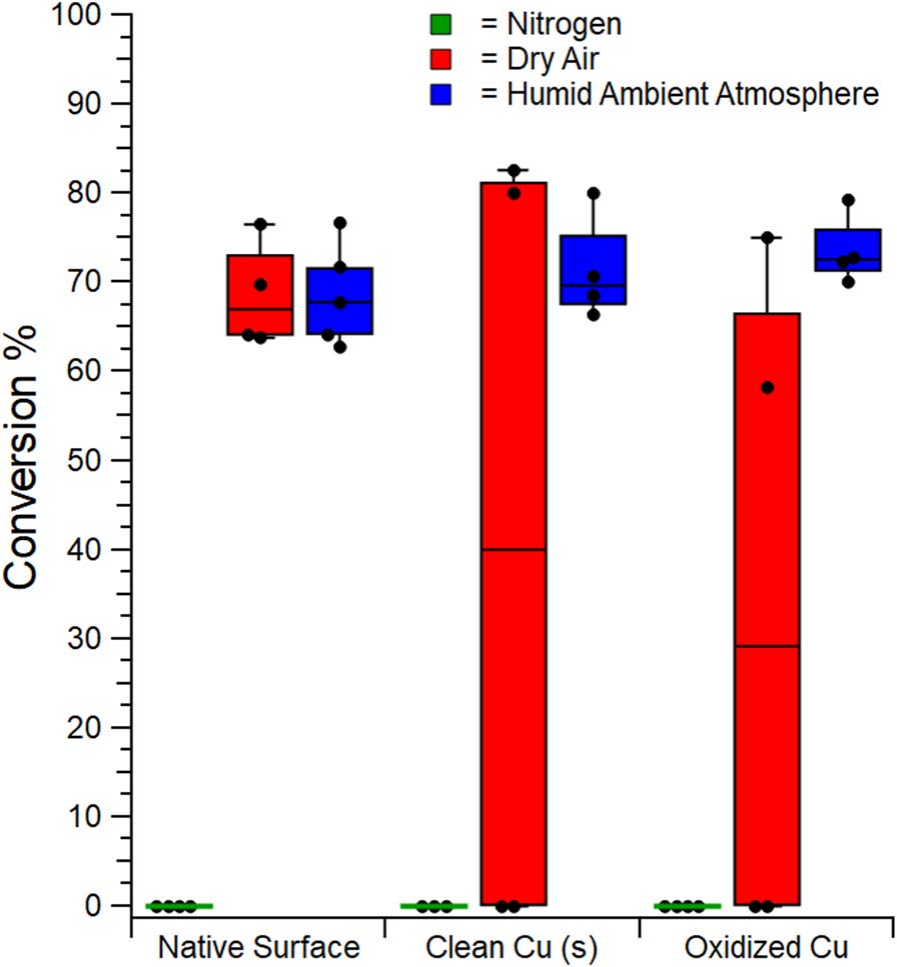
Box plots showing the mechanocatalytic performance of the copper catalysed *p*-toluenesulfonamide and *n*-butyl isocyanate coupling reaction with Cu-BBs of differently prepared surfaces under different atmospheric conditions. Milling parameters: 25 mL stainless steel hermetically sealed jars, Cu-BBs (20 count), 1.25 mmol scale, 30 Hz, 2 h.

Next, we examined the role of surface oxidation on the mechanocatalytic performance of the Cu-BBs in the synthesis of tolbutamide under different atmospheric conditions as described in Section 2.8 of the Materials and Methods. The results of these studies are summarized in Fig. 3. When run under nitrogen, reactions do not proceed significantly, regardless of the oxidation of the Cu-BB surface. In the case where the surface is clean, these results are unsurprising, given the need to oxidize Cu(0) for the reaction to proceed.^46^ The lack of reactivity in the case of native and oxidized copper surfaces, however, is surprising and suggests that surface oxide alone is insufficient to drive the transformation, no matter how extensively the surface is oxidized. This suggests that surface-bound CuO is not the catalytically active species; rather, another Cu(ii) species is responsible for the transformation.

To address this, we explored the reactivity in dry air and humid atmospheres. We noted that the repeatability of the reaction varies between dry and humid air. Humid air (50–60% RH) resulted in consistent conversions of ∼70%, while in dry air the reaction either worked well (70%), or not at all (0%), except when using the native BB surface, where reaction was consistently observed with conversions of ∼70%. Efforts to rationalize the inconsistent performance in dry air are ongoing. At present, we note the data suggests formation of a catalytically active species whose formation is: (1) dependent on the presence of water, (2) dependent on prior copper oxidation, and (3) slower from oxidized copper (leading to slightly lower maximum % conversions in dry air).

This suggests a Cu(ii)-OH species, such as Cu(OH)_2_, as the active catalyst, which might already be present in sufficient quantities on native surfaces but would not be sufficiently present on pre-treated copper surfaces. This supposition is further justified in Section 3.3. In theory, some collections of oxidized Cu BBs may have more hydroxyl or oxide coverage than others within a given batch due to different amounts of the metastable species on the surface. This could explain the high variability in yield under dry air which correlates to the variation of Cu(ii)-OH available on the surface. However, this is unlikely given the complete lack of reactivity under nitrogen which implies oxidized surface bound species are insufficient to drive the reaction alone and suggest that the active catalyst must instead be continuously generated using available water and oxygen. We suspect the reaction performs inconsistently under dry air due to water (or hydroxyl) entering the system despite our efforts and combining with the available oxygen to continuously generate the Cu(ii)-OH catalyst. The jars are hermetically sealed as evidenced by leak tests and their success with N_2_ atmosphere trials; thus, the most likely means of inconsistent water entry into the system is adsorption on the milling jar surfaces from the cleaning process. We have observed this effect in other systems and it is known that trace adsorbates can cause significant changes to reaction outcomes.^23^ At present, we do not want to destroy our jars for XPS analysis or to risk changing the underlying chemistry by heat treatment to purge the surface of adsorbates for control experiments.

At this juncture, we note that, within the standard deviation, the pre-treatment of the surface was found to be inconsequential for optimizing the % conversion with similar distributions under a humid atmosphere for all cases. These findings are in agreement with previous trials utilizing CuCl wherein LAG agents were shown to facilitate greater mixing of the solid matrix with available atmospheric oxygen to promote oxidation of the copper.^46^ This same behaviour may be achieved by milling in a humid environment. However, we judge that, within the context of direct mechanocatalysis, these results more strongly suggest a need for water to form the active catalyst from bulk copper.

**Fig. 4:**
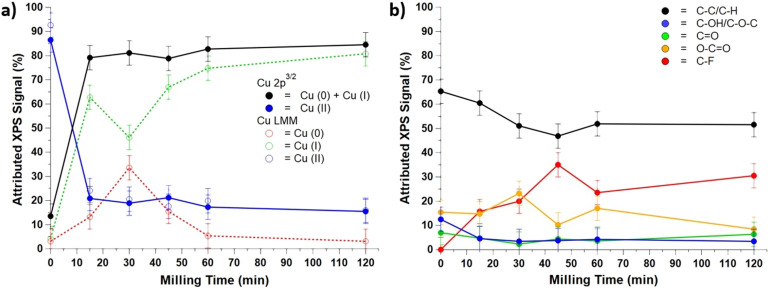
XPS surface area coverage measurements of the Cu-BBs surface (a) of Cu(2p^3/2^) and LMM peaks and (b) C(1s) peaks as a function of milling time. Milling parameters: stainless steel hermetically sealed jars, Cu-BBs (20 count), 30 Hz, 2 h, ambient atmosphere.

The CuO layer on the surface of Cu-BBs appears insufficient to drive reactivity; rather, it likely wears from the surface to reveal Cu (s). Available copper in the solid powder matrix is then converted to a more active catalytic form. To confirm this, we examined the changes to the surface by XPS utilizing native Cu-BBs milled under a humid atmosphere (see Fig. 4 and Section 9, SI for controls; and Section 10, SI for additional O(1s) XPS results). We observe that the surface of the BBs is stripped of CuO, with the coverage changing from ∼85% to ∼20% within the first 15 minutes of milling, leaving Cu_2_O to dominate the surface. The Cu_2_O continues to wear from the surface until approximately 30 minutes of milling, after which it begins to slowly build back up on the ball surface. The C(1s) signal suggests C–F bonds (likely from an industrial treatment of the metal surface before Cu deposition) slowly appearing and building up through the course of milling, suggesting the surface continues to wear throughout the milling process. This agrees with the general finding that the reaction is facilitated by copper that wears off the surface and converts to an active catalyst rather than *via* surface-bound intermediates. Attempts to measure the copper wear by pre- and post-milling of the BBs were unsuccessful due to insignificant sensitivity to mass changes, but visual dulling and “greying” of the BBs and shiny copper metal residue were observed. We measured the total Cu content in the reaction powder at different time points during the 2 h reaction using ICPMS to track Cu wear and found that copper wears off within 30 minutes of milling and the amount of wear does not vary significantly with further milling (see Section 11, SI). Further kinetic studies are complicated by poor repeatability of the reaction at short run times (see Section 11, SI).

**Fig. 5:**
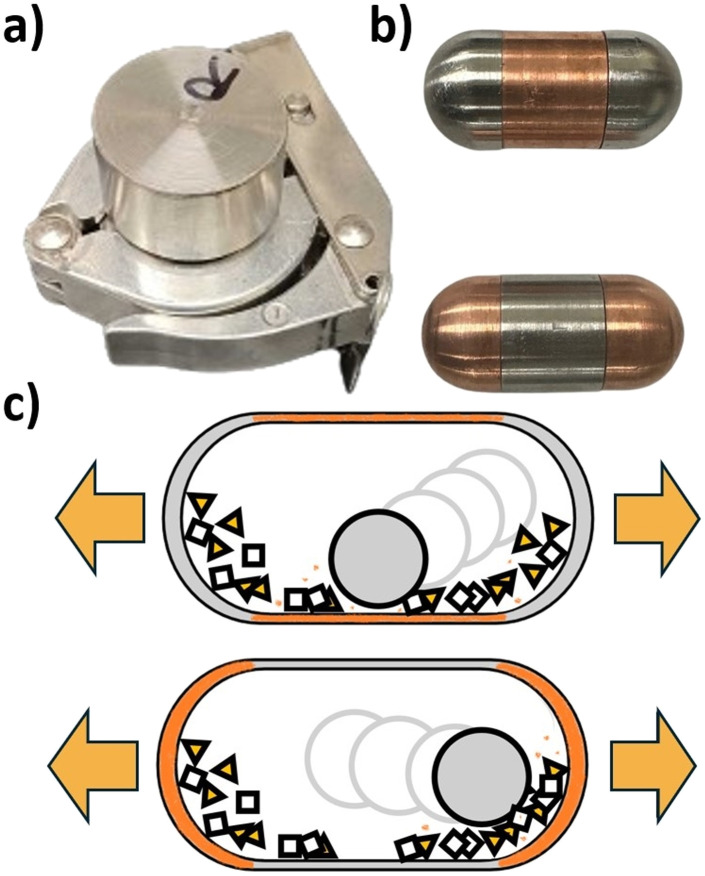
Custom milling jars for isolating copper milling media to different jar regions showing (a) outer jar holder, (b) inserts isolating copper to different regions, and (c) schematic of copper wearing from different regions during the milling process. Milling parameters: custom composite milling jars (stainless steel and copper), 15 mm stainless steel ball, 2.5 mmol, 30 Hz, 2 h.

As a means to control direct mechanocatalysis, we sought to determine the location within the milling jars where copper wear would be most prevalent during reaction, so that the catalyst amount could in principle be tuned. This is particularly relevant for tolbutamide synthesis, as it has been shown that increasing catalyst amount above a certain minimal threshold is detrimental to conversion due to a dilution effect.^46^ To explore this, we designed custom milling jar inserts which facilitated the construction of a composite milling jar, in which the catalytically-active copper metal surface can be placed either only at the end caps, or only in the cylindrical body of the jar (see Fig. 5, also Section 2.5, Materials and Methods). Notably, these two regions of the milling jar were found to undergo different types of mechanical stress, with the end caps experiencing more impact, and the central part of the jar experiencing more shear.^65^ Consequently, the use of the composite milling jar effectively permits isolation of the surface responsible for catalytic activity in either the impact- or the shear-dominated regime of ball-milling. Using the composite milling jar, the reactions were performed under a humid atmosphere (see Section 2.10, Materials and Methods). Analysis of the resulting powder mixtures showed that placing a copper surface at the vessel ends leads to more copper wear per unit surface area (85.5 μg mm^2^ g^−1^), compared to the case in which a copper surface was located in the cylindrical body of the jar (16.4 μg mm^2^ g^−1^). Despite the significant difference in copper loss, both reaction vessel designs led to similar conversions of ∼80%, likely due to sufficient excess available copper (see Section 11, SI). This suggests that copper is more readily removed from areas where impact forces predominantly occur and less readily removed when undergoing more shearing forces. Furthermore, copper loss and catalyst amount are correlated to surface area and placement in the jar. In future work, we aim to explore this effect further, but we note that this suggests that the design of milling vessels to optimize catalyst endurance or wear quantity is feasible.

### 3.3 Step 3: isolating Cu(ii)-OH species as the surface derived catalyst

We next turned to determining the active catalyst by investigating different copper oxides that could form from a combination of water, oxygen, and copper metal under different atmospheres. The methodology is described in Section 2.11 of Materials and Methods. A summary of the results is presented in Table 1. The reaction has previously been performed successfully under humid atmosphere using a number of copper based catalysts including CuCl, CuCl_2_, Cu_2_O, CuCl_2_·2H_2_O, Cu(acetate)_2_·H_2_O, and copper nanopowder.^30,46,66^ Moreover, it has been generally observed that ligands can play a critical role in controlling the mechanochemical reactivity of base metals.^47,67^

**Table 1:** Conversion to tolbutamide (in %) when using different copper species as catalytic additive under varying atmospheric conditions

Catalyst	Catalyst loading (% mol)	Atmosphere	Conversion^a^ (%)
CuCl_2_	5	Nitrogen	75 ± 7^b^
CuCl_2_	5	Dry air	75 ± 3^b^
CuCl_2_	1	Dry air	92 ± 1^b^
CuO	1	Dry air	0 ± 0
CuO	5	Humid	0 ± 0
CuO	10	Humid	19 ± 14^c^
CuO + 10 μL H_2_O	30	Nitrogen	0 ± 0
Cu_2_O	5	Dry air	71 ± 12
Cu_2_O	5	Humid	82 ± 3
Cu_2_O	5	Nitrogen	0 ± 0
Cu_2_O + 10 μL H_2_O	5	Nitrogen	0 ± 0
Cu(OH)_2_	5	Nitrogen	77 ± 4
Cu(OH)_2_	1	Dry air	52 ± 5
Cu-BBs (cleaned) + 10 μL H_2_O		Nitrogen	0 ± 0
Cu-BBs (oxidized) + 10 μL H_2_O		Nitrogen	0 ± 0

a verage of two measurements.

b Results taken from ref. 46.

c Average of four measurements due to one trial with no conversion. Uncertainty is given as one standard deviation. Reaction conditions: *p*-toluenesulfonamide (214 mg, 1.25 mmol), *n*-butyl isocyanate (140.8 μL, 1.25 mmol), 15 mm diameter SS ball, 30 Hz, 2 h reaction time.

CuO was first explored using the traditional conditions from literature for high outputs of *ca.* 75–80% yields (dry air, 1 mol% loading),^46^ but no transformation occurred. Using CuO in a humid atmosphere and at higher catalyst loadings resulted in only *ca.* 19% conversion. This is considerably lower than conversion obtained with CuO on the Cu-BB surface in a humid atmosphere, where conversions typically reached 70% (see Fig. 3). Further trials adding additional water to the mixture along with higher catalyst loadings under inert atmosphere also led to no conversion. Overall, this suggests that CuO is not the primary active catalyst in this transformation and most likely plays a role of a pre-catalyst. CuO species have been shown to reduce under mechanochemical milling in the presence of a reducing agents such as *N*-heterocyclic carbenes or graphite.^68–70^ Similarly oxidation has been observed during polishing and milling of copper metal in aerobic environments.^71,72^ Thus, it is plausible conversion of CuO back to Cu_2_O or onward to other hydroxylated species is responsible for its poor reactivity in humid atmosphere at high catalyst loadings.

Next, we turned to exploring Cu_2_O as the additive. Under an inert atmosphere, the reaction did not proceed significantly, indicating that this also is not the active catalyst species. The reaction proceeded well, however, under a humid atmosphere, reaching a conversion of *ca.* 82%. High conversion of *ca.* 71% was also observed by conducting the reaction in dry air (see Table 1). Attempts to perform the reaction in the presence of Cu_2_O with added 10 μL of water under an inert atmosphere were not successful, suggesting that oxygen is necessary to convert Cu_2_O to a catalytically active form.

In humid conditions where both oxygen and water are present, copper is reported to undergo three stages of oxidation involving the formation of Cu_2_O followed by the formation of Cu(OH)_2_ which later converts to the more stable CuO. The first stage consists of the formation of a Cu_2_O layer and OH groups.^73,74^ It is known that water adsorbed on metal surfaces can undergo dissociation, thereby forming surface OH groups; oxygen enhances this process and reports differ on whether dissociation can occur in an inert enviornment.^75–77^ These OH groups then react with Cu atoms diffusing to the oxide-oxygen interface to form a Cu(OH)_2_ layer.^74,78^ Consequently, we turned to Cu(OH)_2_ as an option for the active catalyst. Using a 5 mol% catalyst loading under a nitrogen atmosphere, a 77% conversion to tolbutamide was observed (see Table 1), matching the highest observed reactivity seen in humid atmospheres with the Cu-BBs under direct mechanocatalysis conditions.

We attempted to capture the formation of Cu(OH)_2_ in the reaction powder using Powder X-ray Diffraction (PXRD), Raman spectroscopy, Fourier Transform-Infrared (FT-IR) spectroscopy, and XPS. However, we were unable to detect the formation of Cu(OH)_2_ species *via* Raman, PXRD and FTIR due to the low amounts of the catalyst present in the solid organic matrix (∼15 000 μg Cu per g powder, see Section 12, SI). While the Cu LMM peak and satellite features in the Cu 2p^3/2^ peak from XPS resemble those obtained using reference Cu(OH)_2_ powders, due to sample charging we were unable to acquire spectra that could undeniably confirm its presence (See Section 13, SI for further discussion of these results).

Nevertheless, the results presented so far support that a hydroxylated copper species is responsible for driving the transformation. To further determine which factors govern the mechanochemical formation of the hydroxylated copper from the native surface, we attempted the reaction under a nitrogen atmosphere using cleaned Cu-BBs with an additional 10 μL of water. No reaction was observed, indicating the presence of oxygen is critical to form the Cu(ii)-OH species. Identical results were seen with oxidized Cu-BBs. The necessity of oxygen may be connected to the formation of the hydroxylated layer which is dependent on the concentration of OH groups forming from the dissociation of water on the metal surface in the presence of adsorbed O atoms on the Cu_2_O/water interface.^73,74,79^

**Fig. 6:**
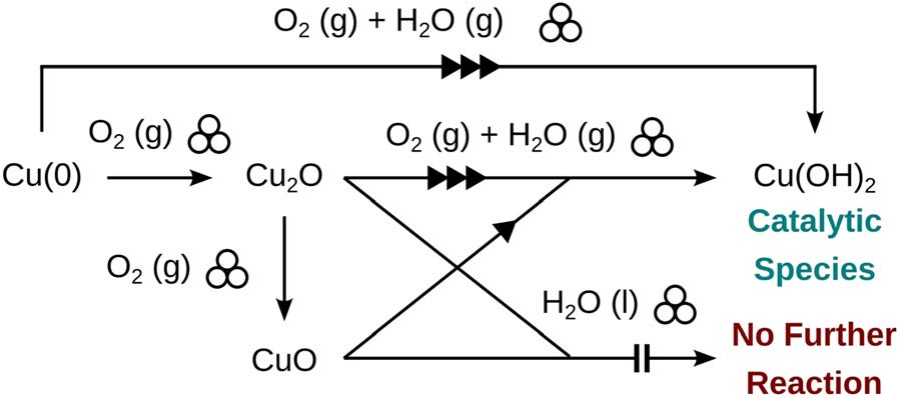
Pathways from the copper metal surface (either directly from cleaned metal or copper metal revealed after initial CuO layer wear) to the active hydroxide catalyst revealed by experimental reaction performance under different atmospheres with different catalyst precursors and Cu-BBs.

A summary of these findings and experimentally suggested routes to the active catalyst during the reaction are shown in Fig. 6.

### 3.4 Step 4. mechanism of tolbutamide formation: comparing catalyst derived from the copper surface to traditional salt catalyst

Having determined how to control the copper surface and its likely transformations, we sought to cross verify these experimental results through computational methods by determining the mechanism of the transformation through DFT calculations. We utilized Cu(OH)_2_ to compare the activity of the derived Cu(ii)-OH species with other traditionally used copper salts such as CuCl_2._ By obtaining a better understanding of the role of the derived catalyst within the transformation and the dependence of the reaction on copper transformation, we hoped to further elucidate how direct mechanocatalytic techniques may differ from using traditional copper salt catalysts. A better understanding of the fundamentals of this transformation will also be critical to realize kinetic studies and reaction scaleup in future work.

In our previous work, we were able to achieve up to ∼90% conversions at low catalyst loading using CuCl_2_ (see Table 1).^46^ We noted that reactions with direct mechanocatalysis have lower conversions in general (averaging around 75–80% conversion). This may well be due to the dilution effect by extra worn copper paralleling the effect of excessive catalyst loading observed previously.^46^ However, there may also be an inherent difference between the different copper-based active catalysts as, under identical conditions, Cu(OH)_2_ has an approximately 40% lower conversion (see Table 1). Furthermore, while the performance of CuCl_2_ is enhanced when reducing loading from 5 mol% to 1 mol%,^46^ Cu(OH)_2_ improves conversion upon increasing loading from 1 mol% to 5 mol%. This data suggests that these catalysts do not have equivalent activity, but that the conversions are only similar at high loadings due to having a sufficient excess of catalyst. To explore this effect and clarify why the transformation requires oxidized copper more generally, we turned to explorations of the mechanism informed by DFT calculations (see Section 2.12, Materials and Methods).

**Fig. 7:**
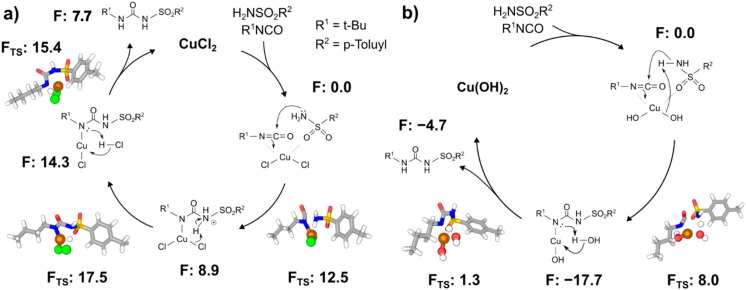
Proposed mechanisms for tolbutamide formation with (a) CuCl_2_ and (b) Cu(OH)_2_ as the active catalyst. Free energies are shown in kcal mol^−1^ from DFT.

It has been reported that the coupling between *p*-toluenesulfonamide and *n*-butyl isocyanate does not occur without the presence of a catalyst, allowing us to rule out any potential mechanisms that could proceed independently of copper.^30^ To reduce the computational cost, we investigated the reaction based on single-molecules instead of bulk surfaces. However, this assumption should be consistent with the mechanochemical wear of the surface. As such, we first considered the synthesis of tolbutamide with a CuCl_2_ catalyst. The mechanism we proposed is depicted in Fig. 7a and is similar to a mechanism suggested by Oda *et al.* on the copper-catalysed addition of alcohol to isocyanates.^80^ First the *n*-butyl isocyanate and *p*-toluenesulfonamide reactants coordinate with CuCl_2_. The nitrogen on *p*-toluenesulfonamide then attacks the electrophilic isocyanate carbon, resulting in the desired linkage. The transfer of the proton from the sulfonamide nitrogen to the isocyanate nitrogen is then facilitated by one of the chlorine ligands on CuCl_2_, thereby yielding tolbutamide and regenerating our copper catalyst. Our calculations showed that the initial coordination of CuCl_2_ with the nitrogen atom on the isocyanate and the oxygen on the sulfonamide to form a tetrahedral complex is more stable than the uncoordinated reactants by about 13.2 kcal mol^−1^. For this reason, all free energies are relative to this tetrahedral complex. The reaction with Cu(OH)_2_ was found to follow a similar mechanism. Here, after the initial coordination of the reagents to Cu(OH)_2_, the nucleophilic attack and deprotonation of the sulfonamide nitrogen occurs in a concerted fashion, rather than sequentially as was the case with CuCl_2_. The deprotonation of the sulfonamide nitrogen by an OH^−^ ligand rather than a Cl^−^ ligand results in the formation of water, which is then deprotonated to yield tolbutamide and reform our Cu(OH)_2_ catalyst.

These mechanisms agree with our experimental results. Lower conversions appear characteristic of the Cu(OH)_2_ catalyst and can be attributed to the formation of water following deprotonation of the sulfonamide nitrogen. This leads to an exceptionally stable intermediate (−17.7 kcal mol^−1^) that slows further reaction progress. By contrast, the corresponding intermediate with CuCl_2_ has an intermediate energy of about 14.3 kcal mol^−1^. The lack of catalysis by CuO can be similarly explained as the inability of the coordinated oxygen to facilitate this proton transfer. While the initial deprotonation of the sulfonamide nitrogen is possible, re-protonation of the isocyanate nitrogen by OH^−^ is extremely unlikely. We have also considered previously reported results for ZnCl_2_ and CaCl_2_, which have been shown to occur with yields of 6% and 0% respectively. Following the proposed mechanism for CuCl_2_, we conducted DFT calculations using these catalysts. In the case of ZnCl_2_, nearly every step in the mechanism was significantly higher in energy than that for CuCl_2_. In the case of CaCl_2_, all attempted transition state searches were unsuccessful, suggesting that these points do not exist. Thus, our calculations are in agreement with the reported lack of yield for these catalysts, further validating the mechanism (see Section 14, SI).^46^

## 4 Conclusions

In summary, we established an in-house method to control the composition of the copper surface and applied it to Cu-BBs which were used as an inexpensive and commercially available source of copper catalyst in the mechanochemical synthesis of tolbutamide. In tandem with atmosphere control, we were able to provide a systematic study on the role of the copper surface in the reaction by exploiting our former discovery that only copper in (II) oxidation state is catalytically active.^46^ We now reveal that the mechanocatalytic reaction is catalysed by Cu(OH)_2_, which forms *in situ* during the milling process. The formation of the active catalyst is shown to be dependent on both atmospheric oxygen and atmospheric water. Moreover, we show that the initial state of the copper surface (either as a native oxide or untarnished metal) is inconsequential to the reaction, with all significant activity happening in copper metal below the native oxide layer that wears from the surface and converts to Cu(OH)_2_. Building on this, we also investigated isolating the copper metal at different regions and found that copper wear can be mitigated by placing the copper in the central body of the jar rather than the ends. Reaction conversions are inferior to that which can be achieved by direct use of the copper(ii) chloride salt at low catalyst loadings,^81^ highlighting the critical role of ligands before reactions can occur and current limitations of the methodology. Furthermore, we proposed a mechanism for the mechanochemical Cu(ii)-catalysed C–N coupling of isocyanate and sulfonamide. DFT calculations revealed that Cu(OH)_2_ is a less ideal catalyst than CuCl_2_ for the reaction due to the formation of a highly stable intermediate. We believe the methodologies presented herein can be extended to further understand other mechanocatalytic transformations where analysis of critical factors driving synthetic activity remains an ongoing challenge.^27^

## Author contributions

Author contributions to this work according to CRediT standardised contribution descriptions are as follows: Kathleen Floyd (conceptualization, data curation, formal analysis, investigation, methodology, project administration, supervision, validation, visualization, writing – original draft, writing – review and editing), Lizette Mella (data curation, formal analysis, investigation, validation, validation, writing – original draft, writing – review and editing), Ryan Kwok (conceptualization, data curation, formal analysis, investigation, methodology, software, validation, visualization, writing – original draft, writing – review and editing), Edward J. Broker, Jr (formal analysis, writing – review and editing), Mateusz Marianski (conceptualization, project administration, formal analysis, supervision, writing – review & editing), James Batteas (conceptualization, funding acquisition, project administration, resources, supervision, writing – review & editing), Tomislav Friščić (writing – review & editing).

## Conflicts of interest

There are no conflicts to declare.

## Supplementary Material

SC-016-D5SC04099J-s001

## Data Availability

The data supporting this article has been included as part of the SI in Section 13. Supplementary information: Chemical details, notes on general procedures and methods, representative product verification data, additional XPS data, additional DFT calculations. See DOI: https://doi.org/10.1039/d5sc04099j.

## References

[cit1] O'Neill R. T., Boulatov R. (2021). The many flavours of mechanochemistry and its plausible conceptual underpinnings. Nat. Rev. Chem..

[cit2] Ardila-Fierro K. J., Hernández J. G. (2021). Sustainability Assessment of Mechanochemistry by Using the Twelve Principles of Green Chemistry. ChemSusChem.

[cit3] Fantozzi N., Volle J.-N., Porcheddu A., Virieux D., García F., Colacino E. (2023). Green metrics in mechanochemistry. Chem. Soc. Rev..

[cit4] Tan D., Loots L., Friščić T. (2016). Towards medicinal mechanochemistry: evolution of milling from pharmaceutical solid form screening to the synthesis of active pharmaceutical ingredients (APIs). Chem. Commun..

[cit5] Solares-Briones M., Coyote-Dotor G., Páez C., Zermeño-Ortega M., Contreras C., Canseco-Gonzalez D., Avila-Sorrosa A., Morales-Morales D., Germán J. (2021). Mechanochemistry: A Green Approach in the Preparation of Pharmaceutical Cocrystals. Pharmaceutics.

[cit6] Brantley J. N., Wiggins K. M., Bielawski C. W. (2013). Polymer mechanochemistry: the design and study of mechanophores. Polym. Int..

[cit7] Reichle S., Felderhoff M., Schüth F. (2021). Mechanocatalytic Room-Temperature Synthesis of Ammonia from Its Elements Down to Atmospheric Pressure. Angew. Chem., Int. Ed..

[cit8] Han G.-F., Li F., Chen Z.-W., Coppex C., Kim S.-J., Noh H.-J., Fu Z., Lu Y., Singh C. V., Siahrostami S. (2021). *et al.*, Mechanochemistry for ammonia synthesis under mild conditions. Nat. Nanotechnol..

[cit9] Bolm C., Hernández J. G. (2019). Mechanochemistry of Gaseous Reactants. Angew. Chem., Int. Ed..

[cit10] Yang L. , Moores A., Friščić T. and Provatas N., A Thermodynamics Model for Mechanochemical Synthesis of Gold Nanoparticles: Implications for Solvent-free Nanoparticle Production, arXiv, 2020, preprint, arXiv:2011.12521v4, 10.48550/arXiv.2011.12521

[cit11] Stolar T., Užarević K. (2020). Mechanochemistry: an efficient and versatile toolbox for synthesis, transformation, and functionalization of porous metal–organic frameworks. CrystEngComm.

[cit12] Guo X., Xiang D., Duan G., Mou P. (2010). A review of mechanochemistry applications in waste management. Waste Manage..

[cit13] Shuai C., He C., Peng S., Qi F., Wang G., Min A., Yang W., Wang W. (2021). Mechanical Alloying of Immiscible Metallic Systems: Process, Microstructure, and Mechanism. Adv. Eng. Mater..

[cit14] Benjamin J. S. , New Materials by Mechanical Alloying Techniques, DGM Informations-gesellschaft, 1989

[cit15] Andersen J., Mack J. (2018). Mechanochemistry and organic synthesis: from mystical to practical. Green Chem..

[cit16] Crawford D. E., Miskimmin C. K. G., Albadarin A. B., Walker G., James S. L. (2017). Organic synthesis by Twin Screw Extrusion (TSE): continuous, scalable and solvent-free. Green Chem..

[cit17] Effaty F., Gonnet L., Koenig S. G., Nagapudi K., Ottenwaelder X., Friščić T. (2023). Resonant acoustic mixing (RAM) for efficient mechanoredox catalysis without grinding or impact media. Chem. Commun..

[cit18] Nwoye E., Raghuraman S., Costales M., Batteas J., Felts J. R. (2023). Mechanistic model for quantifying the effect of impact force on mechanochemical reactivity. Phys. Chem. Chem. Phys..

[cit19] Michalchuk A. A. L., Tumanov I. A., Boldyreva E. V. (2019). Ball size or ball mass – what matters in organic mechanochemical synthesis?. CrystEngComm.

[cit20] Wu Y., Lin K., Ruan J. (2023). Control the Mechanochemical Energy of Ball Milling To Remove Surface Organic Contamination without Damaging the Integrity of the Glass. ACS Sustain. Chem. Eng..

[cit21] Marchini M., Montanari G., Casali L., Martelli M., Raggetti L., Baláž M., Baláž P., Maini L. (2024). “What makes every work perfect is cooking and grinding”: the ancient roots of mechanochemistry. RSC Mechanochem..

[cit22] Takacs L. (2013). The historical development of mechanochemistry. Chem. Soc. Rev..

[cit23] Zheltikova D., Losev E., Boldyreva E. (2023). To touch or not to touch? Fingerprint-assisted grinding of carbamazepine form III. CrystEngComm.

[cit24] Pickhardt W., Grätz S., Borchardt L. (2020). Direct Mechanocatalysis: Using Milling Balls as Catalysts. Chem. - Eur. J..

[cit25] Wohlgemuth M., Mayer M., Rappen M., Schmidt F., Saure R., Grätz S., Borchardt L. (2022). From Inert to Catalytically Active Milling Media: Galvanostatic Coating for Direct Mechanocatalysis. Angew. Chem., Int. Ed..

[cit26] Vogt C. G., Oltermann M., Pickhardt W., Grätz S., Borchardt L. (2021). Bronze Age of Direct Mechanocatalysis: How Alloyed Milling Materials Advance Coupling in Ball Mills. Adv. Energy Sustainability Res.

[cit27] Hwang S., Grätz S., Borchardt L. (2022). A guide to direct mechanocatalysis. Chem. Commun..

[cit28] Wenger L. E., Hanusa T. P. (2023). Synthesis without solvent: consequences for mechanochemical reactivity. Chem. Commun..

[cit29] Graedel T. E., Allwood J., Birat J.-P., Buchert M., Hagelüken C., Reck B. K., Sibley S. F., Sonnemann G. (2011). What Do We Know About Metal Recycling Rates?. J. Ind. Ecol..

[cit30] Tan D., Štrukil V., Mottillo C., Friščić T. (2014). Mechanosynthesis of pharmaceutically relevant sulfonyl-(thio)ureas. Chem. Commun..

[cit31] Jones A. C., Leitch J. A., Raby-Buck S. E., Browne D. L. (2022). Mechanochemical techniques for the activation and use of zero-valent metals in synthesis. Nat. Synth..

[cit32] Chen L., Leslie D., Coleman M. G., Mack J. (2018). Recyclable heterogeneous metal foil-catalyzed cyclopropenation of alkynes and diazoacetates under solvent-free mechanochemical reaction conditions. Chem. Sci..

[cit33] Haley R. A., Zellner A. R., Krause J. A., Guan H., Mack J. (2016). Nickel Catalysis in a High
Speed Ball Mill: A Recyclable Mechanochemical Method for Producing Substituted Cyclooctatetraene Compounds. ACS Sustain. Chem. Eng..

[cit34] Seo T., Ishiyama T., Kubota K., Ito H. (2019). Solid-state Suzuki–Miyaura cross-coupling reactions: olefin-accelerated C–C coupling using mechanochemistry. Chem. Sci..

[cit35] Seo T., Kubota K., Ito H. (2023). Mechanochemistry-Directed Ligand Design: Development of a High-Performance Phosphine Ligand for Palladium-Catalyzed Mechanochemical Organoboron Cross-Coupling. J. Am. Chem. Soc..

[cit36] Kubota K., Seo T., Ito H. (2023). Solid-state cross-coupling reactions of insoluble aryl halides under polymer-assisted grinding conditions. Faraday Discuss..

[cit37] Kubota K., Baba E., Seo T., Ishiyama T., Ito H. (2022). Palladium-catalyzed solid-state borylation of aryl halides using mechanochemistry. Beilstein J. Org. Chem..

[cit38] Kubota K., Endo T., Uesugi M., Hayashi Y., Ito H. (2022). Solid-State C–N Cross-Coupling Reactions with Carbazoles as Nitrogen Nucleophiles Using Mechanochemistry. ChemSusChem.

[cit39] Kubota K., Takahashi R., Uesugi M., Ito H. (2020). A Glove-Box- and Schlenk-Line-Free Protocol for Solid-State C–N Cross-Coupling Reactions Using Mechanochemistry. ACS Sustain. Chem. Eng..

[cit40] Seo T., Kubota K., Ito H. (2020). Selective Mechanochemical Monoarylation of Unbiased Dibromoarenes by *in Situ* Crystallization. J. Am. Chem. Soc..

[cit41] Takahashi R., Kubota K., Ito H. (2020). Air- and moisture-stable Xantphos-ligated palladium dialkyl complex as a precatalyst for cross-coupling reactions. Chem. Commun..

[cit42] Kubota K., Seo T., Koide K., Hasegawa Y., Ito H. (2019). Olefin-accelerated solid-state C–N cross-coupling reactions using mechanochemistry. Nat. Commun..

[cit43] Lennox C. B., Borchers T. H., Gonnet L., Barrett C. J., Koenig S. G., Nagapudi K., Friščić T. (2023). Direct mechanocatalysis by resonant acoustic mixing (RAM). Chem. Sci..

[cit44] Chantrain V., Rensch T., Pickhardt W., Grätz S., Borchardt L. (2024). Continuous Direct Mechanocatalytic Suzuki-Miyaura Coupling *via* Twin-Screw Extrusion. Chem. - Eur. J..

[cit45] Vogt C. G., Grätz S., Lukin S., Halasz I., Etter M., Evans J. D., Borchardt L. (2019). Direct Mechanocatalysis: Palladium as Milling Media and Catalyst in the Mechanochemical Suzuki Polymerization. Angew. Chem., Int. Ed..

[cit46] Floyd K., Gonnet L., Friščić T., Batteas J. (2024). The role of the milling environment on the copper-catalysed mechanochemical synthesis of Tolbutamide. RSC Mechanochem..

[cit47] Pickhardt W., Siegfried E., Fabig S., Rappen M. F., Etter M., Wohlgemuth M., Grätz S., Borchardt L. (2023). The Sonogashira Coupling on Palladium Milling Balls—A new Reaction Pathway in Mechanochemistry. Angew. Chem., Int. Ed..

[cit48] Yoo K., Fabig S., Grätz S., Borchardt L. (2023). The impact of the physical state and the reaction phase in the direct mechanocatalytic Suzuki–Miyaura coupling reaction. Faraday Discuss..

[cit49] Trinh Q. T., Golio N., Cheng Y., Cha H., Tai K. U., Ouyang L., Zhao J., Tran T. S., Nguyen T.-K., Zhang J. (2025). *et al.*, Sonochemistry and sonocatalysis: current progress, existing limitations, and future opportunities in green and sustainable chemistry. Green Chem..

[cit50] Amaniampong P. N., Trinh Q. T., De Oliveira Vigier K., Dao D. Q., Tran N. H., Wang Y., Sherburne M. P., Jérôme F. (2019). Synergistic Effect of High-Frequency Ultrasound with Cupric Oxide Catalyst Resulting in a Selectivity Switch in Glucose Oxidation under Argon. J. Am. Chem. Soc..

[cit51] Kant K., Devi T. A., Nitin, Malakar C. C. (2025). Role of Copper in Mechanochemical C–H Activation Approaches: Mechanochemistry in C–C and C–X Bonds Formation. Asian J. Org. Chem..

[cit52] Gonnet L., Borchers T. H., Lennox C. B., Vainauskas J., Teoh Y., Titi H. M., Barrett C. J., Koenig S. G., Nagapudi K., Friščić T. (2023). The “*η*-sweet-spot” (*η*max) in liquid-assisted mechanochemistry: polymorph control and the role of a liquid additive as either a catalyst or an inhibitor in resonant acoustic mixing (RAM). Faraday Discuss..

[cit53] Biesinger M. C. (2017). Advanced analysis of copper X-ray photoelectron spectra. Surf. Interface Anal..

[cit54] Aromaa J., Kekkonen M., Mousapour M., Jokilaakso A., Lundström M. (2021). The Oxidation of Copper in Air at Temperatures up to 100 °C. Corros. Mater. Degrad..

[cit55] Ansbacher T., Srivastava H. K., Martin J. M. L., Shurki A. (2010). Can DFT methods correctly and efficiently predict the coordination number of copper(I) complexes? A case study. J. Comput. Chem..

[cit56] Zhao G.-m., Liu H.-l., Zhang D.-d., Huang X.-r., Yang X. (2014). DFT Study on Mechanism of N-Alkylation of Amino Derivatives with Primary Alcohols Catalyzed by Copper(II) Acetate. ACS Catal..

[cit57] Straub B. F., Gruber I., Rominger F., Hofmann P. (2003). Mechanism of copper(I)-catalyzed cyclopropanation: a DFT study calibrated with copper(I) alkene complexes. J. Organomet. Chem..

[cit58] Fraile J. M., García J. I., Martínez-Merino V., Mayoral J. A., Salvatella L. (2001). Theoretical (DFT) Insights into the Mechanism of Copper-Catalyzed Cyclopropanation Reactions. Implications for Enantioselective Catalysis. J. Am. Chem. Soc..

[cit59] Isegawa M., Sameera W. M. C., Sharma A. K., Kitanosono T., Kato M., Kobayashi S., Morokuma K. (2017). Copper-Catalyzed Enantioselective Boron Conjugate Addition: DFT and AFIR Study on Different Selectivities of Cu(I) and Cu(II) Catalysts. ACS Catal..

[cit60] Wu L., Sheong F. K., Lin Z. (2020). DFT Studies on Copper-Catalyzed Dearomatization of Pyridine. ACS Catal..

[cit61] Pladevall B. S., de Aguirre A., Maseras F. (2021). Understanding Ball Milling Mechanochemical Processes with DFT Calculations and Microkinetic Modeling. ChemSusChem.

[cit62] Peter R., Petravic M. (2021). Initial Stages of Oxide Formation on Copper Surfaces during Oxygen Bombardment at Room Temperature. J. Phys. Chem. C.

[cit63] Castrejón-Sánchez V.-H., Solís A. C., López R., Encarnación-Gomez C., Morales F. M., Vargas O. S., Mastache-Mastache J. E., Sánchez G. V. (2019). Thermal oxidation of copper over a broad temperature range: towards the formation of cupric oxide (CuO). Mater. Res. Express.

[cit64] Bolt R. R. A., Raby-Buck S. E., Ingram K., Leitch J. A., Browne D. L. (2022). Temperature-Controlled Mechanochemistry for the Nickel-Catalyzed Suzuki–Miyaura-Type Coupling of Aryl Sulfamates *via* Ball Milling and Twin-Screw Extrusion. Angew. Chem., Int. Ed..

[cit65] Michalchuk A. A. L., Tumanov I. A., Boldyreva E. V. (2013). Complexities of mechanochemistry: elucidation of processes occurring in mechanical activators *via* implementation of a simple organic system. CrystEngComm.

[cit66] Rappen M. F., Beissel L., Geisler J., Tietmeyer S. T., Grätz S., Borchardt L. (2024). Polymer vessels in mechanochemical syntheses: assessing material performance. RSC Mechanochem..

[cit67] Puccetti F., Schumacher C., Wotruba H., Hernández J. G., Bolm C. (2020). The Use of Copper and Vanadium Mineral Ores in Catalyzed Mechanochemical Carbon–Carbon Bond Formations. ACS Sustain. Chem. Eng..

[cit68] Sheibani S., Ataie A., Heshmati-Manesh S., Khayati G. R. (2008). Processing of nanocrystalline copper by mechanochemical reduction of CuO and Cu2O with graphite. Mater. Sci. Technol..

[cit69] Lucchesi Schio A., Farias Soares M. R., Machado G., Barcellos T. (2021). Improved Mechanochemical Fabrication of Copper(II) Oxide Nanoparticles with Low E-Factor. Efficient Catalytic Activity for Nitroarene Reduction in Aqueous Medium. ACS Sustain. Chem. Eng..

[cit70] Morozova O., Firsova A., Tyulenin Y., Vorobieva G., Leonov A. (2020). Mechanochemical Synthesis as an Alternative Effective Technique for the Preparation of the Composite Catalysts. Kinet. Catal..

[cit71] Khayati G. R., Nourafkan E., Karimi G., Moradgholi J. (2013). Synthesis of cuprous oxide nanoparticles by mechanochemical oxidation of copper in high planetary energy ball mill. Adv. Powder Technol..

[cit72] Tian Z., Wang S., Li R., Sun X., Shen W., Liu S. (2025). Study on polishing mechanisms of BEOL metal interconnects based on chemical and mechanical synergy. Microsyst. Nanoeng..

[cit73] Platzman I., Brener R., Haick H., Tannenbaum R. (2008). Oxidation of Polycrystalline Copper Thin Films at Ambient Conditions. J. Phys. Chem. C.

[cit74] Wang J., Li C., Zhu Y., Boscoboinik J. A., Zhou G. (2018). Insight into the Phase Transformation Pathways of Copper Oxidation: From Oxygen Chemisorption on the Clean Surface to Multilayer Bulk Oxide Growth. J. Phys. Chem. C.

[cit75] Andersson K., Ketteler G., Bluhm H., Yamamoto S., Ogasawara H., Pettersson L. G. M., Salmeron M., Nilsson A. (2008). Autocatalytic Water Dissociation on Cu(110) at Near Ambient Conditions. J. Am. Chem. Soc..

[cit76] Thiel P. A., Madey T. E. (1987). The interaction of water with solid surfaces: Fundamental aspects. Surf. Sci. Rep..

[cit77] Fan Y., Li R., Wang B., Feng X., Du X., Liu C., Wang F., Liu C., Dong C., Ning Y. (2024). *et al.*, Water-assisted oxidative redispersion of Cu particles through formation of Cu hydroxide at room temperature. Nat. Commun..

[cit78] Mistry K., Snowden H., Darling G. R., Hodgson A. (2024). Hydroxyl on Stepped Copper and its Interaction with Water. J. Phys. Chem. C.

[cit79] Luo D., Wang X., Li B.-W., Zhu C., Huang M., Qiu L., Wang M., Jin S., Kim M., Ding F. (2021). *et al.*, The Wet-Oxidation of a Cu(111) Foil Coated by Single Crystal Graphene. Adv. Mater..

[cit80] Oda S., Masui Y., Omura S., Imamura Y., Takeuchi Y., Hosoya M. (2022). Addition Reaction of Alcohol to Isocyanate Catalyzed by Copper Present in Tap Water: Robust Manufacturing Process of Naldemedine Tosylate. Org. Process Res. Dev..

[cit81] Floyd M. D., Ryan L. Y., Hendsey J. L., Nicholson J. M., Palaia A. T., Isaacs A. K. (2022). Copper-catalyzed three-component synthesis of pyrrole-substituted 1,2-dihydroisoquinolines. Synth. Commun..

