# Supplementary material for: Harnessing the copper surface for direct mechanocatalysis: a case study on mechanochemical sulfonylurea synthesis
Source: Chem Sci. 2025 Sep 9;16(42):19830–42. doi: 10.1039/d5sc04099j (PMC12464735; doi:10.1039/d5sc04099j)
Supplement: SC-016-D5SC04099J-s001 [file SC-016-D5SC04099J-s001.pdf]

Supplementary Information (SI) for Chemical Science.
This journal is © The Royal Society of Chemistry 2025

SUPPORTING INFORMATION

The role of the copper surface in mechanocatalysis: A case study on mechanochemical sulfonylurea synthesis

Kathleen R. Floyd,[a] Lizette S. Mella,[a] Ryan Kwok,[b,c] Mackenzie Gray,[a] Edward J. Broker, Jr.,[a] Mateusz Marianski,[b,c] Tomislav Friščić[d] and James D. Batteas*[a,e]

a. Department of Chemistry, Texas A&M University, College Station, TX 77842-3012, USA. E-mail: batteas@chem.tamu.edu
b. Department of Chemistry, Hunter College, New York, NY 10065, USA. E-mail: mateusz.marianski97@login.cuny.edu
c. Ph.D. Program in Chemistry, Graduate Center of the City University of New York, New York, NY 10016, USA.
d. School of Chemistry, University of Birmingham, Edgbaston, Birmingham B152TT, United Kingdom. E-mail: t.friscic@bham.ac.uk
e. Department of Materials Science and Engineering, Texas A&M University, College Station, TX 77842-3012, USA.

Table of Contents

1. CHEMICALS AND HAZARDS 2
2. MATERIALS AND METHODS 3
2.1 NexION 300D Operating Parameters 3
2.2 Atmospherically Sealed Milling Jars 4
2.3 Inserts for Segmenting Jar Materials Into Different Regions 4
2.4 Raman Spectroscopy 6
2.5 FTIR Spectroscopy 6
2.6 Power X-ray Diffraction (PXRD) 6
3. ADDITIONAL PROCEDURES FOR THE TOLBUTAMIDE SYNTHESIS 6
3.1 Tolbutamide Purification Workup 6
3.2 A Note on Yield Determination by NMR 6
3.3 A Note on Reaction Aging in the Solid State 6
4. SUMMARY OF ^{1}H, ^{13}C AND HRMS DATA 7
5. XPS OF UNTREATED CU BB SURFACES 8
6. EFFECT OF CU CURVATURE ON CU BB XPS 11
7. XPS ANALYSIS OF COPPER SURFACE CLEANING TREATMENTS 14
7.1 Cleaned Copper Surfaces 14
7.2 Cleaned Copper Surface After 10 Days of Ambient Atmosphere Exposure 20
7.3 Determination of Cleaning Process 24
8. XPS DATA TO DETERMINE IDEAL UV OZONE OXIDATION TIME 24
9. ACETONE CLEANING FOR POST MILLING SURFACE ANALYSIS 27

10. O 1S XPS PEAK FITTING AS A FUNCTION OF MILLING TIME 28
11. STUDIES ON COPPER WEAR FROM ISOLATED REGIONS OF THE JAR 28
12. KINETIC STUDIES OF CU WEAR 29
13. ATTEMPTING TO ISOLATE $CU(OH)_2$ FORMATION IN THE POWDER 30
13.1 Raman Spectroscopy 30
13.2 FTIR Spectroscopy 33
13.3 Powder X-ray Diffraction (PXRD) 35
13.4 XPS 36
14. DFT CALCULATIONS OF THE REACTION USING $CACL_2$ AND $ZNCL_2$ 37
15. DOI LINK TO RAW DATA FILES 38
16. REFERENCES 38

1. CHEMICALS AND HAZARDS

Reagents are commercially available. For each substance, hazards are indicated according to Regulation (EC) No 1272/2008.

p-toulenesulfonamide: CAS [70-55-3], 98+% Alfa Aesar – non-hazardous

n-butyl isocyanate: CAS [111-36-4], 98% Sigma Aldrich – H225, H302, H311, H314, H330, H334

- n-butyl isocyanate is a flammable lachrymator liquid causing acute toxicity if inhaled, swallowed, or adsorbed through skin or eyes. It is not considered by the IARC (International Agency for Research on Cancer) to persist or bioaccumulate at levels of 0.1% or higher.

Ethylenediaminetetraacetic acid disodium salt dihydrate: CAS [6381-92-6], 99-100% Sigma-Aldrich – H332, H373

- Ethylenediaminetetraacetic acid disodium salt dihydrate is harmful if inhaled and may cause damage to the respiratory tract through prolonged repeated exposure.

Tolbutamide: CAS [64-77-7] – H303.

- Tolbutamide is not defined as a hazardous substance according to Regulation (EC) No 1272/2008 with [LD50 Oral – Rat: 2.490 mg/kg]

Dimethyl sulfoxide-d_6: CAS [2206-27-1] – H227

Phosphorus pentoxide: CAS [1314-56-3] – H314, P280, P310, P351, P338, P310, EU014

Ethanol: CAS [64-17-5] 200 proof, Koptec – H225, P210, P233, P240, P241, P242, P243, P280, P303, P361, P353, P370, P378, P403, P235, and P501

Citric Acid : CAS [77-92-9], Mrs.Wages® Citric Acid, Kent Precision Foods Group < 2% silicon dioxide (anticaking) – Non-hazardous

Copper (II) oxide: CAS [1317-38-0], Thermo Scientific 99.7% – H400, H412, P273

- Harmful if swallowed, toxic to aquatic life

Copper (I) oxide: CAS [1317-39-1] Strem Chemicals Inc. 95% – H302, P264, P270, P301, P312, P330, P50114)

- Eye and respiratory tract irritant

Copper (II) hydroxide: CAS [20427-59-2] BeanTown Chemical 94% – H302, H318, H319, H330, H400, H410, H411, P260, P264, P264+P265, P270, P271, P273, P280, P284, P301+P317, P304+P340, P305+P351+P338, P305+P354+P338, P316, P317, P320, P330, P337+P317, P391, P403+P233, P405, and P501

- Acute oral and inhalation toxicity, can cause serious eye irritation and damage on contact, very toxic to aquatic life

Dry Air: 99%, < 3ppm moisture – Nonhazardous

Dry Nitrogen: – Nonhazardous

2. MATERIALS AND METHODS

2.1 NexION 300D Operating Parameters

Table S1. ICP-MS additional instrument operating parameters.

**NexION 300D Operating Parameters**	
RF Power	1600 W
Plasma Ar Flow	18.0 L min^{-1}
Auxiliary Ar Flow	1.20 L min^{-1}
Nebulizer Ar Flow	0.98 L min^{-1}
Sample Introduction System	Concentric nebulizer with cyclonic spray chamber
Operating Frequency	40 MHz
Sample Uptake Rate	1 mL min^{-1}
Detector Mode	Analog
Sampler/Skimmer/Hyper-skimmer Cones	Ni/Ni/Al
Scanning Mode	Peak hopping
Number of points per peak	5
Dwell Time	50 ms
Sweeps per Reading	20
Isotopes	^{52}Cr, ^{57}Fe, ^{63}Cu
Internal Standards	^{45}Sc
Software	Syngistix Software, Version 3.4

2.2 Atmospherically Sealed Milling Jars

Previous attempts to use parafilm on SS with tight screwing of traditional 25 mL Retsch MM400 jars were unsuccessful at sealing out atmosphere reliably as threading was observed to loosen overtime. As such, specialized 25 mL jars capable of sealing out atmosphere via a KF-25 flange vacuum joint were manufactured out of 316 stainless steel (see Figures S1 and S2). Initially, KF wing nut clamps were explored for use with the designed jars. However, KF wing nut clamps unwound during the course of milling as with the previously threaded milling jars. A KF-25 toggle clamp and locking pin will solve this issue and endure well for prolonged milling. SolidWorks files are available upon request for readers interested in manufacturing similar jars.

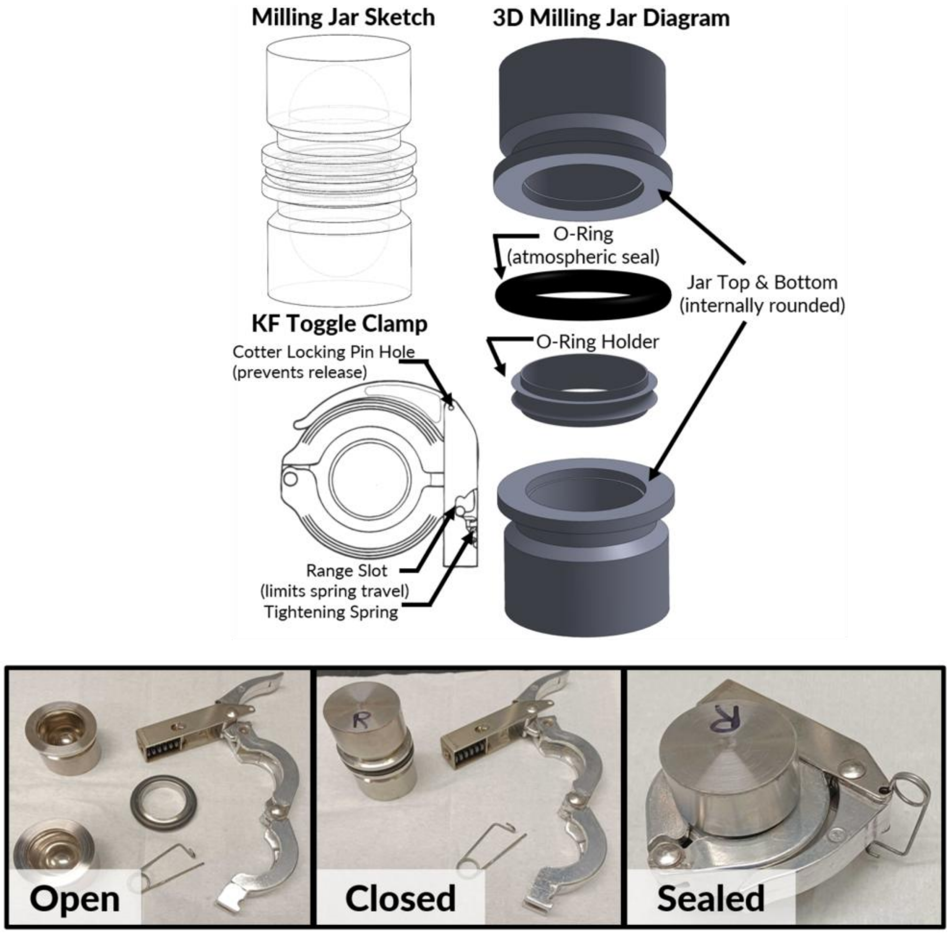

Figure S1. Atmospherically sealed jars sketch, diagram, and images of disassembled and assembled parts.

2.3 Inserts for Segmenting Jar Materials Into Different Regions

Inserts made to fit within the 25 mL hermetically sealed jar or a traditional Retsch MM 400 jar were made from copper (McMaster Carr, Super-Conductive 101) and stainless steel (McMaster Carr, 316 SS). Inserts are composed of two end caps and an internal middle cylinder. These

pieces fit together so different materials can be isolated to either the middle of the jar or ends of the jar for exploring different behavior from each region. Inserts fit snuggly within outer casing such that they are immobile when the jars are closed. See Figure S2 for the dimensions of the inserts and in-house hermetically sealed jars (note internal dimensions are identical to Retsch MM 400 25 mL jars). See Figure S3 for a close-up view of the inserts and the typically associated forces experienced in different milling regions. We note that while impact/compression dominates at the jar ends and shear dominates in the middle portion of the jars, some impact/compression is still likely to occur in the middle of the jar due to the chaotic nature of ball movement.

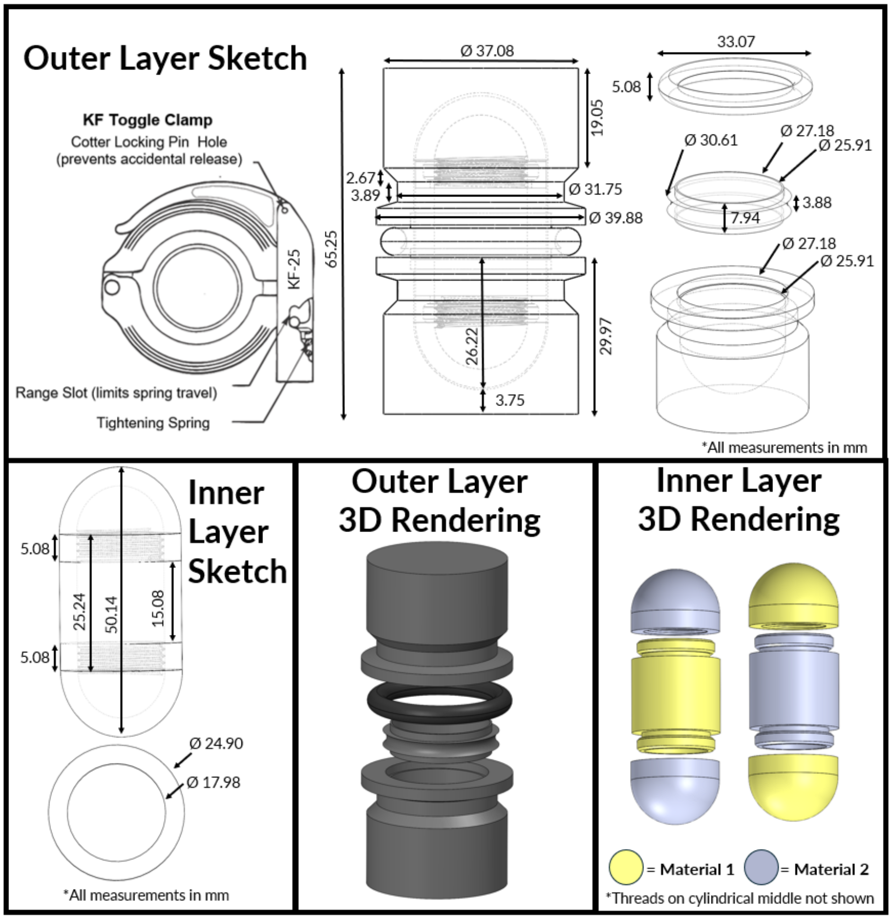

Figure S2. Atmospherically sealed ball milling jars and inserts sketch and 3D diagrams.

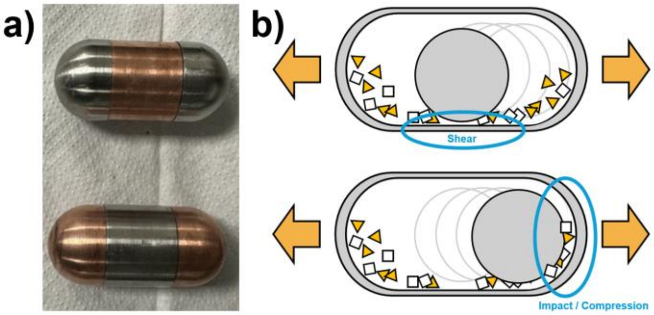

Figure S3. Inserts used for wear studies showing a) closed inserts and b) regions of milling jar and associated most frequent type of force.

2.4 Raman Spectroscopy

Raman spectra were collected using a WITec Alpha 300RA confocal microscope equipped with a 100× objective (Nikon E Plan, 0.9 NA), 488 nm diode laser, a UHTS 300 VIS spectrometer, and a cooled (−60 °C) Andor EMCCD detector. These point spectra are an average of 10 individual spectra collected with 10 s or 20 s integration time, using a 1800 g mm^{-1} grating and a laser power of ~1 mW for copper oxide species and ~60 μW for tolbutamide and crude reaction powder. The use of lower laser power for the organic matrix was necessary to prevent sample degradation.

2.5 FTIR Spectroscopy

FTIR spectra were collected in transmission mode using a Nicolet 6700 FTIR spectrometer operated using OMNIC software and equipped with a liquid nitrogen cooled Mercury Cadmium Telluride (MCT)-A detector and KBr beam splitter. All spectra were taken using 100 scans and a spectral resolution of 1 cm^{-1} and using a KBr pellet as background. Prior to analysis, samples were ground with dried KBr powder (~7 mg sample in 100 mg KBr) and were pressed into pellets (50 mg) using Specac press with a 12 ton pressure applied three times, rotating the sample a third of the way each time.

2.6 Power X-ray Diffraction (PXRD)

XRD patterns for powder samples were collected at room temperature and ambient atmosphere using a Bruker-AXS D8 Endeavor X-ray Powder Diffractometer (D8 Goniometer, LynxEye PSD XTE detector (detector opening=1.006° or 4°), 0.2 or 0.6 mm fixed divergence slit, Cu-Kα radiation). Patterns were collected for a two-theta range of 5 to 60 or 90° with 0.020 step size and 1 or 0.70 s per step.

3. ADDITIONAL PROCEDURES FOR THE TOLBUTAMIDE SYNTHESIS

3.1 Tolbutamide Purification Workup

A total of 7.5 mL of water and $Na_2H_2EDTA \cdot 2H_2O$ (50 mg, 0.17 mol) were added to the remaining crude reaction mixture and milled for 10 min. The product was separated by vacuum filtration, rinsed using 80 ml of nanopure H_2O (18.2 MΩ · cm, Barnstead) and dried in an Accutemp AT09 vacuum oven at 700 mmHg over P_4O_5 at 25°C overnight. Purified product was dissolved in d_6-dimethylsulfoxide. Sample was pipetted through a filter pipette and ^1H NMR of the purified product was taken.

3.2 A Note on Yield Determination by NMR

As described in previous studies, when *p*-toluenesulfonamide (214 mg, 1.25 mmol), *n*-butyl isocyanate (140.8 μL, 1.25 mmol), and copper in a 2+ oxidation state are added directly to d_6-DMSO, the reaction can proceed noticeably after mixing within the NMR tube over the course of a few hours.[1] In our reactions, NMR was obtained immediately following the dissolution of crude product to ensure data is representative solely of the mechanochemical phenomenon at play and not further reaction within the NMR tube.

3.3 A Note on Reaction Aging in the Solid State

It was found that reactions performed mechanocatalytically could age on Cu surfaces or with sufficient worn Cu present in the powder matrix if the reaction mixture remained "wet" (chiefly from unreacted *n*-butyl isocyanate) upon removal from the milling jars (see Figure S4 and Table

S2). Aging is generally accompanied by the powder turning green. This result is contrary to reactions previously studied with $CuCl_2$ as catalyst and associated control experiments verifying aging in powder with reaction quantities did not occur. Further investigations into this phenomenon are underway. To ensure that yields obtained were the result of the mechanochemical milling rather than aging, ^{1}H NMR measurements were taken immediately upon reaction completion excepting some trials that ran before the discovery of the aging phenomenon where yields were observed to be 0% regardless of time sitting in the powder.

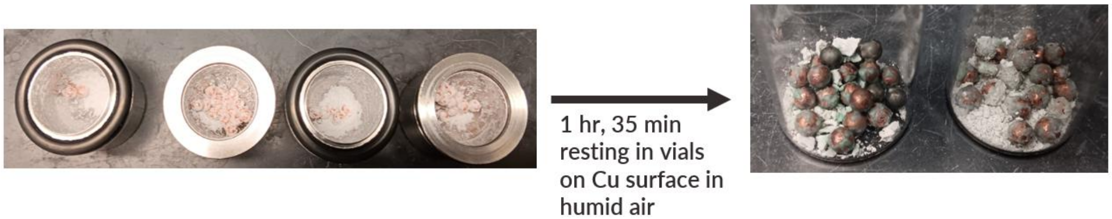

Figure S4. Reaction aging on copper surface. Product that appears green has higher associated yields while white powder remains unreacted after milling.

Table S2. Yields of milled powder after reaction under inert atmosphere after 1 hr and 35 minutes of aging on a Cu surface under ambient atmosphere examining both green solid formed after leaving the mixture on the Cu surface and white solid in remaining jar physically removed far from the Cu.

Milling Atmosphere	Cu BB Surface Oxidation	White Aged Product Yield (%)	Green Aged Product Yield (%)
N_2	Cu (I+0)	0	26.5
N_2	Cu (II)	0	18.0

4. SUMMARY OF ^{1}H, ^{13}C AND HRMS DATA

N-butylcarbamoyl)-4-methylbenzenesulfonamide CAS [64-77-7] – Tolbutamide White powder, m.p. 125.2-126.4 °C.[2] **^{1}HNMR** (400 MHz, DMSO-d_6) δ (ppm): 10.45 (s_{broad}, 1H, NHS), 7.78 (d, J =8.0 Hz, 2H, CH_{Ar}), 7.40 (d, J =8.0 Hz, 2H, CH_{Ar}), 6.42 (m, 1H, NHC=O), 2.94 (t, J = 6.4 Hz, 2H, CH_2), 2.39 (s, 3H, CH_3), 1.33-1.26 (m, 2H, CH_2), 1.19-1.14 (m, 2H, CH_2), 0.82 (t, J = 7.2 Hz, 3H, CH_3) **^{13}C NMR** (500 MHz, DMSO-d_6) δ (ppm): 151.7, 143.3, 129.3, 127.1, 39.5, 31.3, 30.6, 21.0, 19.2, 13.5 **HRMS ESI (+):** calculated for $C_{12}H_{19}O_3N_2S$ $[M + H]^+$: 271.1111; measured: 271.1104

5. XPS OF UNTREATED CU BB SURFACES

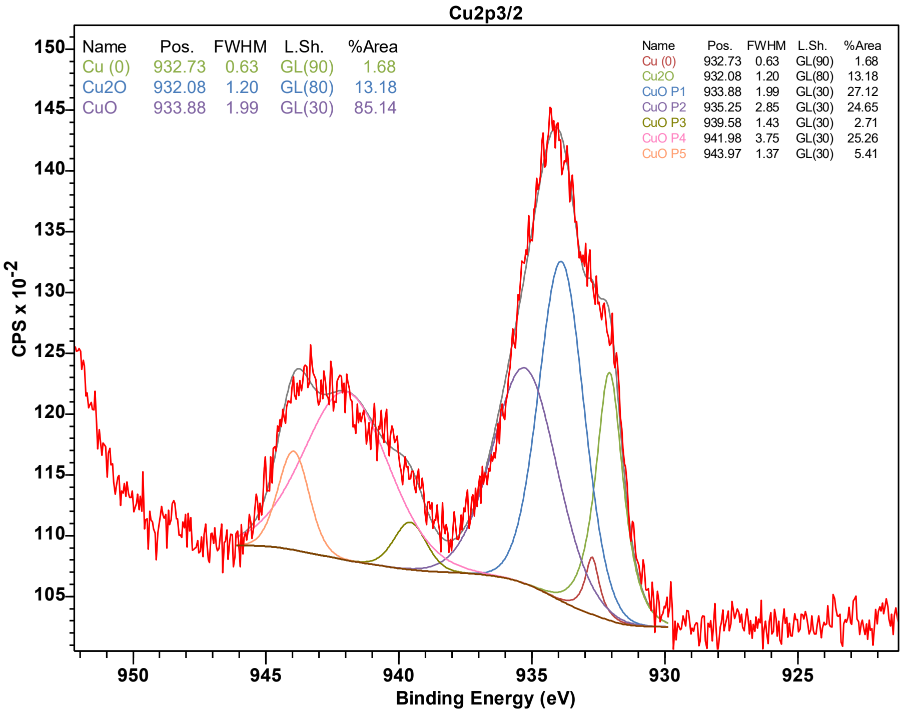

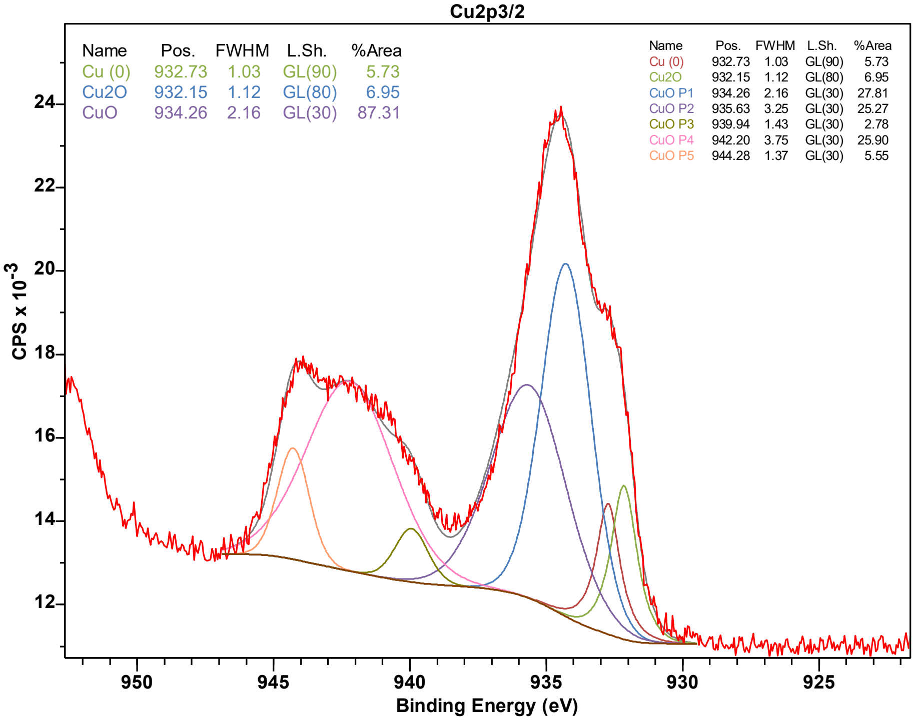

Figure S5. XPS data of untreated Cu BBs Cu2p$^{3/2}$ peak fit to traditional peaks of Cu and associated oxides. Peak fits are shown along with peak positions, FWHM, line shapes, and % Area.

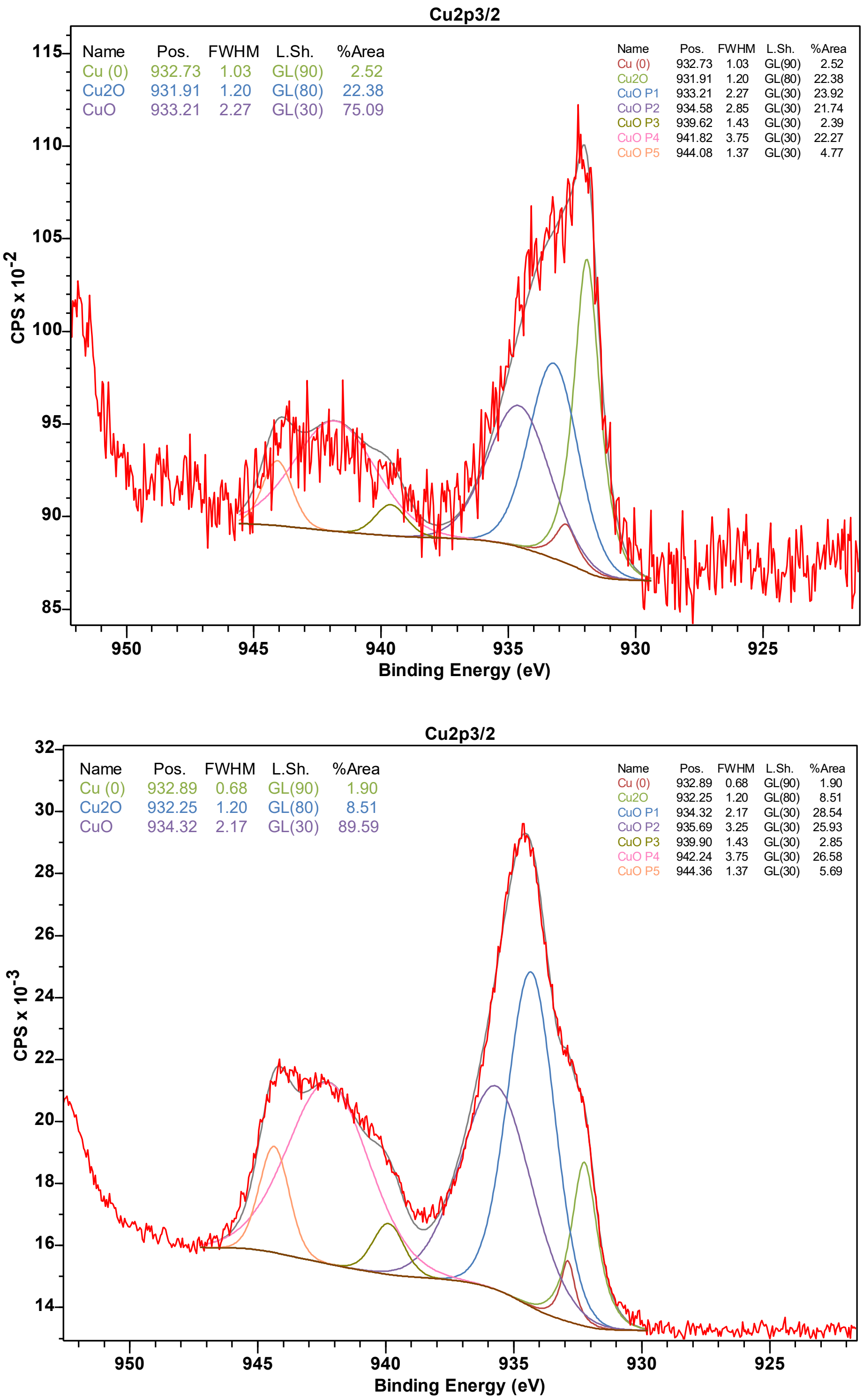

Figure S6. XPS data of untreated Cu BBs Cu2p$^{3/2}$ peak fit to traditional peaks of Cu and associated oxides. Peak fits are shown along with peak positions, FWHM, line shapes, and % Area.

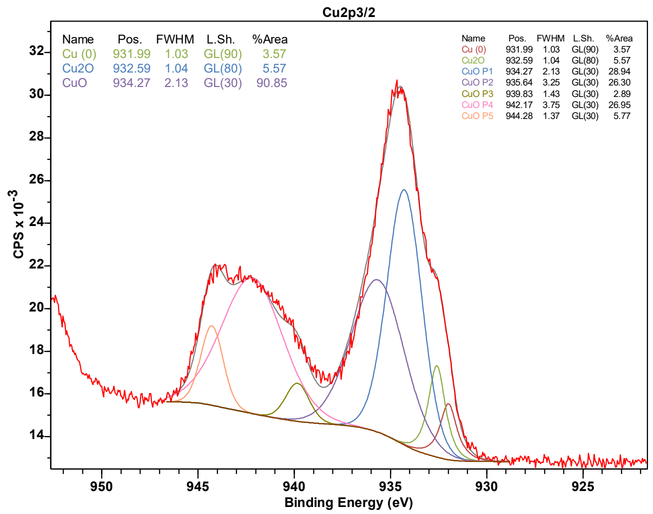

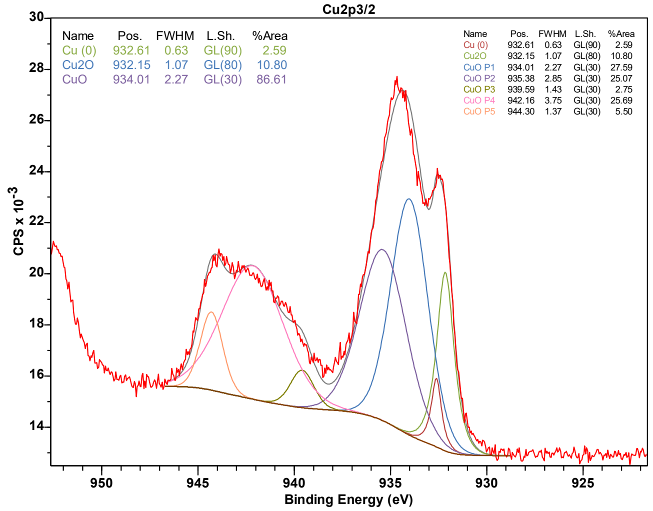

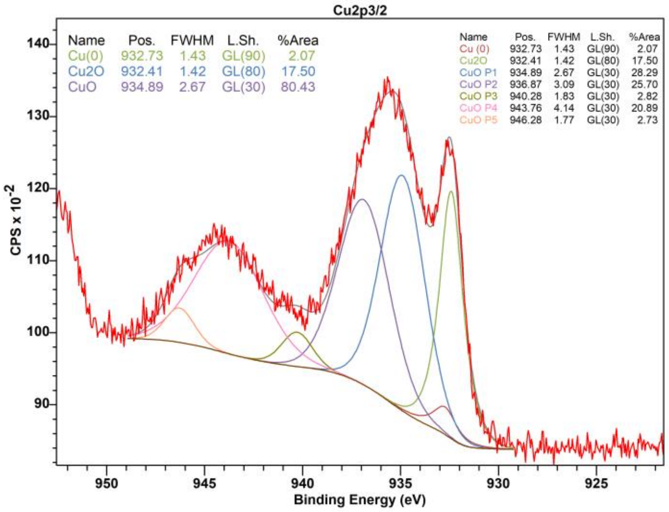

Figure S7. XPS data of untreated Cu BBs Cu2p$^{3/2}$ peak fit to traditional peaks of Cu and associated oxides. Peak fits are shown along with peak positions, FWHM, line shapes, and % Area.

Readers should note that the Cu (0) and Cu_2O peaks in the Cu $2p^{3/2}$ spectra overlap and judgement between the amounts of each should only be done based on Cu LMM data which can show qualitatively (fitting is too complex to be relied on rigorously in a quantitative sense) which makes up a majority of the peak. Thus, the extent of Cu (0) vs Cu_2O in Figures S5-S7 should not be inferred from these figures. Furthermore, shake-up/satellite peaks in the Cu $2p^{3/2}$ spectra are treated differently in literature with some authors choosing to neglect the peak area of shake up peaks in quantification to simplify fitting and others including the area for quantification. Most of the shake-up/satellite peaks in Figures S5-S7 qualitatively appear like those of CuO peaks though some samples have a shape resembling $Cu(OH)_2$ (see Figure S7, bottom and the native copper rod in the Figure S11 for reference). Further fitting information for Cu species is available from Besinger et al and at https://www.xpsfitting.com/2012/01/copper.html.[3]

6. EFFECT OF CU CURVATURE ON CU BB XPS

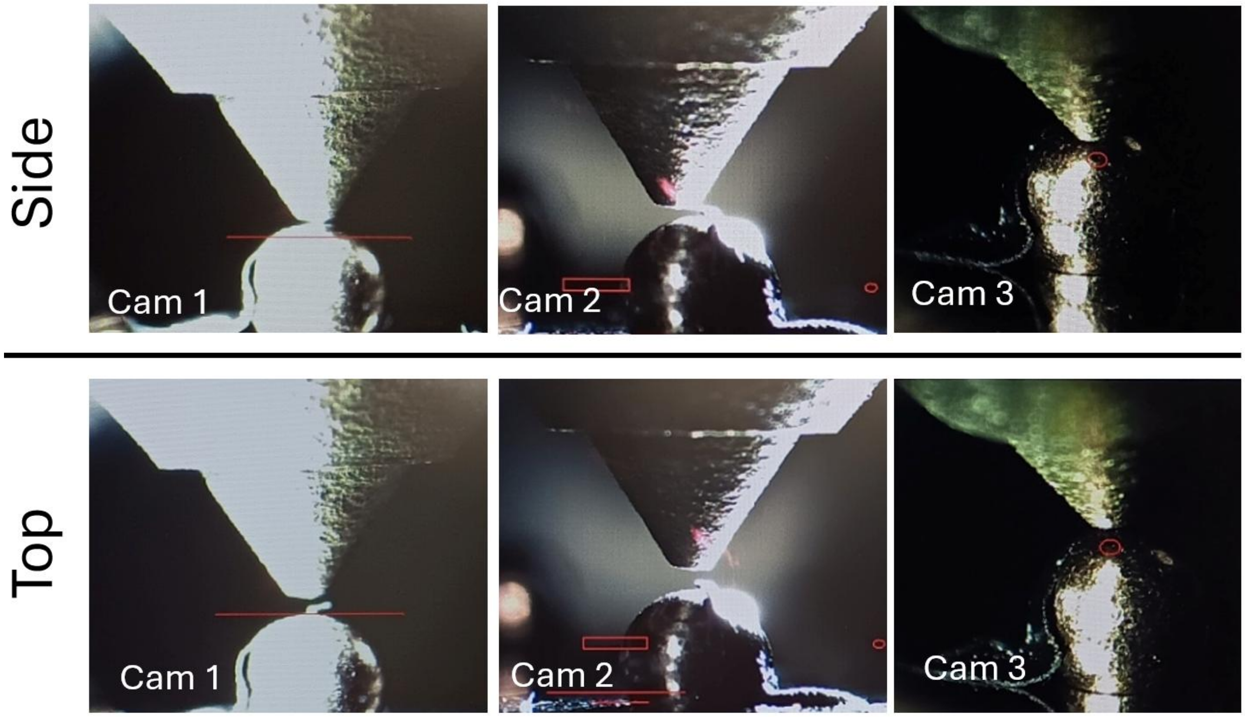

Figure S8. Images from sample centering cameras showing beam probe when set at the top of the ball as close to directly over as possible (top) and when set at the furthest possible curvature value where scattering beam could be collected by the detector and data could still be obtained (side).

Traditionally XPS is performed on somewhat "flat" surfaces. In theory, if the curvature of the surface is more extensive, an offset beam could pass through more of the surface than the bulk artificially boosting signal from a thin oxide on the surface compared to underlying bulk signal. While we attempted to keep the beam as directly on the top of the Cu BBs as possible, the effect of curvature on quantification was explored as a control (see Figure S8). Measured differences between Cu (0+I) species and Cu (II) species were within 5% of one another with all peak shapes correspondingly similar with similar fits suggesting there were no statistically significant differences arising from probe placement above the Cu BB (see Figure S9) . This result was consistent regardless of initial surface oxidation (see Figure S10).

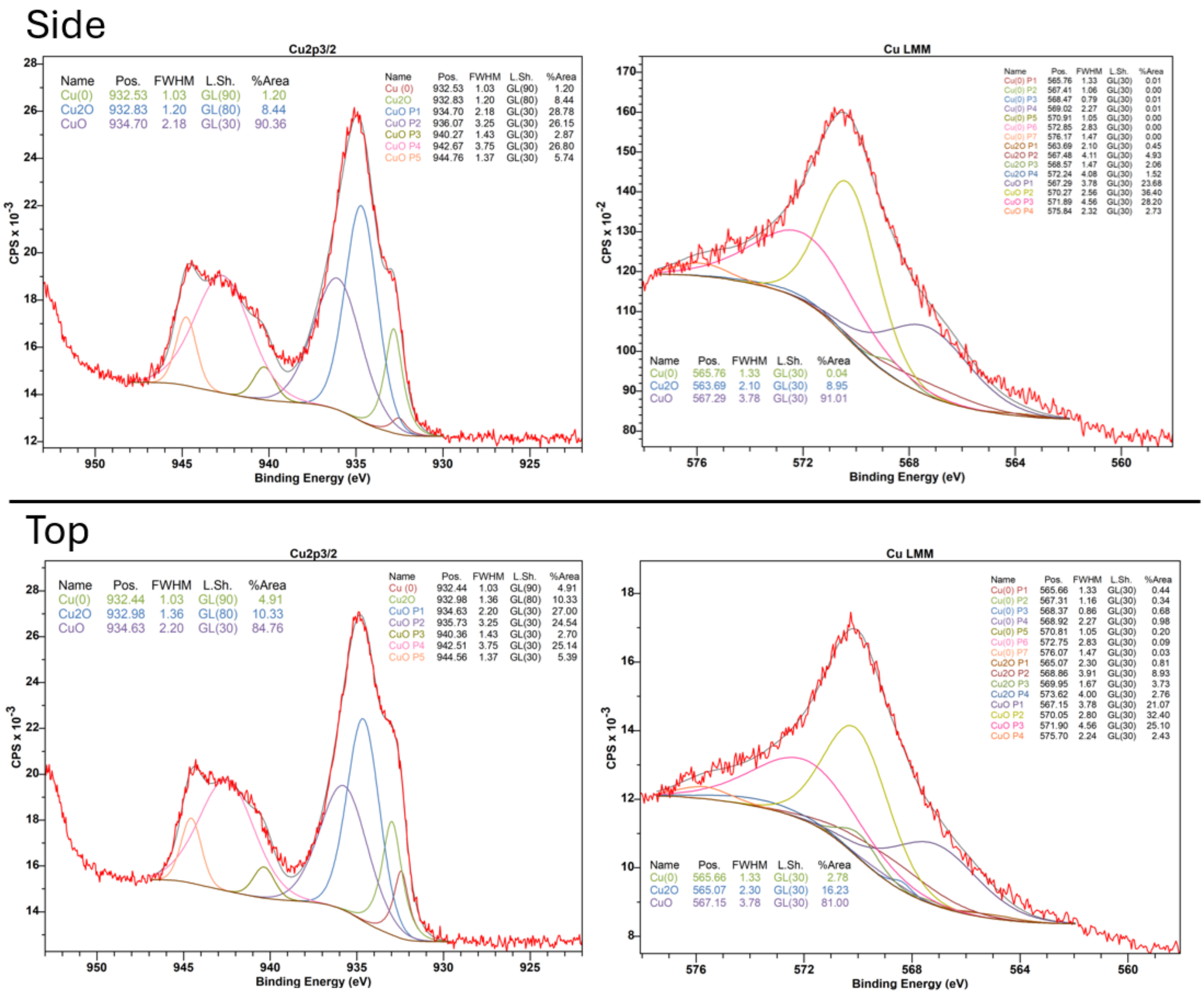

Figure S9. Cu 2p$^{3/2}$ and Cu LMM XPS data obtained on the top and side of an oxidized Cu BB showing nearly identical spectra and fitting. Peak fits are shown along with peak positions, FWHM, line shapes, and % Area.

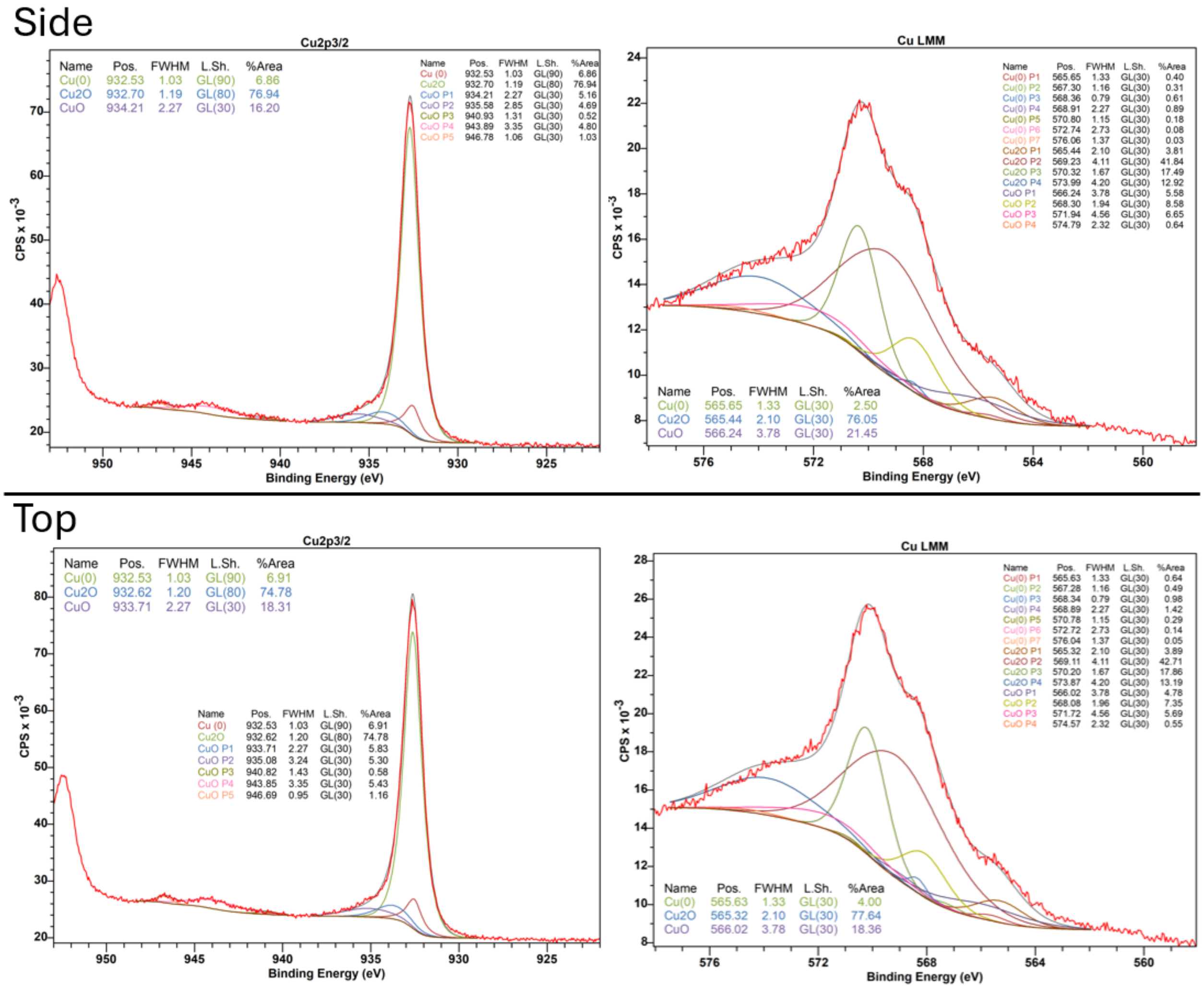

Figure S10. Cu $2p^{3/2}$ and Cu LMM XPS data obtained on the top and side of a cleaned Cu BB showing nearly identical spectra and fitting. Peak fits are shown along with peak positions, FWHM, line shapes, and % Area.

7. XPS ANALYSIS OF COPPER SURFACE CLEANING TREATMENTS

7.1 Cleaned Copper Surfaces

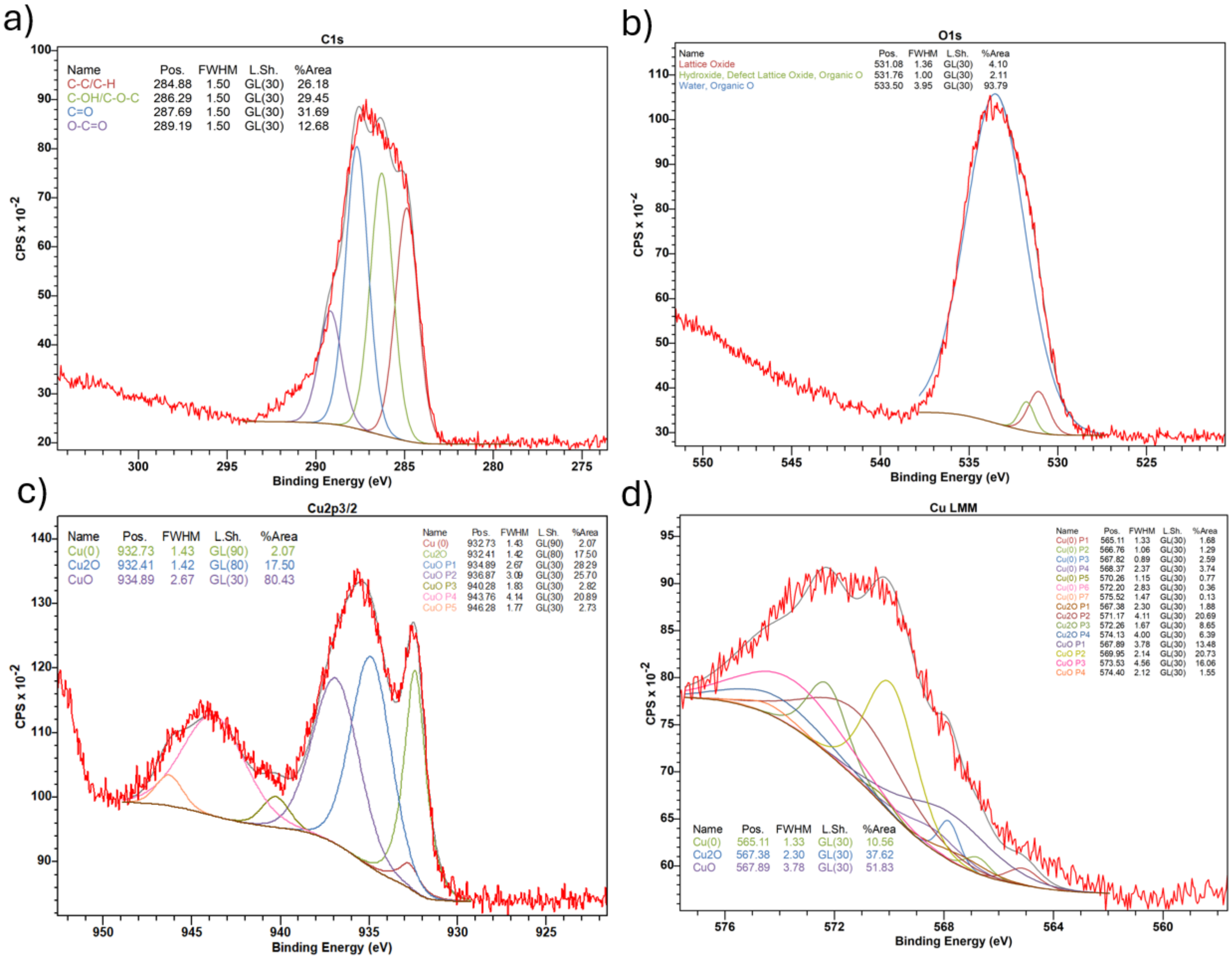

Figure S11. XPS of pure Cu rods before cleaning showing spectra of a) C 1s, b) O 1s, c) Cu $2p^{3/2}$, and d) Cu LMM. Peak fits are shown along with peak positions, FWHM, line shapes, and % Area.

Copper rods for cleaning treatments were purchased from McMaster Carr (Super-Conductive 101, 1/8" Diameter). Initial rod surface was prepared by rinsing in nanopure H_2O (18.2 MΩ · cm, Barnstead), rinsing under EtOH, and drying under streaming N_2 (g) followed by storage under inert atmosphere until examination by XPS following established XPS acquisition methodology for Cu BBs. The surface was shown to be 80.43% CuO according to fitting of the Cu $2p^{3/2}$ spectrum suggesting it was significantly oxidized (see Figure S11). These results were in agreement with the results of the C 1s, O 1s, and Cu LMM findings. As previously, readers should note that the Cu (0) and Cu_2O peaks in the Cu $2p^{3/2}$ spectra overlap and judgement between the amounts of each should only be done based on Cu LMM data which can show qualitatively (fitting is too complex to be relied on rigorously in a quantitative sense) which makes up a majority of the peak. Furthermore, shake-up peaks in the Cu $2p^{3/2}$ spectra are treated differently in literature with some authors choosing to neglect the peak area of shake up peaks in quantification to simplify fitting and others including the area for quantification.[3] We have chosen to fit the shake up features. Further fitting information for Cu species is available from Besinger et al and at https://www.xpsfitting.com/2012/01/copper.html.[3]

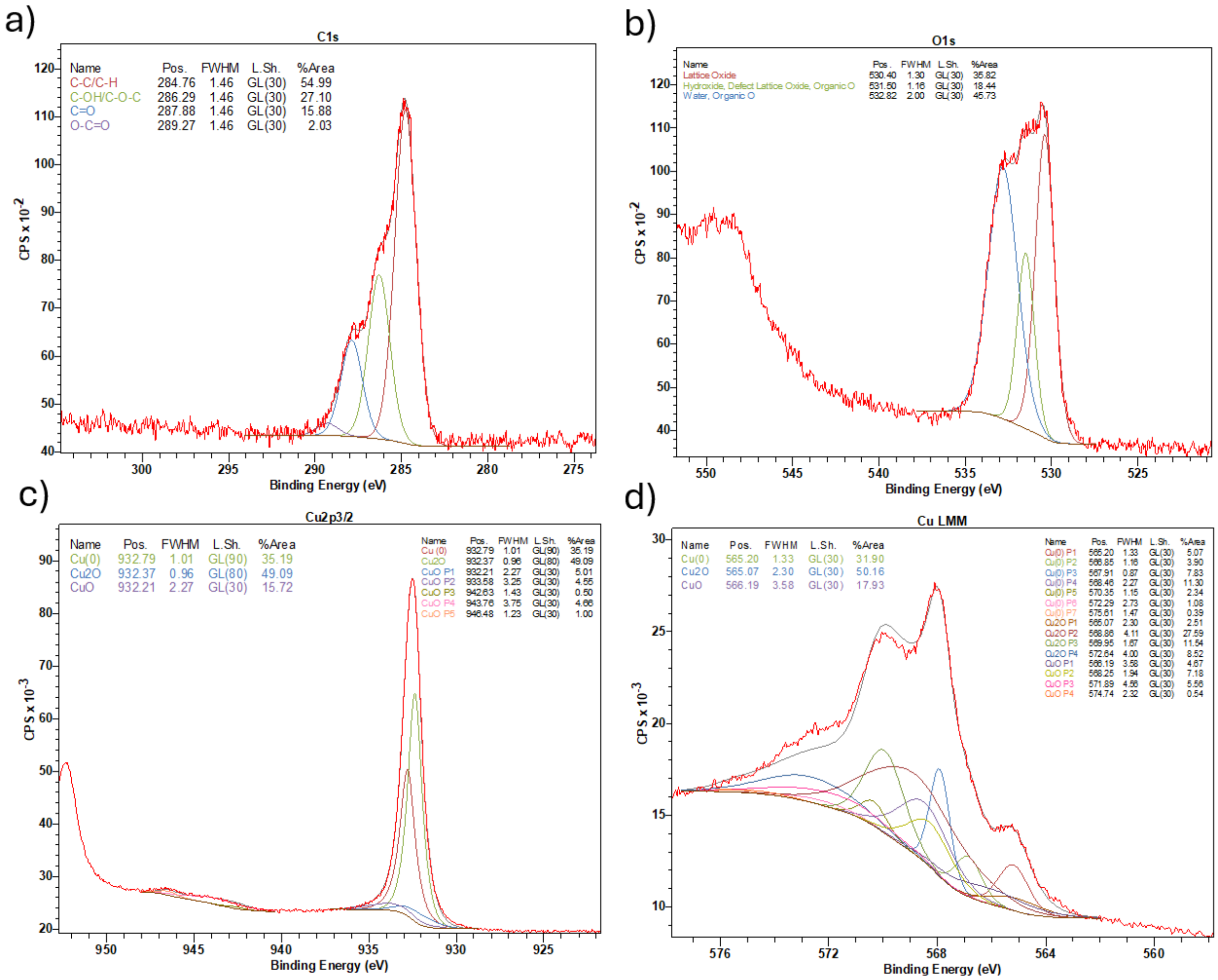

Figure S12. XPS of Cu rods cleaned using the lemon-cornstarch method showing spectra of a) C 1s, b) O 1s, c) Cu $2p^{3/2}$, and d) Cu LMM. Peak fits are shown along with peak positions, FWHM, line shapes, and % Area.

To clean the surface of the Cu, a number of techniques were examined. The lemon-cornstarch cleaning method was as follows: initial rod surface was prepared by rinsing in nanopure H_2O (18.2 MΩ · cm, Barnstead), rinsing under EtOH, and drying under streaming N_2 (g). Subsequently, cornstarch (1.5 g) was mixed with NaCl (1.5 g) and dissolved in 2 mL of lemon juice to make a slightly abrasive paste. The paste was rubbed over the Cu surface using a new clean microfiber cloth. Subsequently, the rod was rinsed in nanopure H_2O (18.2 MΩ · cm, Barnstead), rinsed under EtOH, dried under streaming N_2 (g), and stored under inert atmosphere until examination by XPS following established XPS acquisition methodology for Cu BBs. XPS Cu $2p^{3/2}$ results showed CuO oxidation was reduced to 15.7 % area coverage with Cu_2O and Cu (0) dominating the remainder of the surface. Cu LMM reveals Cu_2O was more abundant that Cu (0). Both the C 1s and O 1s spectra indicate that organic contamination on the Cu surface reduced significantly compared to the pure Cu rod.

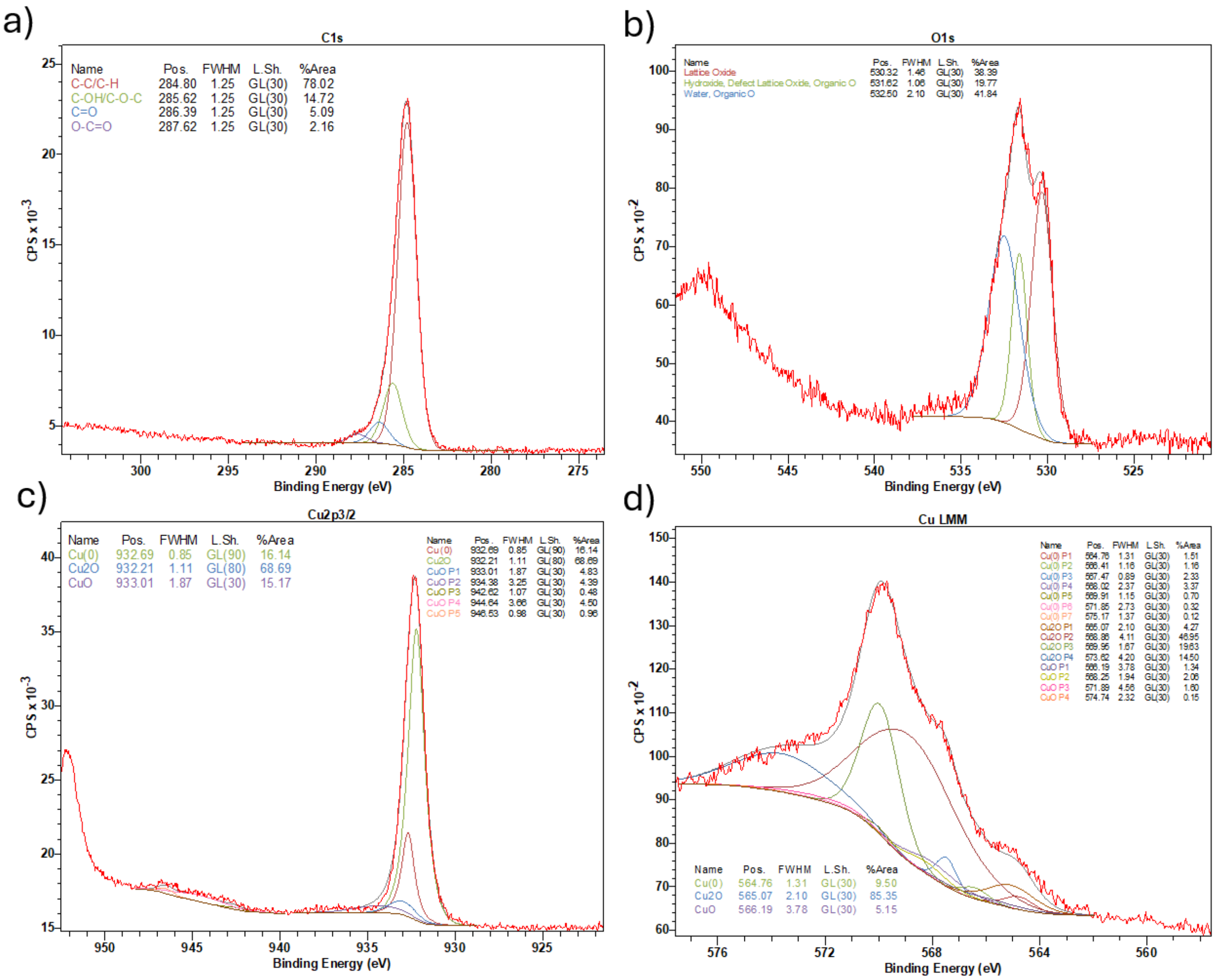

Figure S13. XPS of Cu rods cleaned using the lemon-baking soda method showing spectra of a) C 1s, b) O 1s, c) Cu $2p^{3/2}$, and d) Cu LMM. Peak fits are shown along with peak positions, FWHM, line shapes, and % Area.

The lemon-baking soda cleaning method was as follows: initial rod surface was prepared by rinsing in nanopure H_2O (18.2 MΩ · cm, Barnstead), rinsing under EtOH, and drying under streaming N_2 (g). Subsequently, baking soda (2.5 g) was dissolved in 1.2 mL of lemon juice to make a slightly abrasive paste. The paste was rubbed over the Cu surface using a new clean microfiber cloth. Subsequently, the rod was rinsed in nanopure H_2O (18.2 MΩ · cm, Barnstead), rinsed under EtOH, dried under streaming N_2 (g), and stored under inert atmosphere until examination by XPS following established XPS acquisition methodology for Cu BBs. XPS Cu $2p^{3/2}$ results showed CuO oxidation was reduced to 15.2 % area coverage with Cu_2O and Cu (0) dominating the remainder of the surface. Cu LMM reveals Cu_2O was more abundant that Cu (0). Both the C 1s and O 1s spectra indicate that organic contamination on the Cu surface reduced significantly compared to the pure Cu rod.

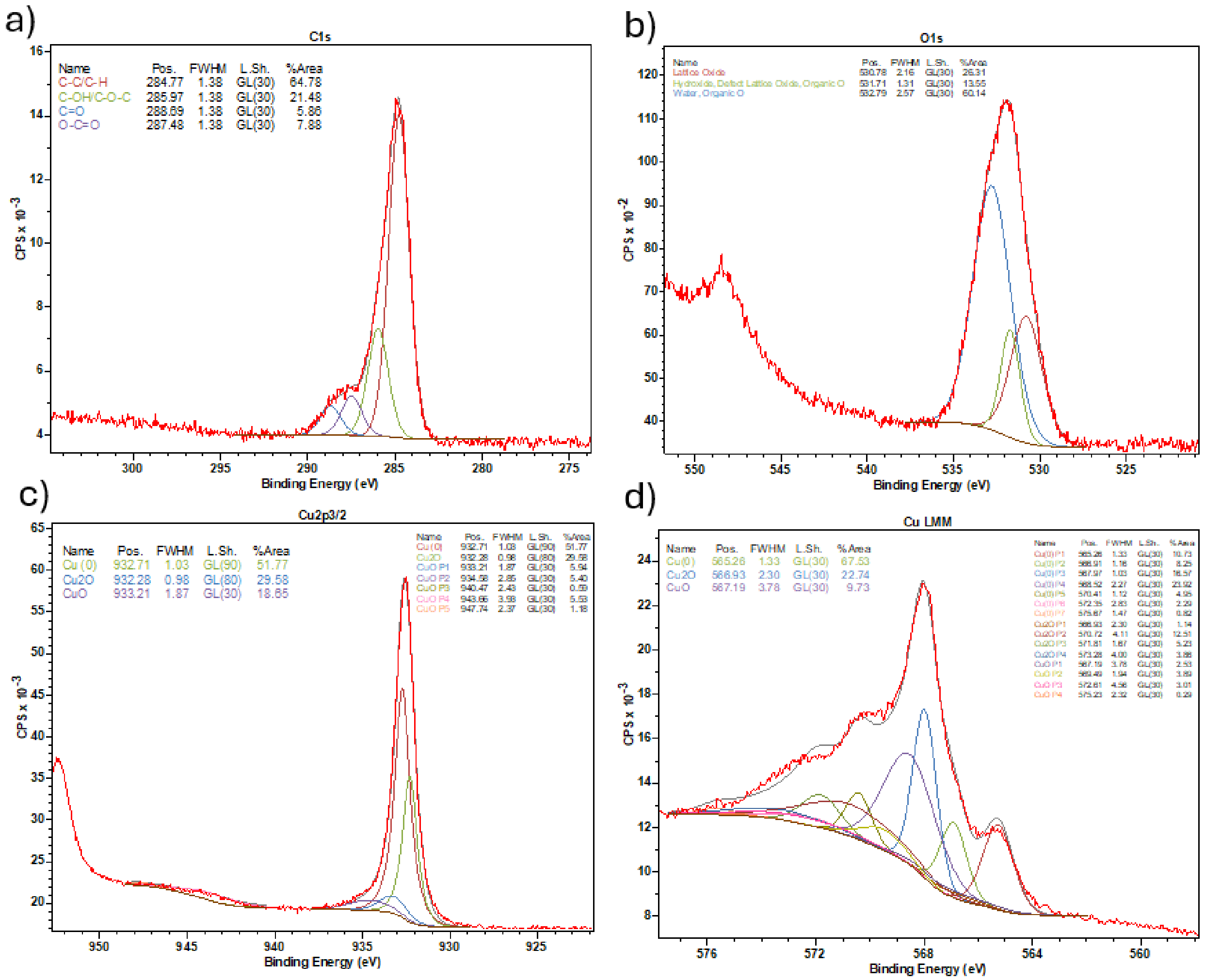

Figure S14. XPS of Cu rods cleaned using the citric acid method showing spectra of a) C 1s, b) O 1s, c) Cu $2p^{3/2}$, and d) Cu LMM. Peak fits are shown along with peak positions, FWHM, line shapes, and % Area.

The citric acid cleaning method was as follows: initial rod surface was prepared by rinsing in nanopure H_2O (18.2 MΩ · cm, Barnstead), rinsing under EtOH, and drying under streaming N_2 (g). Subsequently, Cu was thrice submersed in aqueous citric acid (11.1 % w/v) for 3 minutes and rinsed in nanopure H_2O (18.2 MΩ · cm, Barnstead) followed by a single rinse with 200 proof EtOH, drying under streaming N_2 (g), and storage under inert atmosphere until examination by XPS following established XPS acquisition methodology for Cu BBs. XPS Cu $2p^{3/2}$ results showed CuO oxidation was reduced to 18.6 % area coverage with Cu_2O and Cu (0) dominating the remainder of the surface. Cu LMM reveals that Cu (0) made up a majority of the surface. Both the C 1s and O 1s spectra indicate that organic contamination on the Cu surface reduced significantly compared to the pure Cu rod.

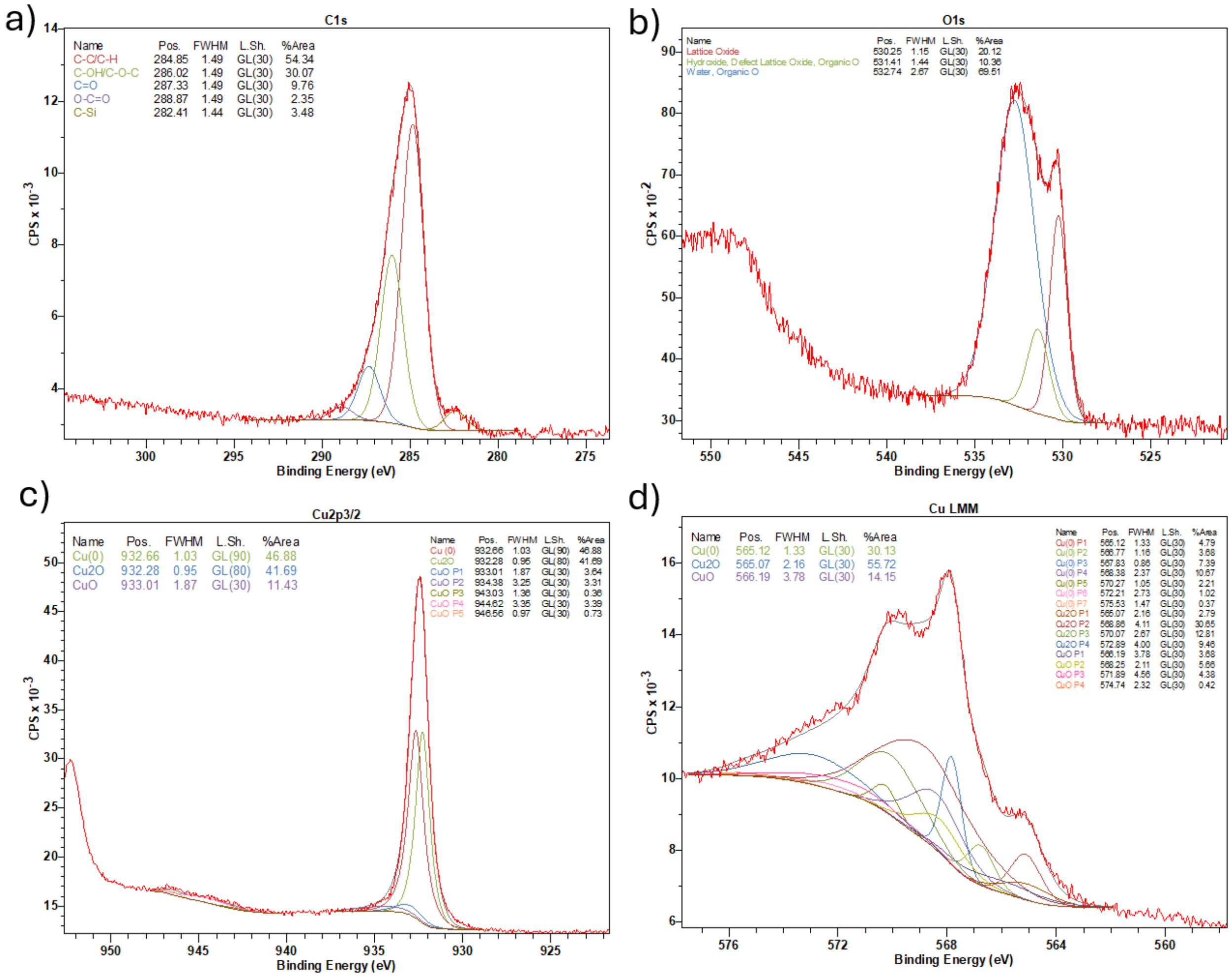

Figure S15. XPS of Cu rods cleaned using the sanding method showing spectra of a) C 1s, b) O 1s, c) Cu $2p^{3/2}$, and d) Cu LMM. Peak fits are shown along with peak positions, FWHM, line shapes, and % Area.

The sanding cleaning method was as follows: initial rod surface was prepared by rinsing in nanopure H_2O (18.2 MΩ · cm, Barnstead), rinsing under EtOH, and drying under streaming N_2 (g). Subsequently, Cu was sanded down with Imperial Wet or Dry 40IQ paper (3M) followed by a single rinse with 200 proof EtOH, drying under streaming N_2 (g), and storage under inert atmosphere until examination by XPS following established XPS acquisition methodology for Cu BBs. XPS Cu $2p^{3/2}$ results showed CuO oxidation was reduced to 11.0 % area coverage with Cu_2O and Cu (0) dominating the remainder of the surface. Cu LMM reveals that Cu_2O made up a majority of the surface. Both the C 1s and O 1s spectra indicate that organic contamination on the Cu surface reduced significantly compared to the pure Cu rod. The C 1s spectra further shows silica bound to carbon suggesting some contamination of the surface by sandpaper components.

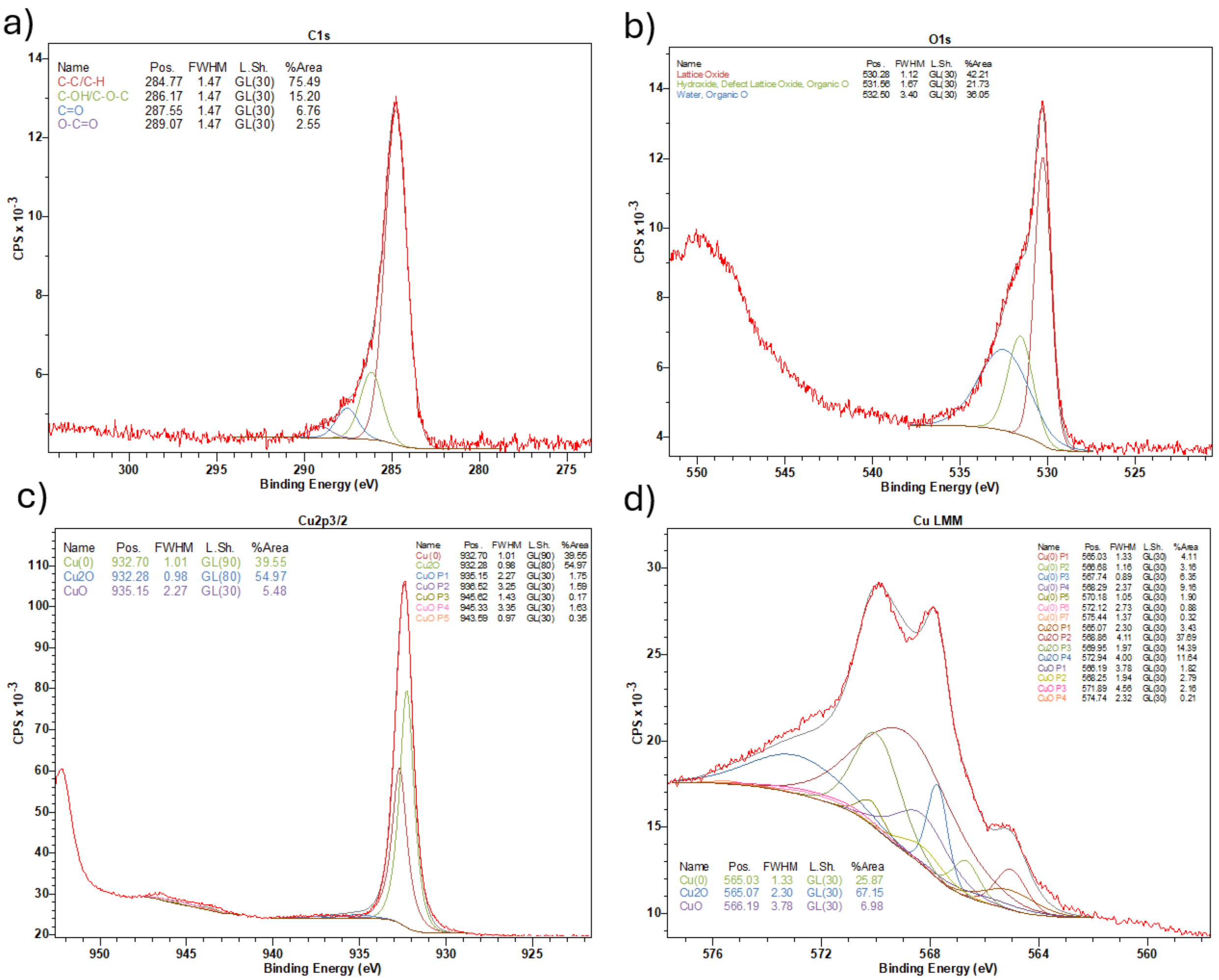

Figure S16. XPS of Cu rods cleaned using the vinegar method showing spectra of a) C 1s, b) O 1s, c) Cu $2p^{3/2}$, and d) Cu LMM. Peak fits are shown along with peak positions, FWHM, line shapes, and % Area.

The vinegar cleaning method was as follows: initial rod surface was prepared by rinsing in nanopure H_2O (18.2 MΩ ∙ cm, Barnstead), rinsing under EtOH, and drying under streaming N_2 (g). Subsequently, a solution of white vinegar (15 mL), nanopure H_2O (15 mL, 18.2 MΩ ∙ cm, Barnstead), and NaCl (0.5 g) was made and used to wet a new clean microfiber cloth which was then used to rub the Cu surface. This was followed by a rinsing in nanopure H_2O (18.2 MΩ ∙ cm, Barnstead), rinsing with 200 proof EtOH, drying under streaming N_2 (g), and storage under inert atmosphere until examination by XPS following established XPS acquisition methodology for Cu BBs. XPS Cu $2p^{3/2}$ results showed CuO oxidation was reduced to 5.5 % area coverage with Cu_2O and Cu (0) dominating the remainder of the surface. Cu LMM reveals that Cu_2O made up a majority of the surface. Both the C 1s and O 1s spectra indicate that organic contamination on the Cu surface reduced significantly compared to the pure Cu rod with superior results to the reduction achieved by all former cleaning methods.

7.2 Cleaned Copper Surface After 10 Days of Ambient Atmosphere Exposure

To determine the extent to which the surface would re-oxidize we left the cleaned Cu rods out in ambient atmosphere for 10 days and examined them again by XPS.

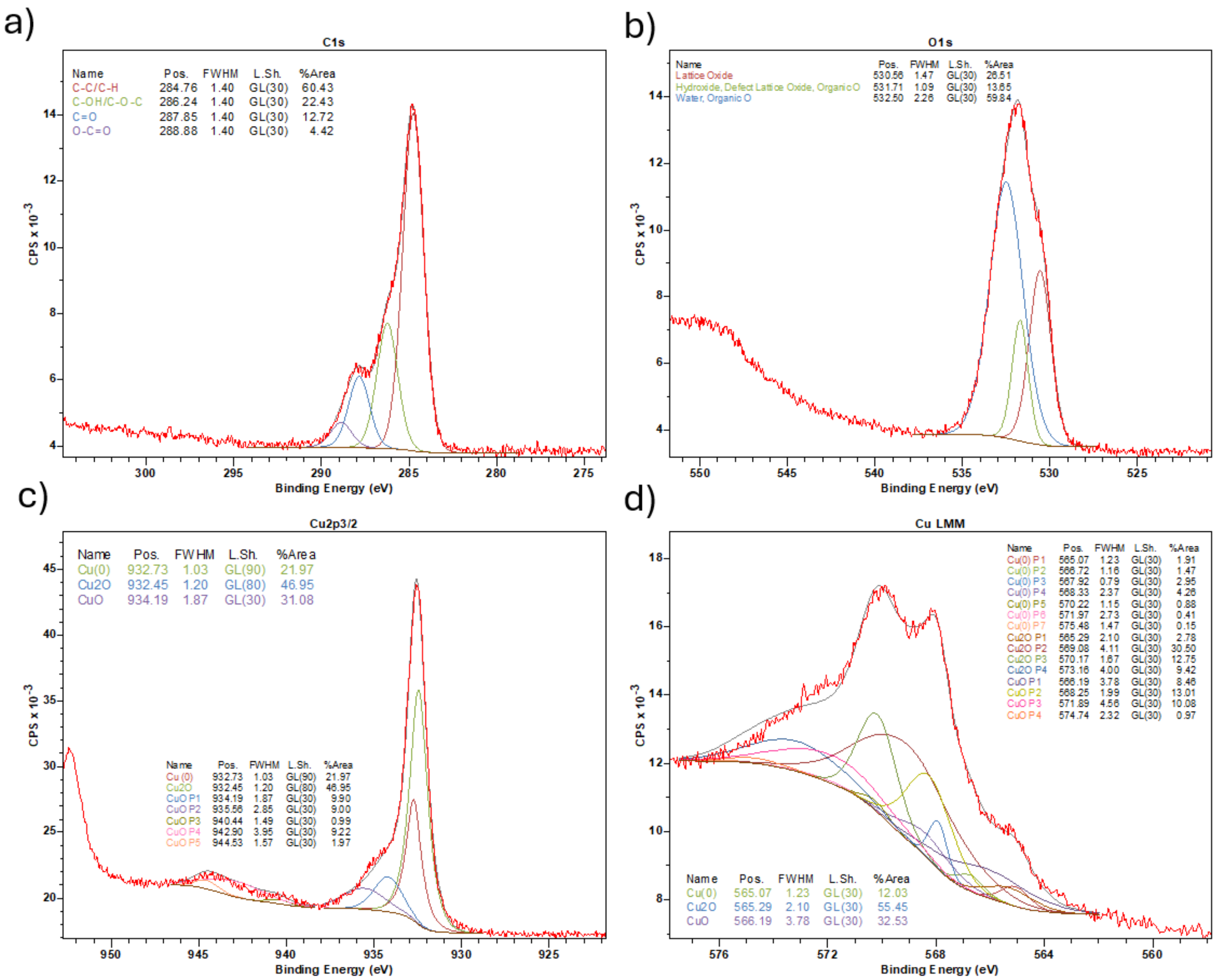

Figure S17. XPS of Cu rods cleaned using the lemon-cornstarch method after 10 days of exposure to ambient atmosphere showing spectra of a) C 1s, b) O 1s, c) Cu $2p^{3/2}$, and d) Cu LMM. Peak fits are shown along with peak positions, FWHM, line shapes, and % Area.

Cu surface cleaned using the lemon-cornstarch method showed surface CuO nearly doubling from 15.7 % to 31.1 % coverage. As previously, Cu_2O dominates over Cu (0) on the surface.

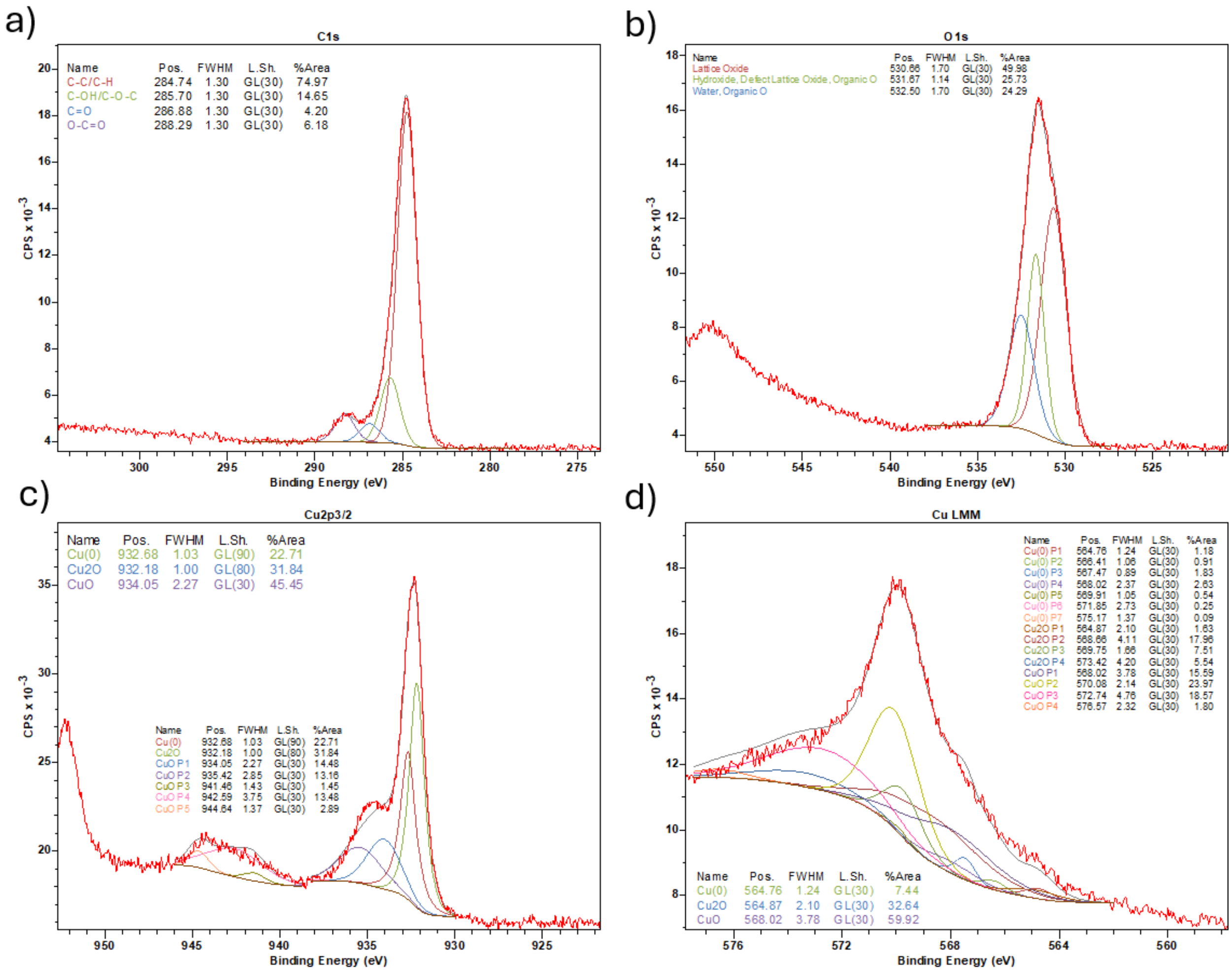

Figure S18. XPS of Cu rods cleaned using the lemon- baking soda method after 10 days of exposure to ambient atmosphere showing spectra of a) C 1s, b) O 1s, c) Cu $2p^{3/2}$, and d) Cu LMM. Peak fits are shown along with peak positions, FWHM, line shapes, and % Area.

Cu surface cleaned using the lemon-baking soda method showed surface CuO increasing from 15.2 % to 45.4 % coverage. As previously, Cu_2O dominates over Cu (0) on the surface.

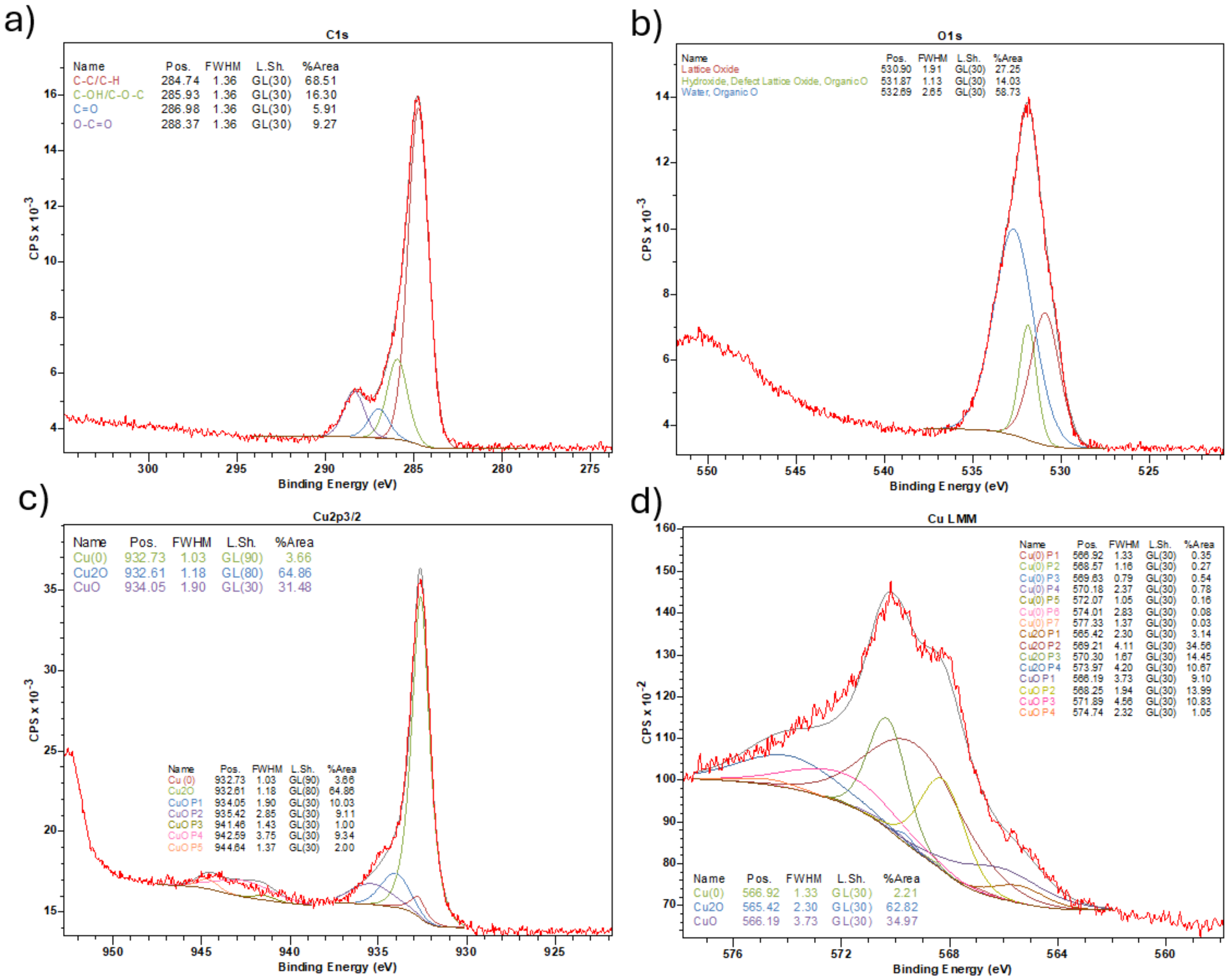

Figure S19. XPS of Cu rods cleaned using the citric acid method after 10 days of exposure to ambient atmosphere showing spectra of a) C 1s, b) O 1s, c) Cu $2p^{3/2}$, and d) Cu LMM. Peak fits are shown along with peak positions, FWHM, line shapes, and % Area.

Cu surface cleaned using the citric acid method showed surface CuO increasing from 18.6 % to 31.5 % coverage. After lengthy exposure to ambient atmosphere, Cu LMM reveals that Cu_2O has come to dominate over Cu (0) on the surface reversing the previous dominance of Cu (0) on the surface directly after cleaning.

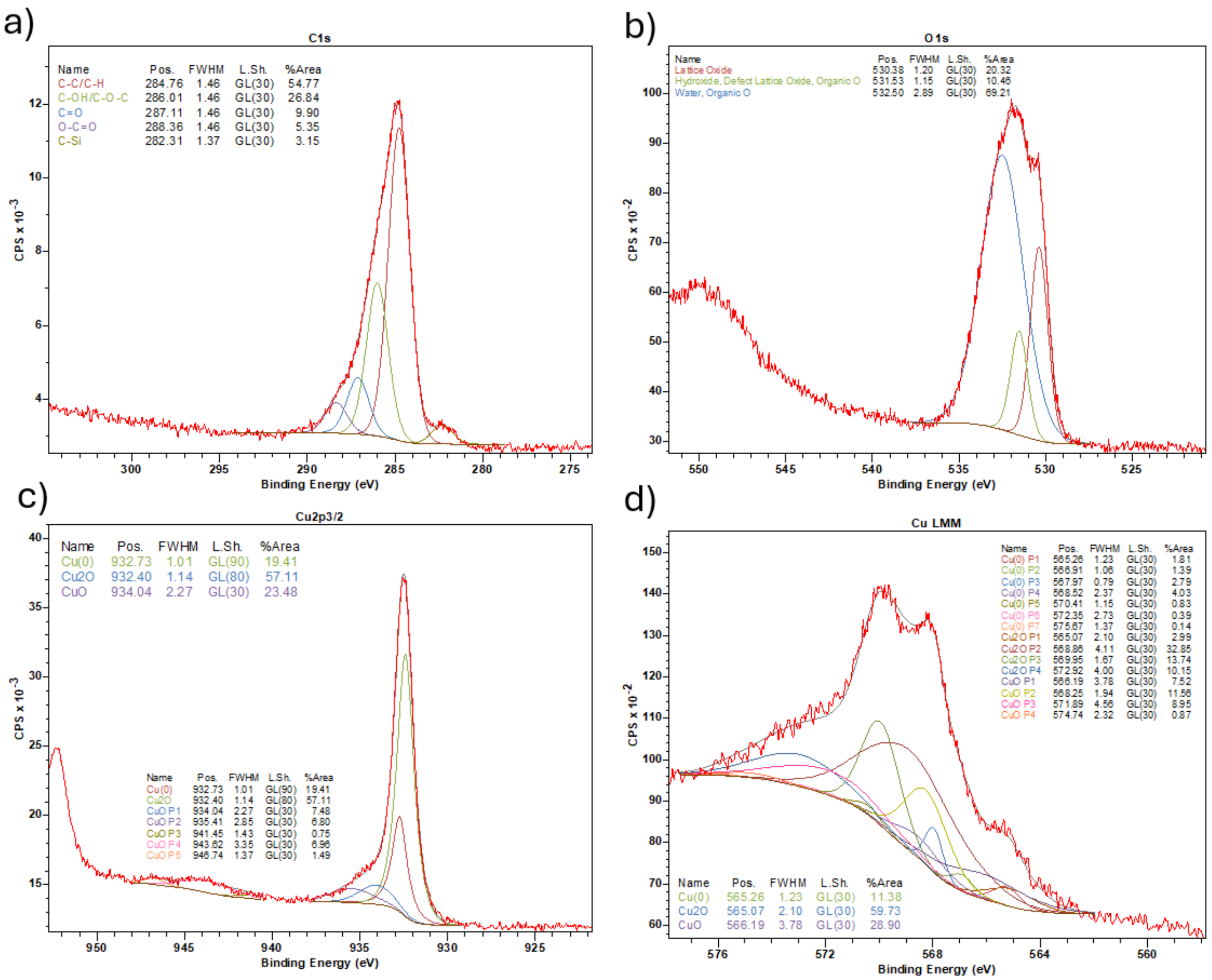

Figure S20. XPS of Cu rods cleaned using the sanding method after 10 days of exposure to ambient atmosphere showing spectra of a) C 1s, b) O 1s, c) Cu $2p^{3/2}$, and d) Cu LMM. Peak fits are shown along with peak positions, FWHM, line shapes, and % Area.

Cu surface cleaned by sanding showed surface CuO increasing from 11.0 % to 23.5 % coverage. As previously, Cu_2O dominates over Cu (0) on the surface.

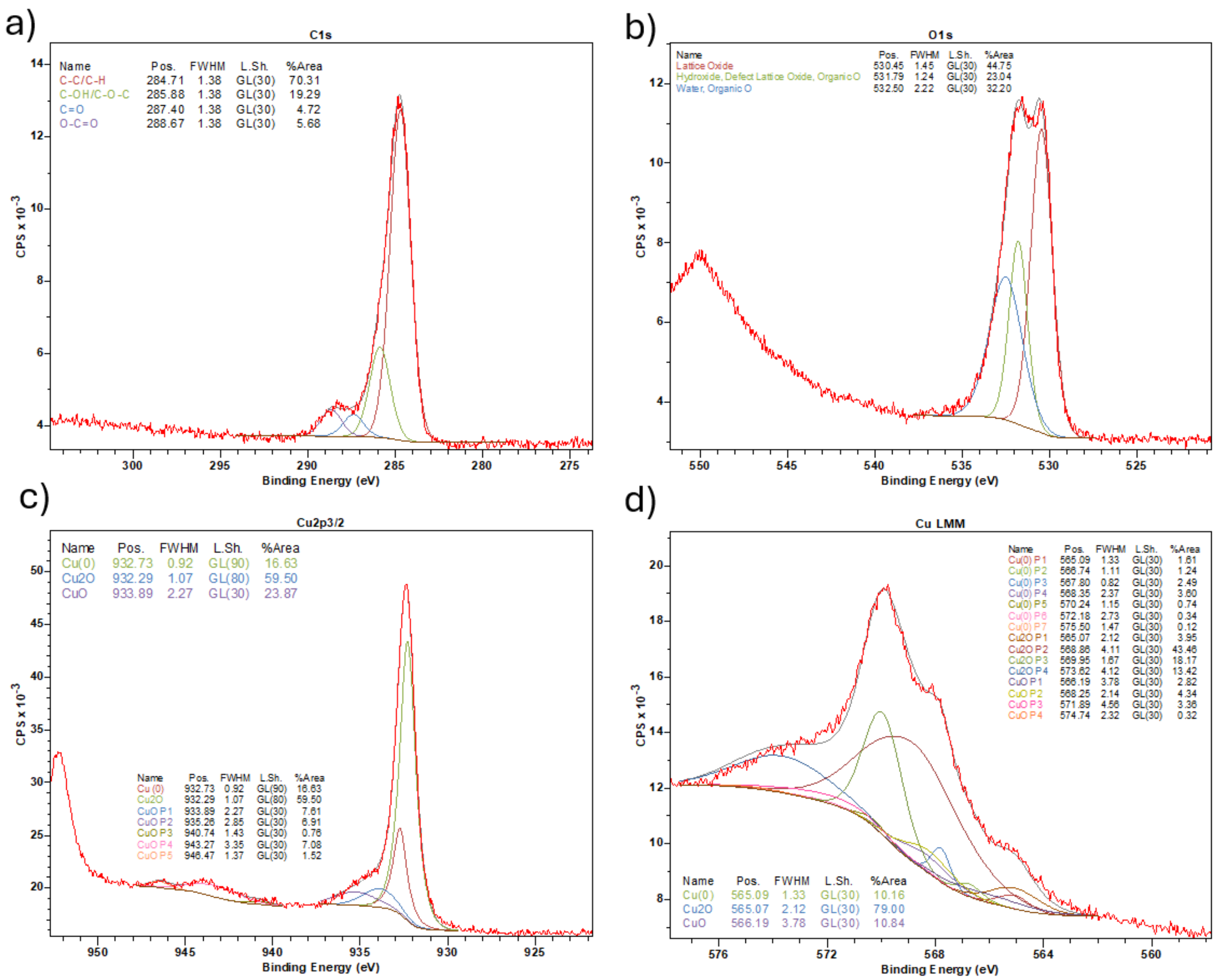

Figure S21. XPS of Cu rods cleaned using the vinegar method after 10 days of exposure to ambient atmosphere showing spectra of a) C 1s, b) O 1s, c) Cu $2p^{3/2}$, and d) Cu LMM. Peak fits are shown along with peak positions, FWHM, line shapes, and % Area.

Cu surface cleaned by vinegar showed surface CuO increasing from 5.5 % to 23.9 % coverage. As previously, Cu_2O dominates over Cu (0) on the surface.

7.3 Determination of Cleaning Process

We decided to proceed using the citric acid cleaning method for the following reasons:

1. Dominance of Cu (0) on cleaned surface over Cu_2O
2. Cost effectiveness
3. Simplicity of cleaning process removing manual rubbing of the surfaces
4. Comparatively slower formation of future oxidation than other methods
5. Relatively low contamination of the surface by cleaning residues
6. Previous establishment of the process in literature[4]

8. XPS DATA TO DETERMINE IDEAL UV OZONE OXIDATION TIME

The surface begins as ~65% solid copper metal and Cu_2O with ~35% CuO. Upon exposure to UV ozone, Cu_2O converts to the more stable CuO which is maximized at 45 minutes of exposure with

90% coverage. Following this point, CuO begins to convert back to Cu_2O. This is potentially due to destabilization of the CuO layer in the presence of excess oxygen radicals, or it may suggest more complex surface chemistry initiated by surface curvature; we did not explore the fundamental chemistry behind this effect further, as we sought merely to maximize a stable oxide layer. Thus, we choose to oxidize Cu-BBs for 45 minutes to maintain maximum oxide coverage for experiments examining oxidized surfaces. The Cu $2p^{3/2}$ peak, Cu LMM, and O 1s peaks are shown in Figure S22. The results of fitting each peak and the adventitious carbon C 1s peak are shown in Figure S23, Figure S24, and Figure S25.

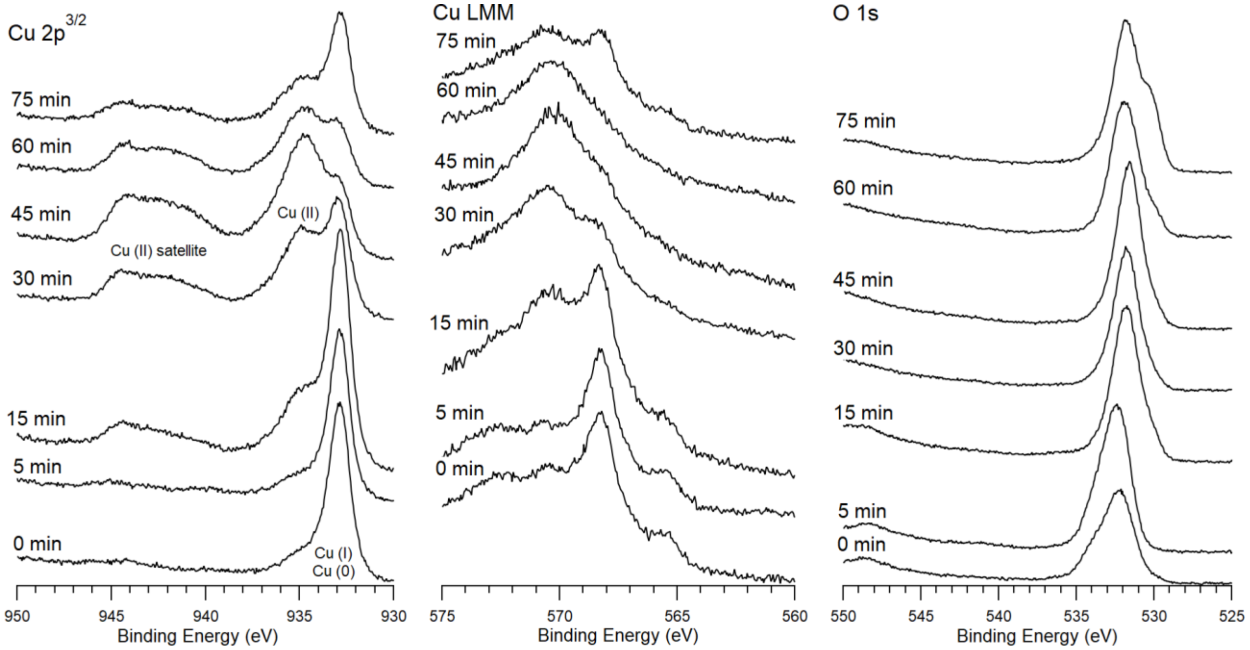

Figure S22. XPS Cu $2p^{3/2}$, Cu LMM, and O 1s peaks as a function of Cu BB exposure to UV ozone.

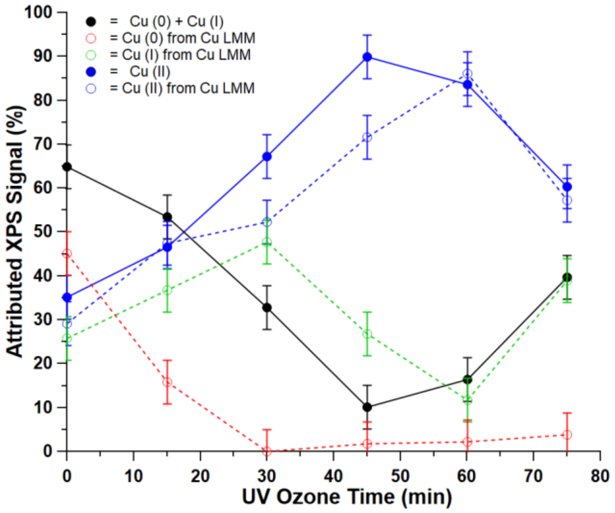

Figure S23. Copper BB surface oxidation state measured by XPS of Cu $2p^{3/2}$ and LMM peaks as a function of UV ozone exposure time. Cu (0) and Cu_2O peaks in the Cu $2p^{3/2}$ spectra overlap and judgement between the amounts of each should only be done based on Cu LMM data which can show qualitatively (fitting is too complex to be relied on rigorously in a quantitative sense) which makes up a majority of the peak.

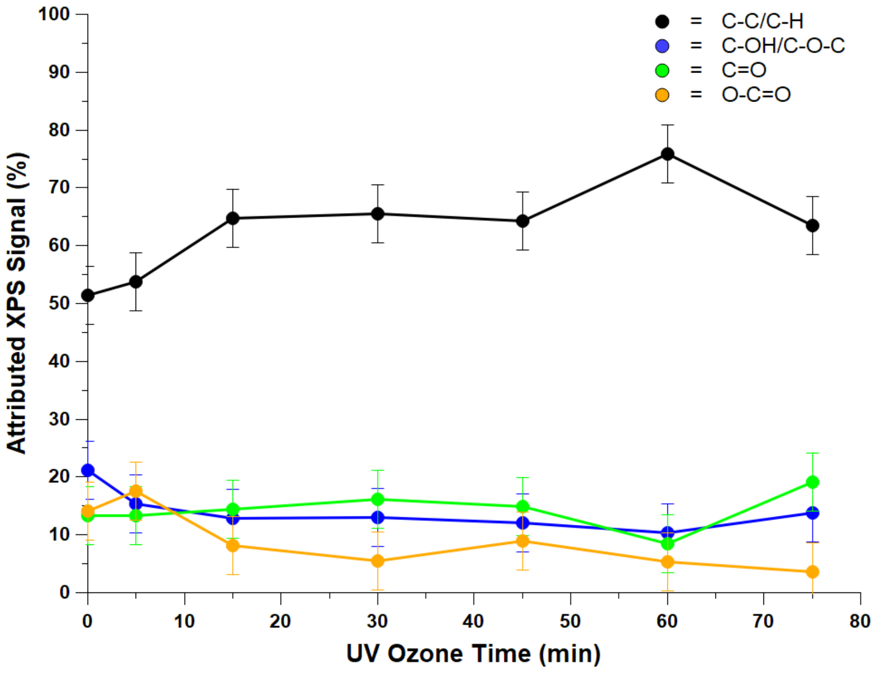

Figure S24. XPS signal % of different bonds from fits of the C 1s peak on a Cu BB as a function of Cu BB exposure to UV ozone.

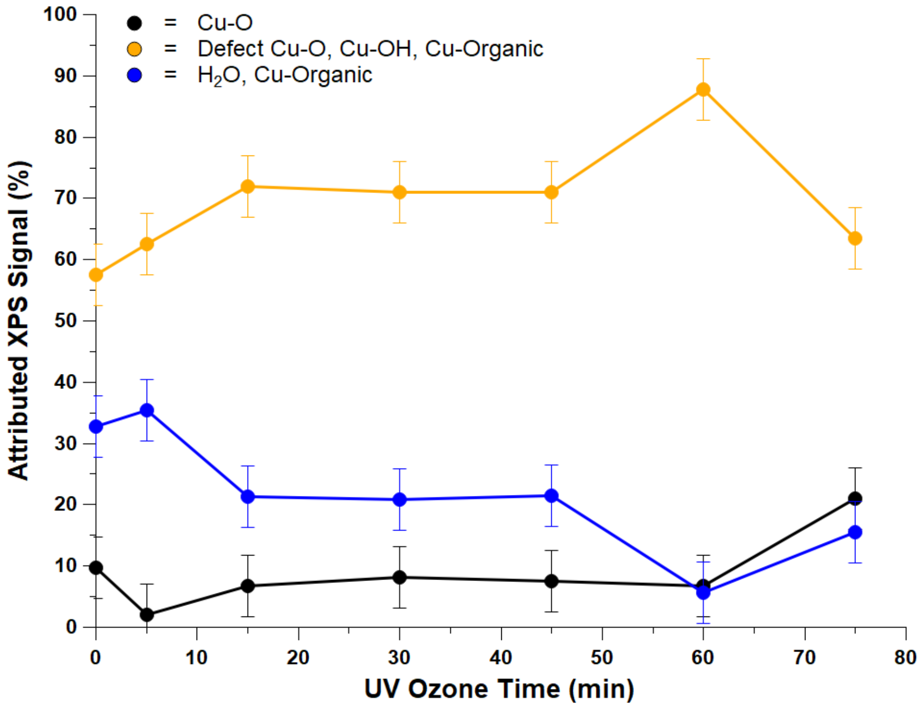

Figure S25. XPS signal % of different bonds from fits of the O 1s peak on a Cu BB as a function of Cu BB exposure to UV ozone.

9. ACETONE CLEANING FOR POST MILLING SURFACE ANALYSIS

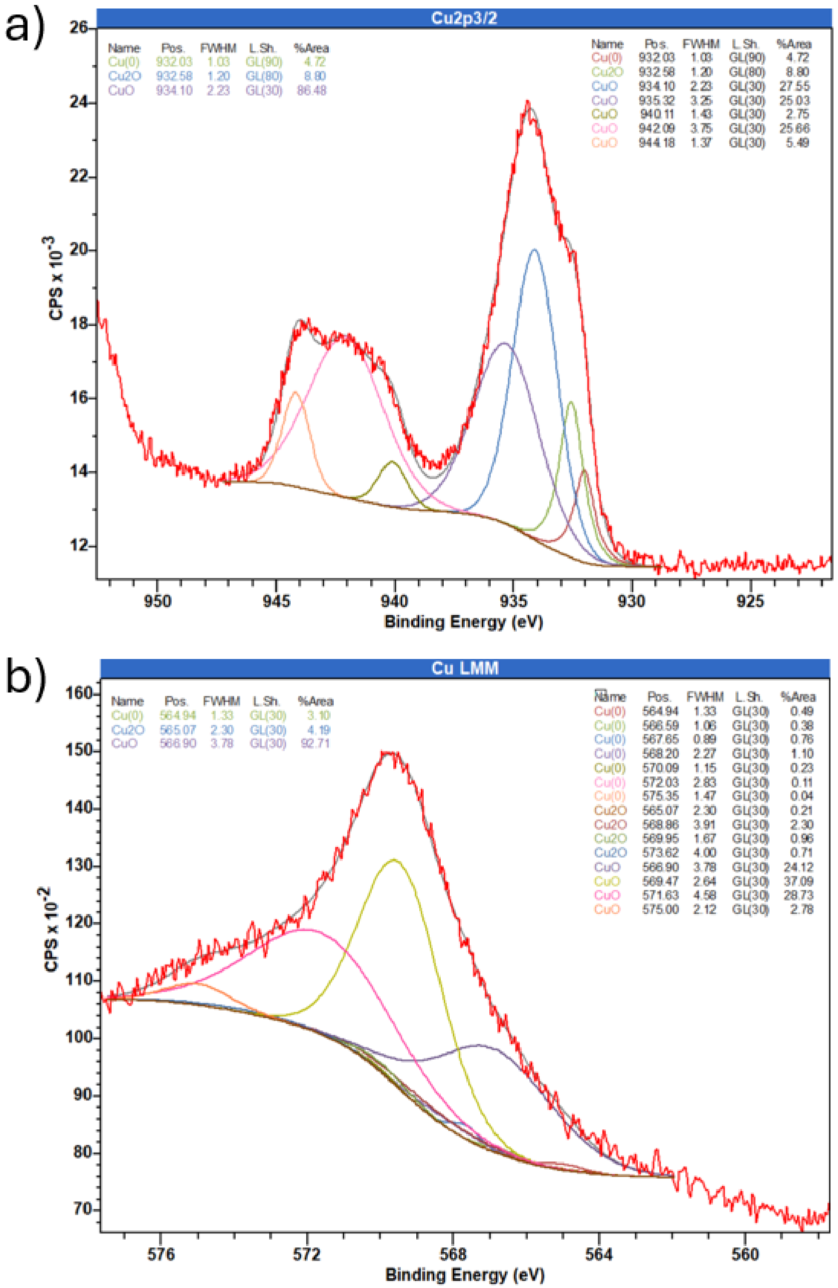

Figure S26. XPS fits of the a) Cu $2p^{3/2}$ and b) Cu LMM peaks of a native Cu BB following cleaning of the Cu BB with acetone.

A pure Cu BB was cleaned with acetone and examined by XPS to validate the inability of acetone to remove the native CuO layer from the surface as a control for tracking the changes to the Cu surface after milling for different lengths of time.

10. O 1S XPS PEAK FITTING AS A FUNCTION OF MILLING TIME

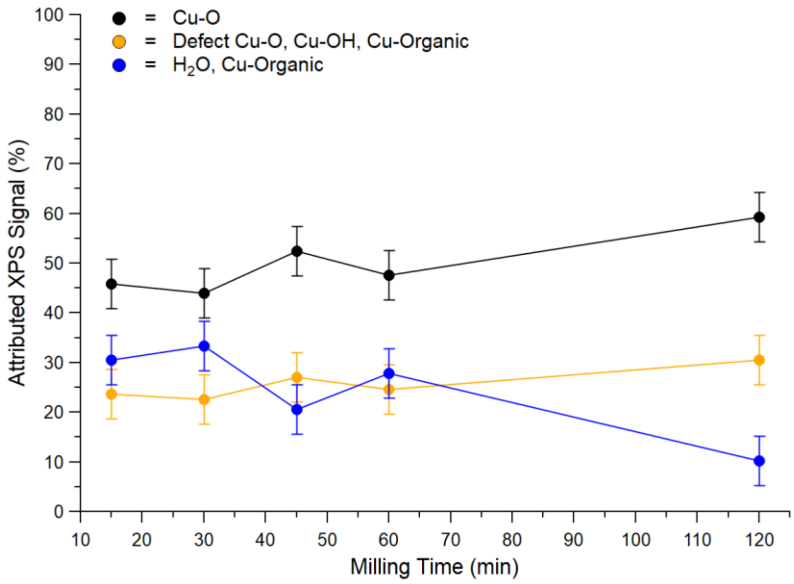

Figure S27. XPS signal % of different bonds from fits of the O 1s peak on a Cu BB as a function of milling time.

11. STUDIES ON COPPER WEAR FROM ISOLATED REGIONS OF THE JAR

Table S3. Metal wear measured by ICP-MS and reaction conversion as a function of copper location in the milling vessel.

Copper Location	Cr amount (µg/g)	Fe amount (µg/g)	Cu amount (µg/g)	% Conversion
Middle	129 ± 4	530 ± 64	7350 ± 60	83.3
Middle	81 ± 3	334 ± 55	8610 ± 90	N/A
Middle	65 ± 2	316 ± 51	7460 ± 90	78.1
Ends	79 ± 3	358 ± 54	8180 ± 150	77.5
Ends	59 ± 2	262 ± 40	9740 ± 190	N/A
Ends	60 ± 2	284 ± 72	14950 ± 200	77.3

Table S4. Comparison of copper wear and reaction conversion of isolating the copper metal on different regions of the jar.

Measurement	Cu in the Middle	Cu at the End Caps
Surface Area (mm^2)[a]	1425.7	384.3
Average amount of copper in powder (µg g^{-1})[b]	23420 (SD = 569)	32870 (SD = 2895)
Copper wear per unit of surface area (µg mm^2 g^{-1})[b]	16.4	85.5
Conversion (%)[c]	80.7	77.4

[a] calculated from the Cu inserts dimensions; [b] average from three trials, [c] average from two trials

12. KINETIC STUDIES OF Cu WEAR

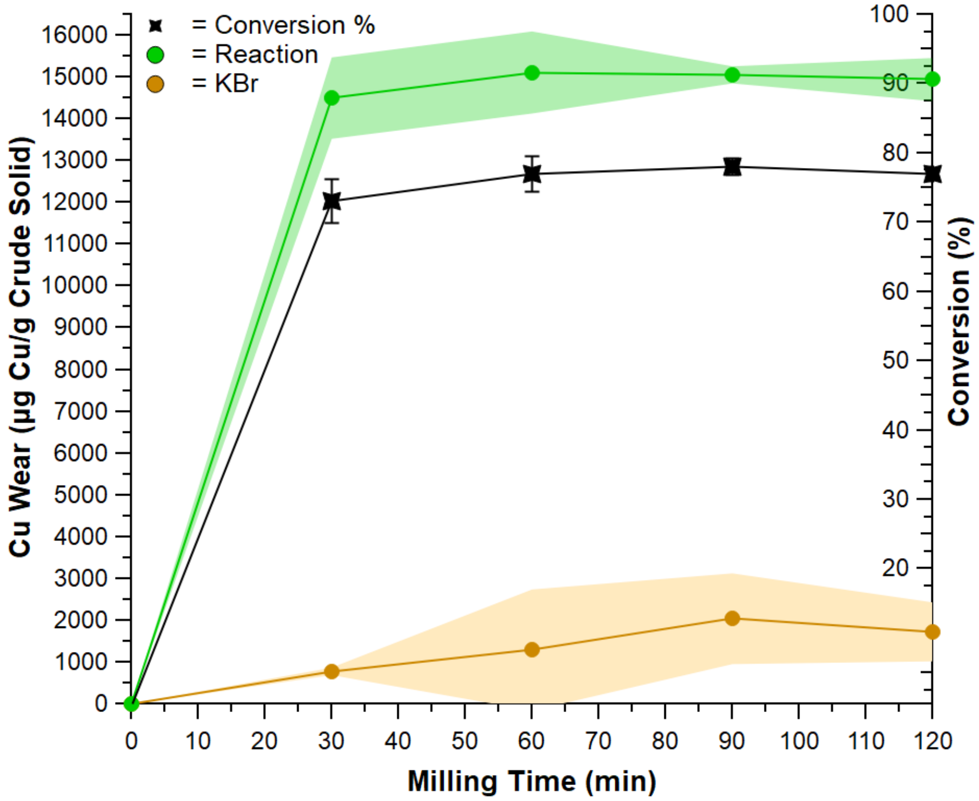

Figure S28. Measured Cu wear as a function of milling time during the reaction and in inert KBr powder. Each point represents an average of triplicate measurements and shaded regions or error bars show the standard deviation.

Wear from the Cu BBs into the powder was recorded at 30 minute increments during the reaction and separately with Cu BBs (20 total) milled in the presence of inert KBr powder (0.248 g). Results are shown in **Figure S28.** Measured Cu wear as a function of milling time during the reaction and in inert KBr powder. Each point represents an average of triplicate measurements and shaded regions or error bars show the standard deviation.Figure S28. Copper wear is accelerated by the presence of the reaction's reagents, suggesting wear kinetics are not easily translatable between systems in mechanochemical milling. Overall, copper wears quickly during the reaction within the first 30 minutes and then stabilizes at approximately 14500 μg Cu/g of crude powder. We have attempted ex-situ studies to capture the kinetics of tolbutamide formation further previously using *p*-toluenesulfonamide (214 mg, 1.25 mmol), *n*-butyl isocyanate (140.8 μL, 1.25 mmol), and traditional catalyst $CuCl_2$ (0.0017 g, 1 mol%) in 25 mL SS jars with 15 mm SS balls from Retsch milling. As shown in Figure S29, reaction repeatability is poor at short time scales making it difficult to capture the process accurately. The reaction will occasionally reach high yields at short time scales or on other occasions will require significant milling before reaching full conversion. Unfortunately, this behavior precludes simple kinetic studies of the reaction under more complex direct mechanocatalytic conditions at present. *In-situ* setups and studies are currently out of the financial scope of this manuscript. As a result, we maintained 120 minute timescales which are sufficient to ensure accurate comparison between different types of copper-based catalysts and proceeded with investigations along those lines.

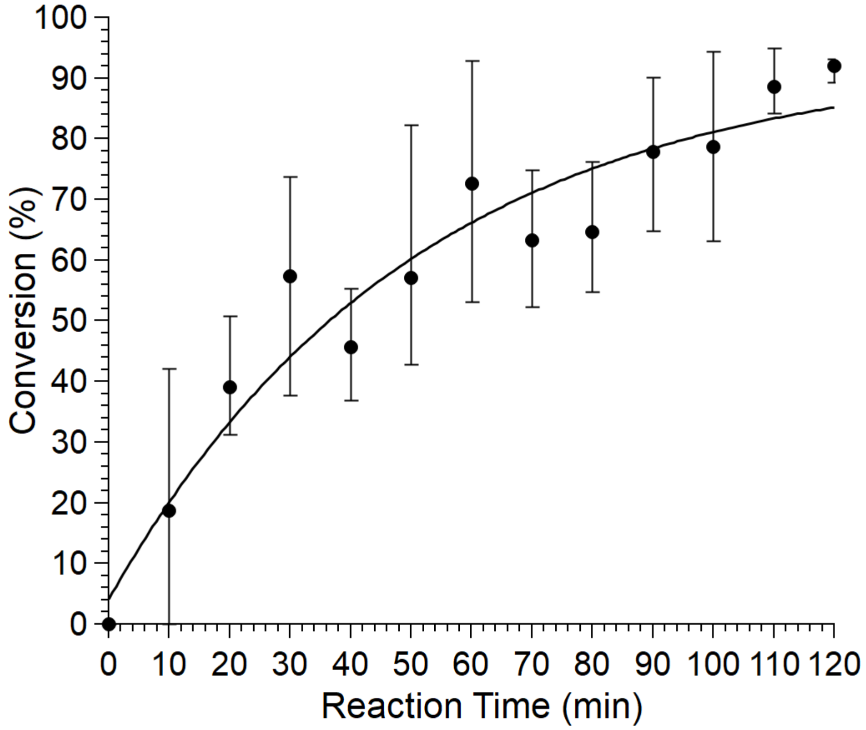

Figure S29. Kinetics of the tolbutamide transformation during milling using $CuCl_2$ catalyst (1 mol%). Each point represents an average of six replicate measures and error bars show a single standard deviation.

13. ATTEMPTING TO ISOLATE $Cu(OH)_2$ FORMATION IN THE POWDER

13.1 Raman Spectroscopy

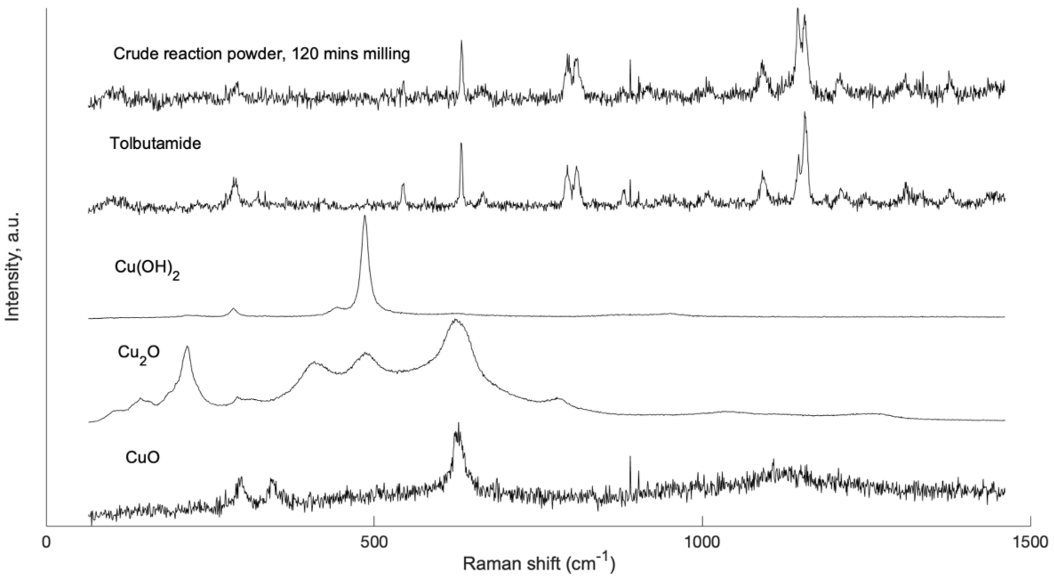

Figure S30. Raman spectra of crude powder after 120 minutes of milling using 20 Cu BBs and references.

We were unable to observe Raman bands corresponding to any of the copper oxide species (see Figure S30). We were able to locate spots that visually look like copper flakes (~10 μm large) but spectra on these flakes showed a strong background and we are not able to resolve any Raman bands from any of the copper species (see Figure S31). Cu_2O can be located in powder samples from CuBBs milled in KBr (see Figure S32); but we were unable to locate CuO and $Cu(OH)_2$ species potentially due to weaker Raman signal coupled to potential interference of KBr which may prevent further copper oxidation by removing the necessary water due to its hygroscopic nature. Raman signals of CuO and $Cu(OH)_2$ on copper metal surfaces like the flakes observed herein are known to be relatively weak, so it is unsurprising we cannot observe these species.[5] Given our laser spot size is 300 nm, domains of these species smaller than that value are likely to be below the detection limit for this instrument.

a)

b)

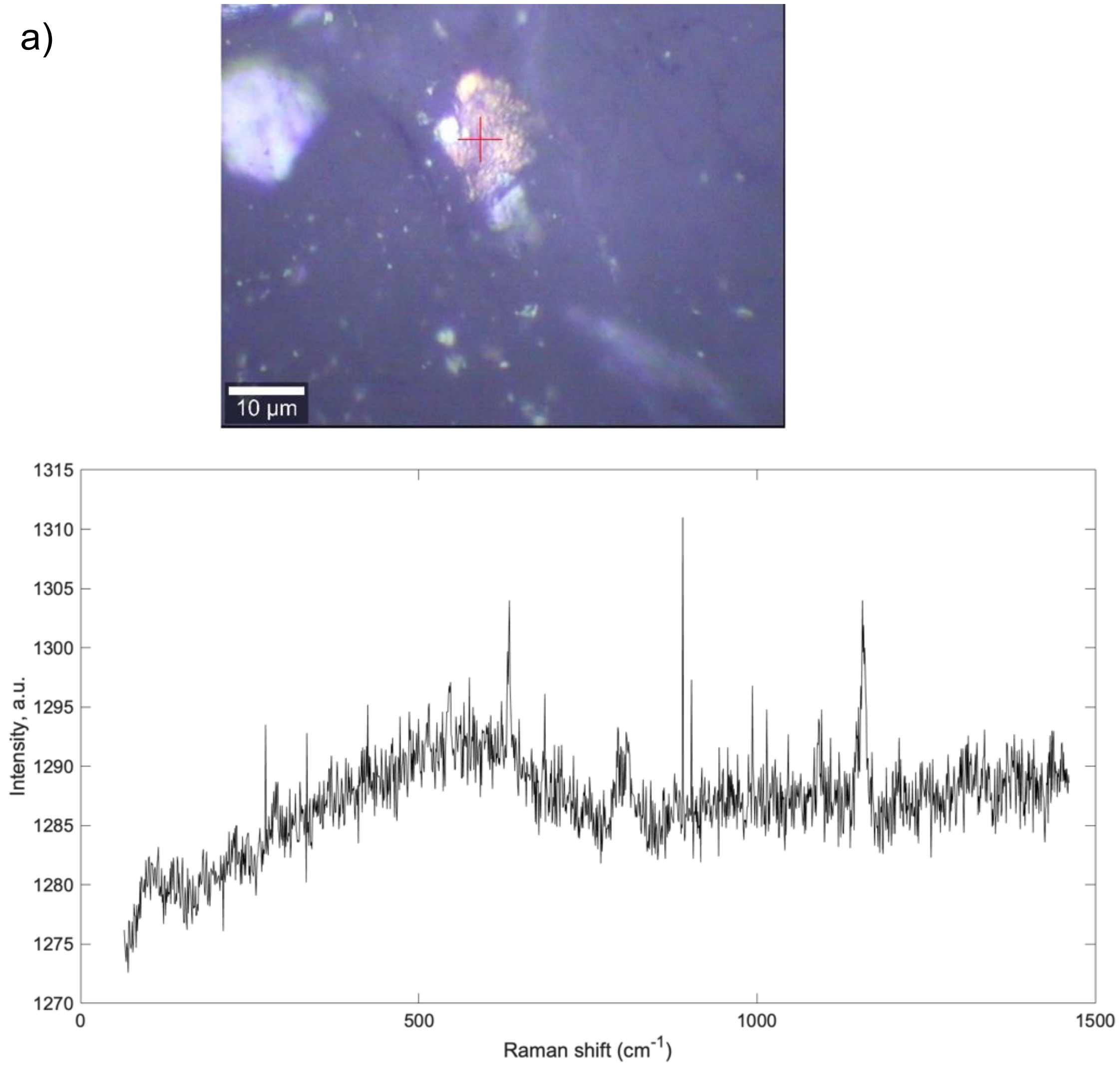

Figure S31. a) optical image of copper flake worn into crude reaction powder; b) typical Raman spectra from copper site in tolbutamide powder

a)

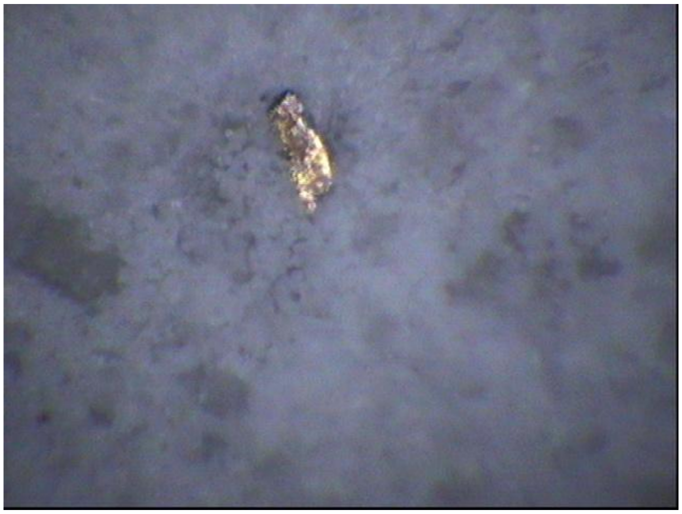

b)

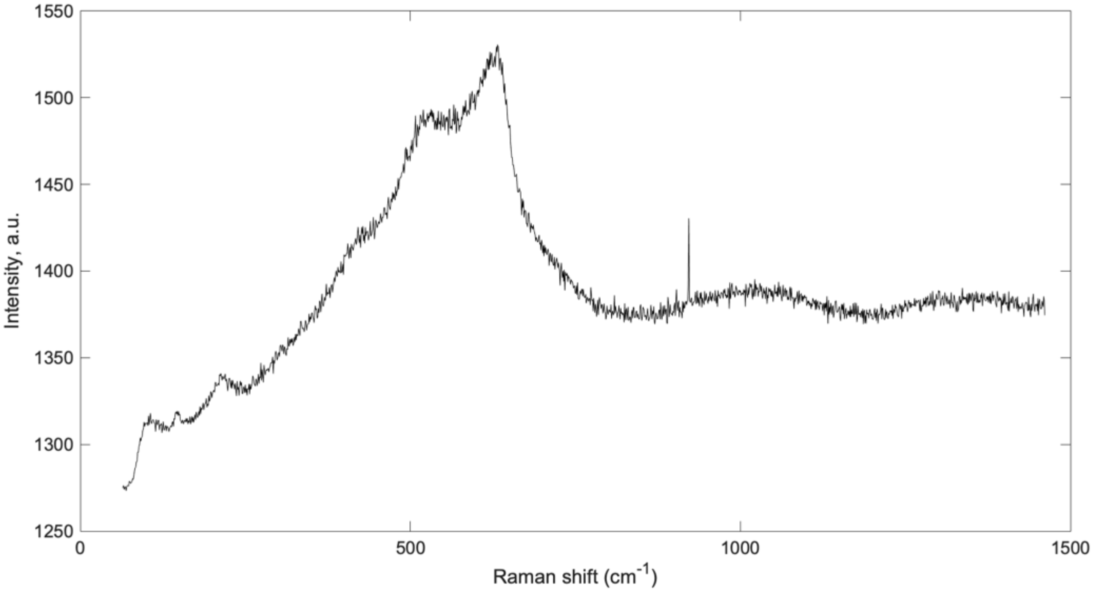

Figure S32. Raman spectra from copper site in powder from CuBBs (20x) milled in KBr (0.284 g) for 2 hrs at 30 Hz.

13.2 FTIR Spectroscopy

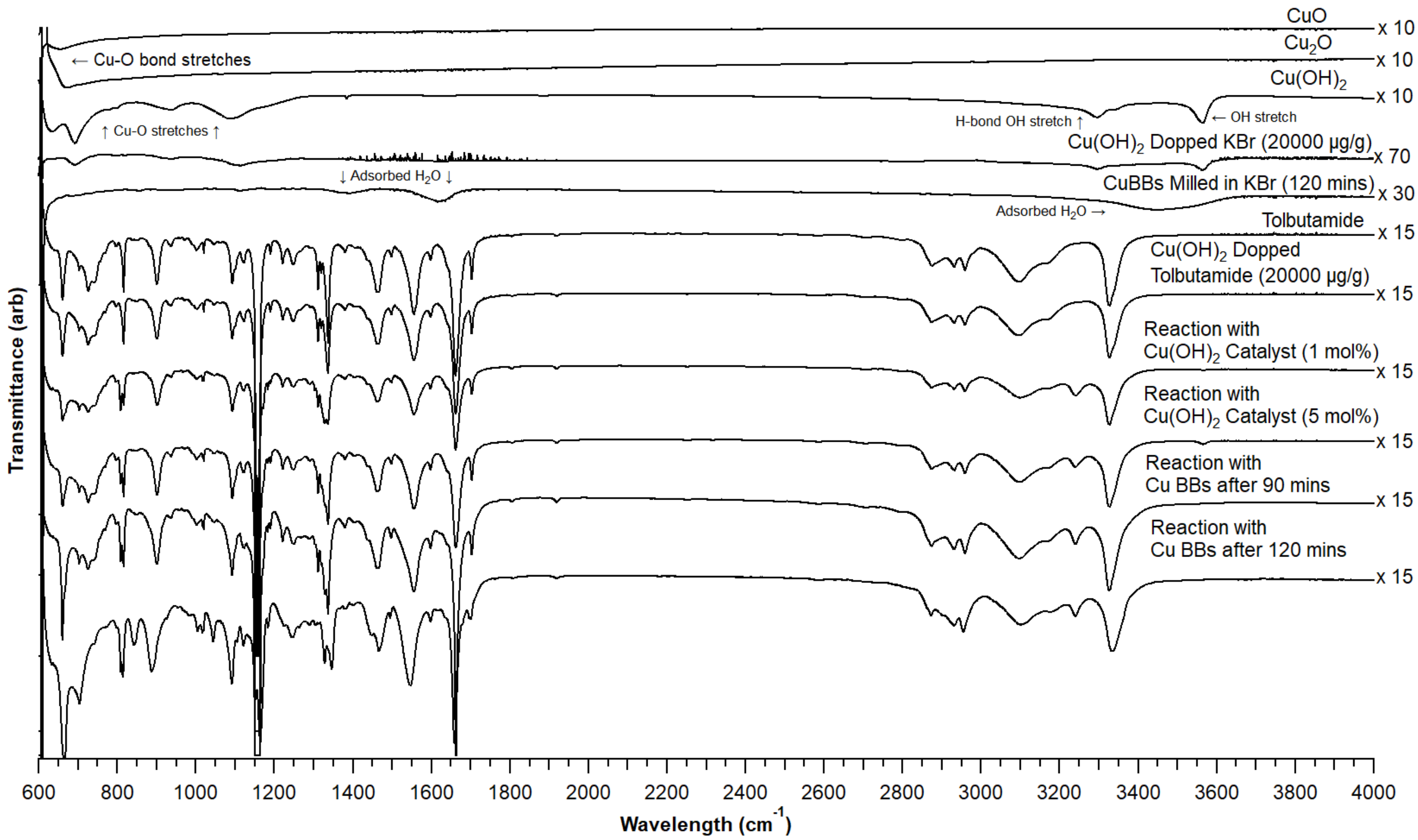

Figure S33. FTIR spectra utilized to explore the transformations of copper metal during milling. Sample identity and scaling factors between spectra are included on the right.

The presence of $Cu(OH)_2$ can be validated by peaks below 700 cm^{-1} associated with Cu-O vibrations, the peak at ~3430 cm^{-1} representing hydrogen bonded hydroxyl stretches, and the peak at ~3570 cm^{-1} representing the free OH stretch of the hydroxyl. Peaks for CuO and Cu_2O are generally not visible being overshadowed by reagent and product peaks (see Figure S33). The only visible peak for determination of $Cu(OH)_2$ content was at 3570 cm^{-1}. Given this peak arises from a hydroxyl stretch, we are cautious to use this data to make a positive identification of the catalyst in crude samples. However, we searched for the peak appearing in samples from reactions utilizing $Cu(OH)_2$ as the catalyst in 1 and 5 mol % quantities (controls), reactions utilizing Cu BBs after 120 min and 90 minutes of milling, and Cu BBs after milling in 120 min with KBr (which serves as an IR-inactive medium to collect copper wear during the grinding process). We further explored additional controls of tolbutamide spiked with $Cu(OH)_2$ standard at amounts measured by ICPMS from Cu BB wear after 120 min of milling and KBr spiked with $Cu(OH)_2$ standard at amounts measured by ICPMS from Cu BB wear after 120 min of milling.

The $Cu(OH)_2$ hydroxyl stretch from $Cu(OH)_2$ spiked in KBr at typical wear amounts can be observed by FTIR (see Figure S33). Unfortunately, Cu BBs milled in KBr simply show water adsorbed from the atmosphere which overshadows any potential $Cu(OH)_2$ peaks formed (see Figure S33). In $Cu(OH)_2$-spiked tolbutamide and samples using $Cu(OH)_2$ directly as the catalyst in typical quantities, resolution of the $Cu(OH)_2$ peak is poor and it can often not be easily discerned

without significant scaling enhancement often into the spectral background features (see Figure S33 and Figure S34).

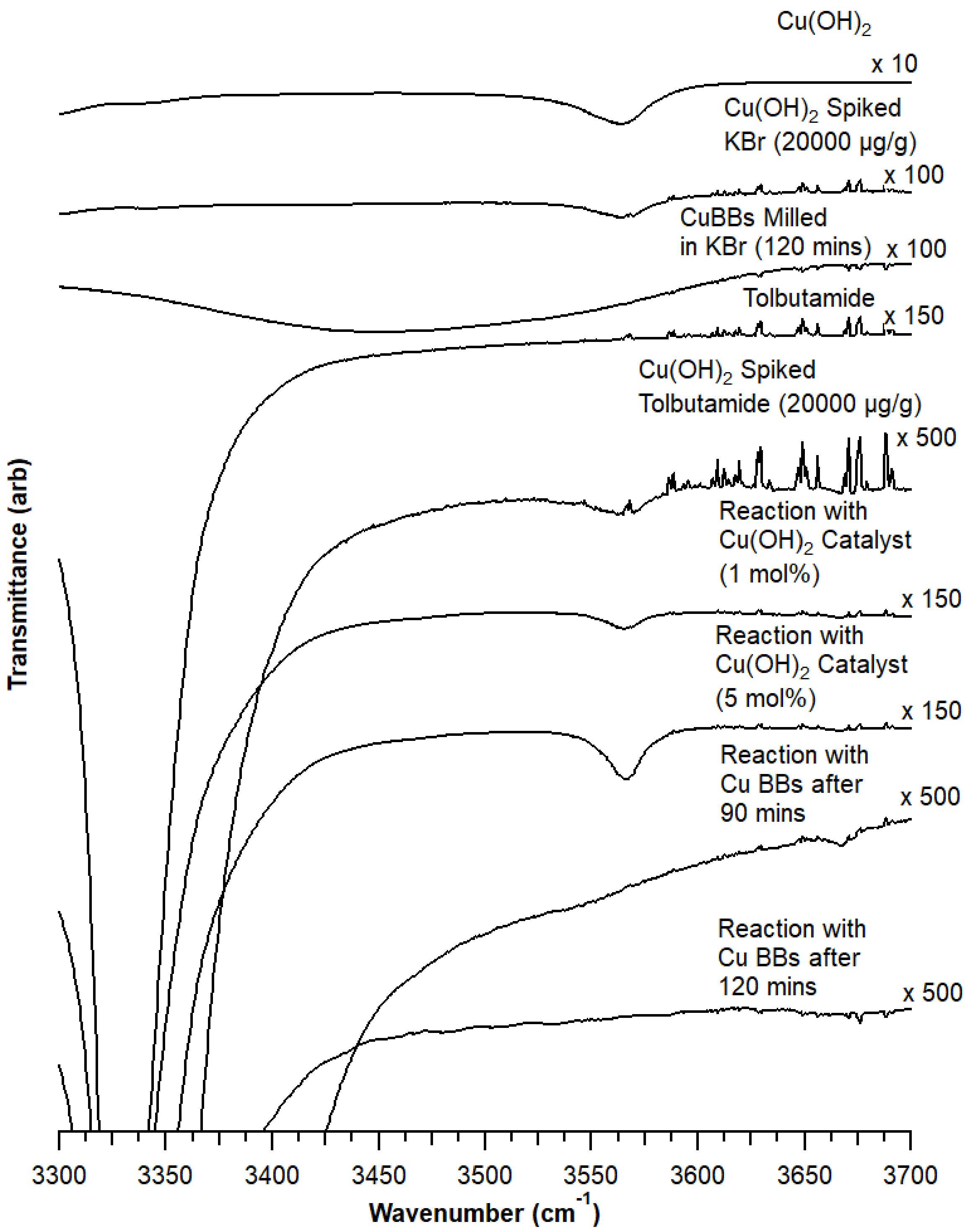

Figure S34. FTIR spectra utilized to explore the transformations of copper metal during milling with scaling enhancement around the $Cu(OH)_2$ hydroxyl stretch. Sample identity and scaling factors between spectra are included on the right.

Samples utilizing $Cu(OH)_2$ in 1 and 5 mol % quantities in theory provide upper and lower bounds for catalyst quantities that would yield the conversions observed with the Cu BBs while the $Cu(OH)_2$-spiked tolbutamide sample shows the expected $Cu(OH)_2$ signal that would be seen assuming full conversion of all worn copper to a hydroxide. While all of these samples show the weak $Cu(OH)_2$ 3570 cm^{-1} peak (shifted in the case of the spiked sample), it is not visible in samples from the reaction with Cu BBs at either 90 or 120 minutes (see Figure S34). This is unsurprising given the difficulties of resolving the low concentrations of even known samples and the likely weaker signal in real samples due to the lack of a defined crystallinity and incomplete conversion of the metal to the catalyst. We also suspect that Cu(II)-OH species formed during the milling process are unstable compared to solid $Cu(OH)_2$ standard and tend to convert to a more stable species in the ambient atmosphere required to prepare FTIR samples. Most $Cu(OH)_2$ species on surfaces of the metal are metastable and transform to CuO in ambient atmosphere.[6]

13.3 Powder X-ray Diffraction (PXRD)

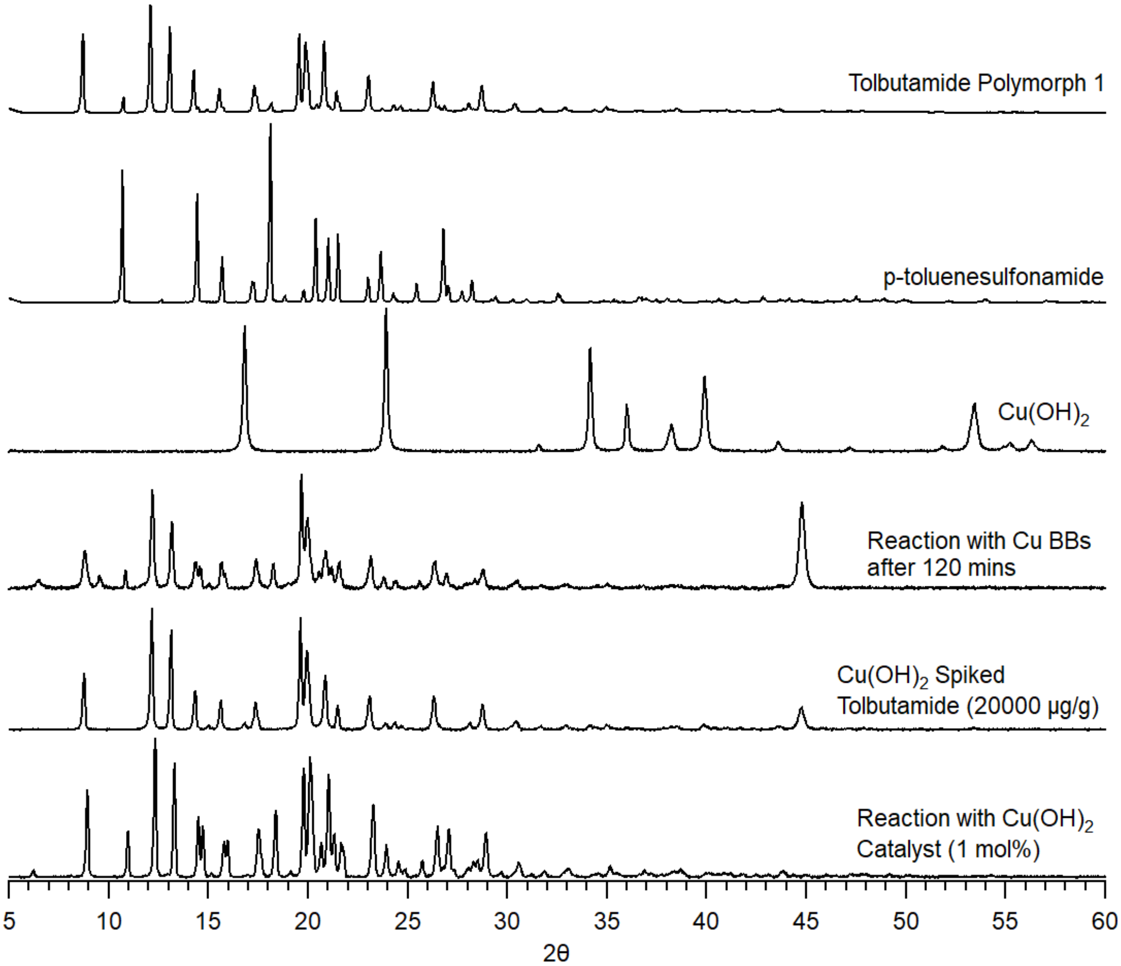

Figure S35. PXRD spectra of the crude reaction powder from milling Cu BBs in ambient atmosphere for 30 Hz compared to reference samples.

$Cu(OH)_2$ peaks could not be observed in PXRD spectra of the crude reaction mixture after 120 min of milling at 30 Hz with Cu BBs. Control samples utilizing $Cu(OH)_2$ in 1 mol % quantities and a sample of tolbutamide product spiked with $Cu(OH)_2$ in quantities matching Cu wear

measured by ICPMS (which would assume full conversion of all worn copper to a hydroxide) also fail to show any peaks. This suggests the amount of hydroxide formed in the reaction is below the instrumental detection limit.

13.4 XPS

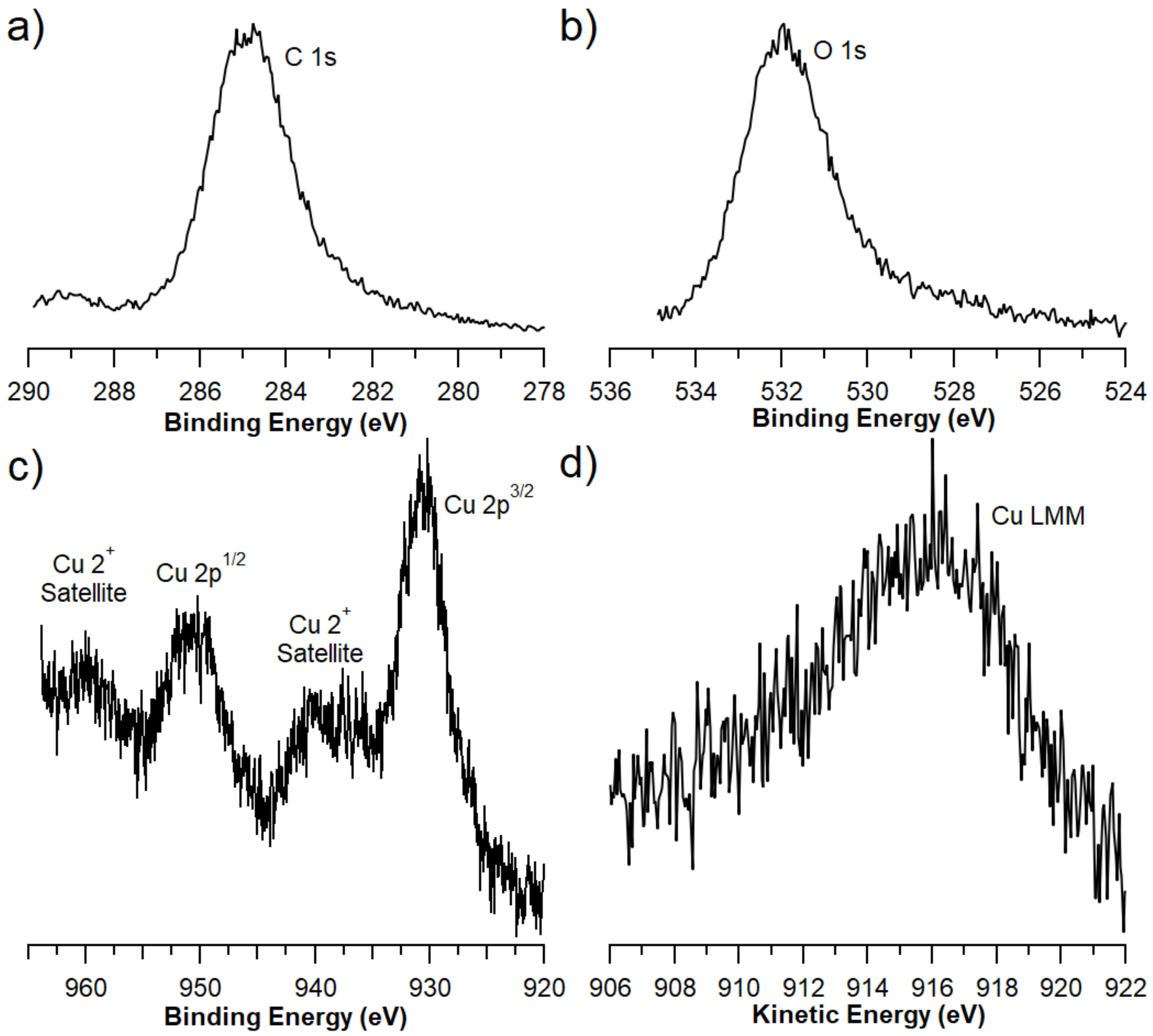

Figure S36. XPS spectra of crude reaction powder including C 1s (a), O 1s (b), Cu 2p (c), and Cu LMM (d). Binding energies were corrected for charging by 26.1 eV as calibrated to the C 1s peak shift. Reaction conditions: 20 Cu BBs, humid atmosphere, 120 min, 30 Hz.

Crude powder from the reaction with Cu BBs under humid atmosphere after 120 minutes was stored under inert atmosphere following the reaction until XPS analysis. Spectra collection followed the standard procedure mentioned in the manuscript except for the need for a few shorter survey spectra before full collection due to substantial charging of the powder under x-ray exposure causing a shift in the peak location. The resulting charge-corrected spectra are shown in Figure S36. Unfortunately, charging on the powder was severe and can be seen to severely alter peak shape. The altered peak shape prevents peak fitting to definitively determine the

identity of oxidized copper species. Furthermore, the signal to noise ratio is poor due to low copper concentration in the matrix (this data required 12 scans and 2 hours per Cu spectra). We cannot increase signal to noise beyond this point at present due to instrumental limitations and difficulty of copper reduction under prolonged x-ray exposure (access to a charge neutralizer is not available at present).[3] Qualitatively, three features of these spectra align more closely with literature for those described for $Cu(OH)_2$ than CuO.[3] Firstly, the Cu $2p^{1/2}$ satellite peak is much less intense than the Cu $2p^{1/2}$ peak.[3] Secondly, the Cu $2p^{3/2}$ satellite peak is more pointed and sharply angled upward matching the shape expected for $Cu(OH)_2$ rather than the double humped feature typical for CuO.[3] Lastly, the Cu LMM peak is more intense at kinetic energies ranging from 908 - 914 eV.[3] However, spectra is still likely a combination of various copper species and without peak fitting we are cautious to use this data at present as proof of catalyst identity.

14. DFT CALCULATIONS OF THE REACTION USING $CaCl_2$ AND $ZnCl_2$

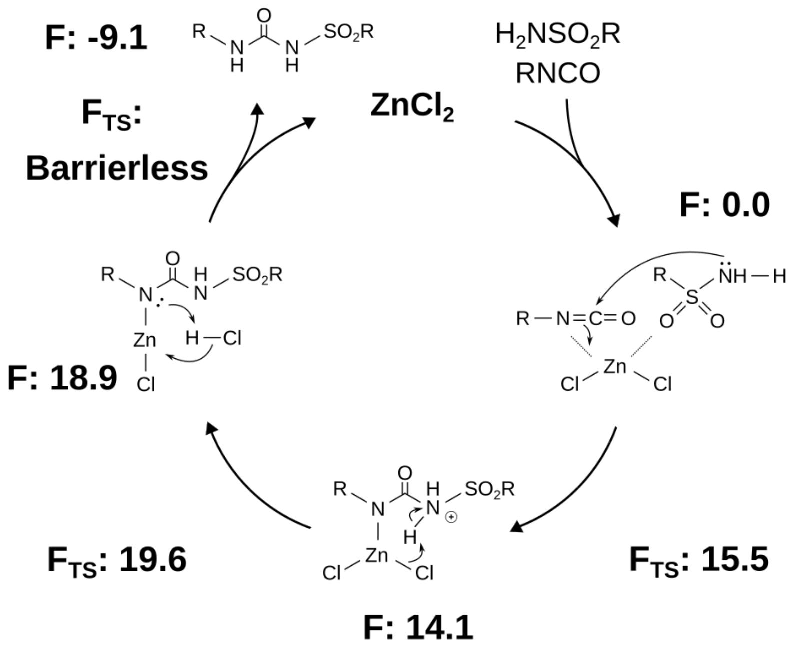

Figure S37. Mechanism for the tolbutamide synthesis using $ZnCl_2$.

The use of $ZnCl_2$ and $CaCl_2$ catalysts have been reported with yields of 6% and 0% respectively under neat milling conditions. DFT calculations at the PBE0+D3/6-311+G(d,p) level of theory with PCM solvation (ε = 8.94) were also used to investigate the mechanisms of $ZnCl_2$ and $CaCl_2$, following the scheme proposed for $CuCl_2$ in the manuscript. The energetics for $ZnCl_2$ are shown in Figure S37. We were unable to optimize the final transition state for $ZnCl_2$ in PCM. However when optimized in the gas phase, this transition state appears to be barrierless. Here nearly every step in the mechanism is less energetically favorable than that in $CuCl_2$, thereby accounting for the significantly reduced yield of $ZnCl_2$. The final intermediate for $ZnCl_2$ contains an additional unintended imaginary frequency that may impact the free energy, however the trend is also present when considering the electronic energy. In the case of $CaCl_2$, we were unable to obtain any transition states for this mechanism. Starting from the optimized $CuCl_2$ transition states, we replaced the copper atom with a calcium atom. Our attempted searches would then be optimized

to our starting tetrahedral complex. This implies that these transition states do not exist and is largely consistent with the lack of observed experimental yields.

15. DOI LINK TO RAW DATA FILES

Raw data files for results presented herein are available at the following DOI: 10.6084/m9.figshare.28410224

16. REFERENCES

(1) Floyd, K.; Gonnet, L.; Friščić, T.; Batteas, J. The role of the milling environment on the copper-catalysed mechanochemical synthesis of Tolbutamide. *RSC Mechanochemistry* **2024**, *1* (3), 289-295, 10.1039/D4MR00031E. DOI: 10.1039/D4MR00031E.

(2) Colacino, E.; Dayaker, G.; Morère, A.; Friščić, T. Introducing Students to Mechanochemistry via Environmentally Friendly Organic Synthesis Using a Solvent-Free Mechanochemical Preparation of the Antidiabetic Drug Tolbutamide. *Journal of Chemical Education* **2019**, *96* (4), 766-771. DOI: 10.1021/acs.jchemed.8b00459.

(3) Biesinger, M. C. Advanced analysis of copper X-ray photoelectron spectra. *Surface and Interface Analysis* **2017**, *49* (13), 1325-1334. DOI: https://doi.org/10.1002/sia.6239.

(4) Aromaa, J.; Kekkonen, M.; Mousapour, M.; Jokilaakso, A.; Lundström, M. The Oxidation of Copper in Air at Temperatures up to 100 °C. *Corrosion and Materials Degradation* **2021**, *2* (4), 625-640.

(5) Deng, Y.; Handoko, A. D.; Du, Y.; Xi, S.; Yeo, B. S. In Situ Raman Spectroscopy of Copper and Copper Oxide Surfaces during Electrochemical Oxygen Evolution Reaction: Identification of CuIII Oxides as Catalytically Active Species. *ACS Catalysis* **2016**, *6* (4), 2473-2481. DOI: 10.1021/acscatal.6b00205.

(6) Platzman, I.; Brener, R.; Haick, H.; Tannenbaum, R. Oxidation of Polycrystalline Copper Thin Films at Ambient Conditions. *The Journal of Physical Chemistry C* **2008**, *112* (4), 1101-1108. DOI: 10.1021/jp076981k.
